# Supplementary material for: Intrauterine Device Training Workshop for Preclinical Medical Students
Source: MedEdPORTAL. 2019 Oct 18;15:10841. doi: 10.15766/mep_2374-8265.10841 (PMC6944262; doi:10.15766/mep_2374-8265.10841)
Supplement: Supplementary file 1 — A. Student Pretest Survey.docx B. IUD Simulation PowerPoint Didactic.pptx C. Student Posttest Survey.docx D. Faculty Guide for IUD Workshop.docx [file mep-15-10841-s001.zip › _Educational_Summary_Report_10841.pdf]

MedEdPORTAL® The AAMC Journal of Teaching and Learning Resources

Original Publication Open Access

Intrauterine Device Training Workshop for Preclinical Medical Students

Carlie Field, MD*, Lyndsey S. Benson, MD, MS, Alyssa Stephenson-Famy, MD, Sarah Prager, MD, MAS

*Corresponding author: csfield@uw.edu

Abstract

Introduction: Medical school reproductive health curricula often lack adequate education regarding intrauterine devices (IUDs). When placed in clinical scenarios, students may have insufficient knowledge and training to counsel patients about IUDs. **Methods:** We developed a workshop for preclinical medical students and assessed whether it improved knowledge of and comfort with counseling patients on IUDs. The workshop consisted of a 45-minute lecture and a 45-minute IUD simulation training. Each session was taught to groups of 40 to 50 students. The workshop was evaluated between January 2016 and November 2017. Participants completed pre- and postsurveys. The primary outcome was comfort level with IUD counseling. **Results:** One hundred forty-two students completed the workshop, and 137 completed both pre- and postsurveys (96% response rate). At baseline, more than half (56%, $n = 77$) had not seen an IUD inserted. Students scoring 75% or higher on the IUD knowledge questions increased from 51% ($n = 70$) on presurveys to 87% ($n = 119$) on postsurveys ($p < .0001$). Students agreeing or strongly agreeing that they felt comfortable counseling patients on IUDs increased from 27% ($n = 37$) to 92% ($n = 122$, $p < .0001$) on postsurveys. All students felt the workshop was worthwhile. **Discussion:** Preclinical students showed increased knowledge of and comfort with IUDs after a simple IUD simulation. Medical schools could utilize this workshop to ensure students have hands-on training and experience related to IUDs prior to clinical rotations and for their future careers.

Keywords
Long-Acting Reversible Contraception, Intrauterine Devices, Simulation, Family Medicine, OB/GYN, Pediatrics, Clinical/Procedural Skills Training

Educational Objectives

By the end of this activity, learners will be able to:

1. Identify different types of intrauterine contraception.
2. Compare the mechanism of action of different types of intrauterine contraception.
3. Discuss the risks and benefits of intrauterine contraception.
4. Increase the comfort level for counseling patients on intrauterine contraception.
5. Use the simulation setting to practice placement of intrauterine contraception.

Introduction

Unplanned, unwanted pregnancies are a public health problem in the United States.[1,2] The copper and levonorgestrel intrauterine devices (IUDs) are effective contraceptive methods with few side effects and rapid return to fertility upon discontinuation. IUDs have among the lowest failure rates, highest user satisfaction, and highest rates of continuation of all current contraceptive methods.[2-4] Although use of IUDs in the United States has increased, less effective methods, such as pills or condoms, are far more commonly used.[4-6] In 2013, only 7.7% of US women had ever used an IUD for contraception compared to 81.9% for contraceptive pills.[7] From 2006 to 2010, only 3.5% of women ages 15-44 years were currently using an IUD for contraception.[8] Increased use of IUDs can help reduce the number of unplanned pregnancies in the United States.[2]

Low utilization rates for IUDs may be due to factors including clinician lack of familiarity, misconceptions about IUDs, and low confidence with placement.[2] In a 2008 study, only 76% of US medical students reported that IUDs were covered in their medical school lectures, with the majority of lecture time being spent on less effective methods, such as oral contraceptive pills.[9] To bridge this gap, medical schools have begun to emphasize topics such as family planning and contraception in their curricula in an attempt to familiarize rising physicians. The tenth edition

Citation:
Field C, Benson LS, Stephenson-Famy A, Prager S. Intrauterine device training workshop for preclinical medical students. *MedEdPORTAL*. 2019;15:10841.
https://doi.org/10.15766/mep_2374-8265.10841

Copyright © 2019 Field et al. This is an open-access publication distributed under the terms of the Creative Commons Attribution license.

of the Association of Professors of Gynecology and Obstetrics (APGO) Medical Student Educational Objectives states that during their clinical clerkship, medical students should learn the steps to insert and remove an IUD and understand the risks, benefits, and mechanisms of action for various contraceptive methods, including IUDs.[10]

Simulation training has been shown to be an effective training tool for students and allows them a consequence-free environment to learn how to prevent mistakes prior to facing real patients.[11] Prior studies have shown that small-group procedural workshops are effective at improving medical students' confidence, participation, and performance with regard to procedural skills.[12-14] IUD simulation has been utilized effectively in the past for women's health trainees from several disciplines, including midwifery students, nurse practitioner students, family medicine residents, and OB/GYN residents.[15-17] A workshop using papaya models improved third-year medical students' knowledge of and comfort with IUD insertion and uterine aspiration.[18] In a small 2016 study, third-year medical students on their OB/GYN clerkship participated in didactics and hands-on stimulation using realistic pelvic models and demonstrated an increase in comfort with counseling patients on IUDs from 20% to 77%.[19]

Prior studies were almost exclusively targeted toward third- and fourth-year medical students during their OB/GYN clerkship rotations or resident physicians. Preclinical medical students were selected for this study because they are beginning to have more and more clinical experiences prior to entering their true clinical years. At the University of Washington, first- and second-year students spend one half-day per week in the preceptor primary care clinic. They are eager for training that links classroom learning with patient care. We also believe that increased exposure to IUDs over the medical school curriculum will increase students' long-term knowledge of and comfort with IUDs. Our study also included all the different types of IUDs currently on the market, which has not been done in prior studies. We sought to determine whether our intervention, a voluntary workshop including both didactic and hands-on simulation training for preclinical medical students, could improve students' comfort with counseling patients and placement of IUDs.

Methods

We developed this formal IUD workshop at the University of Washington School of Medicine in Seattle, Washington, after many years of family planning faculty members doing voluntary, informal IUD insertion and removal simulation with first- and second-year medical students based on requests from a variety of student groups on campus. The catalyst for this curriculum was preclinical student interest in contraceptive methods and the continued low use of long-acting reversible contraceptive (LARC) options despite their safety profile and patient acceptability compared to other methods. The formal simulation was a voluntary, after-hours workshop that included a 45-minute standardized didactic lecture on IUDs given by a family planning faculty member (Appendix B). Immediately following the didactic session, there was a detailed review on the steps of IUD insertion and a 45-minute hands-on simulation of insertion of IUDs using plastic discs in the shape of a uterus. The CuT 380A (Paragard, CooperSurgical, Trumbell, Connecticut) and the levonorgestrel-containing IUDs (Mirena, Kyleena, and Skyla, Bayer HealthCare Pharmaceuticals, Inc., Whippany, New Jersey; Liletta, Allergan/Medicines 360, San Francisco, California) were utilized. Each student was provided with a simulation uterus disc and demo IUDs for insertion. Circulating volunteer resident and faculty preceptors were available to assist students with insertions. After the didactic and hands-on simulation (Appendix B), the postsurvey was administered (Appendix C).

Our evaluation of the workshop was performed between January 2016 and November 2017. These sessions occurred three times during the study period, and participants included preclinical medical students entering medical school from 2014 through 2017. Participation in the study was voluntary, and participants were given a cover letter at the beginning of the session explaining the study. Consent was implied with the completion of the pre- and postintervention surveys (Appendices A and C). Students were eligible to participate in the study if they attended the IUD didactic session and hands-on simulation, were currently enrolled first- or second-year medical students, and completed both the pre- and postsurvey. Students were ineligible if they did not meet these criteria or had previously participated in the workshop. The survey questions were designed to assess preclinical medical students' knowledge of and comfort with IUDs both before and after the session. Many of the survey questions used were modified with author permission from a prior study of medical students' knowledge of and comfort with IUDs.[19]

Equipment

During the simulation, each student had an individual plastic uterus model and IUD demonstration kit. The IUD demonstration kits were donated by the respective pharmaceutical companies after communicating with pharmaceutical representatives regarding the goals and objectives of this workshop. Each kit contained a plastic uterus model that the student could use for

Copyright © 2019 Field et al. This is an open-access publication distributed under the terms of the Creative Commons Attribution license.

the session. The number of models and IUDs required depended on the number of learners present. The scissors, uterine sounds, and tenaculums could be shared among students. Equipment included the following:

- Plastic uterus model provided by IUD manufacturer.
- IUD demonstration kit provided by IUD manufacturer:
  - CuT 380A (Paragard).
  - Levonorgestrel-containing IUDs (Mirena, Kyleena, Skyla, and Liletta).
- Long suture scissors.
- Uterine sound.
- Tenaculum.
- Gloves.

Personnel
Personnel included faculty and resident physicians from the OB/GYN and family medicine departments who were rotating and working one-on-one with students and their individual uterus model. Appendix D presents a faculty guide to conducting the workshop.

Simulation
After the didactic session, each student was provided with an IUD demonstration kit, gloves, tenaculum, and suture scissors at the workstation. To ensure that students had experience with the different types of IUDs, demonstration kits were alternated between students so that they could switch during the session. Facilitators were assigned to rows of students to assist and provide one-on-one assistance during the IUD insertion. Students were then asked to follow the IUD insertion steps listed in the PowerPoint slides, including performing a mock bimanual examination, placing a tenaculum, sounding the uterus, placing the IUD, and then trimming the strings. During the hands-on portion, faculty and resident facilitators answered questions and helped guide IUD insertions. Students then switched IUD types among each other so that they had the opportunity to practice inserting Mirena, Kyleena, Skyla, Liletta, and Paragard IUDs.

Assessment
Prior to the intervention, all participants were given a paper presurvey (Appendix A) with questions related to the following: (1) intended medical specialty, (2) baseline IUD knowledge, (3) baseline IUD experiences and exposures, (4) main source of information on IUDs, and (5) number of IUDs they had seen placed. In the final workshop in November 2017 ($n = 46$), the presurvey also included questions about age and gender for additional demographic information.

Both pre- and postsurveys included questions on the following: (1) knowledge related to IUD mechanism of action and return to fertility; (2) candidates for IUDs specifically related to parity, age, and sexually transmitted infections; (3) comfort level with counseling patients on IUDs; and (4) comfort level with placement of IUDs. Each participant created a unique four-digit number that was used to pair the pre- and postsurveys.

Analysis was limited to eligible first- and second-year participants with completed pre- and postsurveys. The primary outcome of interest was participant comfort level with IUD counseling pre- and postintervention using a 5-point Likert scale. The responses were dichotomized into agree (including "strongly agree" and "agree") or disagree (including "neutral," "disagree," and "strongly disagree"). For knowledge questions, scores were dichotomized using a cutoff of 75% correct on pre- and postsurveys. The McNemar test was used to compare pre- and postsurvey dichotomous variables. Descriptive analyses were performed on demographic data, intended medical specialty, and prior IUD experiences and exposures. The University of Washington Institutional Review Board found this study to be exempt. Study data were managed in REDCap (Research Electronic Data Capture) hosted at the University of Washington.[20] Data analysis was performed with Stata/SE 13.1 (College Station, Texas).

Results

One hundred fifty students participated in the three workshops that occurred during the study period. Of these participants, eight students were excluded who did not indicate their year in training or who were not preclinical medical students. Of the 142 students who indicated they were in their first or second year, 137 completed paired pre- and postsurveys, for a response rate of 96%. We estimate that the total number of Seattle-based eligible first- and second-year medical students at the University of Washington over the three sessions was 573, and thus, this study involved 24% of all medical students during the study time frame.

Fifty-eight percent of the students who participated were first-year medical students, and 42% were second-year medical students. Additional demographics obtained from students in the last workshop ($n = 46$) showed that participants were an average age of 25 years (range: 22-36) and 85% ($n = 39$) identified as female. Participants most commonly reported an interest in specializing in family medicine (29%, $n = 39$), internal medicine (20%, $n = 28$), OB/GYN (14%, $n = 19$), pediatrics (13%, $n = 18$), emergency medicine (10%, $n = 13$), or general surgery (5%, $n = 7$).

Copyright © 2019 Field et al. This is an open-access publication distributed under the terms of the Creative Commons Attribution license.

On the presurvey, all students (100%) strongly agreed or agreed that they had an interest in learning more about IUDs, and 82% ($n = 113$) reported that contraceptive counseling would be a part of their future medical practice. Participants had high rates of familiarity with IUDs, with almost all participants (89%, $n = 122$) reporting they knew a family member or friend with an IUD and 42% ($n = 57$) reporting they had an IUD themselves or a partner with one. Eighty-two percent of participants ($n = 112$) would recommend an IUD to a family member (reporting “strongly agree” or “agree”). Most participants reported that prior knowledge was obtained from a health care provider (42%, $n = 63$), friends (23%, $n = 34$), medical school lecture (10%, $n = 15$), family (6%, $n = 9$), and the internet (5%, $n = 8$). Despite these exposures and opinions related to IUDs, the majority of students (56%, $n = 77$) had never seen an IUD placed in a patient.

Baseline knowledge regarding IUDs was modest, with 51% ($n = 70$) correctly answering 75% or more of the questions. After the intervention, there was a significant increase in correct answers to knowledge questions, with 87% ($n = 119$) of students answering at least 75% correct ($p < .0001$). Similarly, participants were more likely to recommend IUDs to appropriate candidates after the intervention (Table 1).

After the intervention, students showed significantly higher comfort scores with IUD counseling, IUD placement steps, IUD placement in a plastic model, IUD placement in a patient, and teaching IUD insertion to another student (Table 2). Before the intervention, only 27% ($n = 37$) of participants reported “strongly agree” or “agree” when asked about their comfort with counseling on IUDs. After the intervention, comfort with IUD counseling was 92% ($n = 122$, $p < .0001$). The intervention was well received, with 100% of respondents agreeing that the workshop was worthwhile.

Discussion

This IUD simulation and the associated evaluation demonstrated that first- and second-year medical students who voluntarily participated in a standardized IUD simulation training significantly improved their knowledge of and comfort with IUD counseling and placement. The intervention was also thought to be worthwhile by the participants.

Table 1. Patients to Whom You Would Recommend Intrauterine Device Placement[a]

Patient Type	Preintervention *n* (%)	Postintervention *n* (%)	*p*
Nulliparous woman	127 (93)	131 (98)	.07
Multiparous woman	111 (82)	128 (96)	.0001
Teenager	109 (80)	131 (98)	<.0001
Woman with chlamydia	25 (18)	78 (59)	<.0001

[a]Reported as proportion of participants who answered “strongly agree” or “agree” on a 5-point Likert scale.

Table 2. Comfort With IUD Placement[a]

Action	Preintervention *n* (%)	Postintervention *n* (%)	*p*
Counseling on IUD	37 (27)	122 (92)	<.0001
IUD placement steps	18 (14)	126 (95)	<.0001
Placement of IUD in plastic model	5 (4)	112 (84)	<.0001
Teaching IUD placement to another student	4 (3)	113 (85)	<.0001
IUD in patient	9 (7)	77 (58)	<.0001

Abbreviation: IUD, intrauterine device.
[a]Reported as proportion of participants who answered “strongly agree” or “agree” on a 5-point Likert scale.

Prior studies have shown that IUD simulation improves knowledge of and comfort with placement of IUDs.[15,16,18] A recent study comparing high- and low-fidelity simulators showed that knowledge of and comfort with IUD placement were comparable in both groups.[17] To our knowledge, ours is the first study looking at IUD simulation, knowledge, and comfort in first- and second-year medical students. Our findings are consistent with previously published results in OB/GYN clerkship students.[18] With limited time in the structured classroom curriculum and limited hands-on training during medical school, other modalities to ensure attainment of APGO’s objectives should be used for medical student education.[10] Simple, low-fidelity simulation can be used with excellent results for student learning.[17]

Simulation should not be limited to the clinical years of medical school but instead should be incorporated throughout the preclinical curriculum, with opportunities for multiple exposures and learning opportunities. The preclinical years are an appropriate time to introduce this workshop, as many medical schools now have a longitudinal primary care clerkship that spans the preclinical years. Students have the opportunity to see and help manage contraception, including LARC, from the first day of medical school. This type of workshop may also be beneficial for fourth-year medical students going into a women’s health care field. The simulation could be included in the capstone or transition to residency curriculum at the end of medical school, for those students who are interested. This would offer a nice way to consolidate their knowledge and skills just prior to entering residency training.

Considering that many medical schools across the country have condensed preclinical course work, it may be challenging to

Copyright © 2019 Field et al. This is an open-access publication distributed under the terms of the Creative Commons Attribution license.

make this type of workshop mandatory for all medical students during the required time. An evening session or after-class session may be the best option for students to continue these types of simulation learning opportunities. We were able to attain a large sample size for this study likely because it was held on the medical school campus, where many students tend to work and study after required classes. The evening simulation session worked well for our study and was able to draw a large number of students as it was combined with an already existing evening medical student interest group meeting. As students enter into the third and fourth years of medical school, rotations can be located at a variety of hospitals across a city or even in different states, and scheduling large groups of students for simulations and/or workshops tends to be more difficult.

Major strengths of this study include the large number of participants and high response rate. The high response rate was likely in part due to the use of paper surveys just prior to and after the session. We suspect that our response rate would have been much lower if an online survey had been utilized, as students are likely to delete or ignore a survey sent to them by email. The students reported diverse interests in future medical specialization. This type of simulation workshop is easily reproducible at other institutions with all types of learners (medical students, nursing students, etc.). Similarly, this simulation workshop is inexpensive given the use of donated IUDs and plastic uterus discs.

There are several limitations to this study. It was performed at a single large academic center and may not be generalizable to all training sites. We had sufficient donations of IUD kits so that each student could perform the IUD placement individually; however, at smaller institutions, this simulation could easily be modified and performed in a small-group setting where students rotate through the IUD insertion while also watching their peers perform the exercise. If IUD kits with plastic uterus discs are unavailable, the simulation could be modified to use a papaya model, as described previously.[18] Participation in this study was voluntary and outside of regular school hours. Therefore, participants likely had more interest in and experience with IUDs and thus higher baseline knowledge surrounding IUDs compared to other first- and second-year medical students who chose not to participate. In the future, it is our hope that this type of training will be incorporated into the structured curriculum for preclinical students so that all students will have the ability to participate. Participants were surveyed immediately after the intervention, but no long-term follow-up was performed; thus, it is difficult to know if this intervention will provide long-lasting knowledge and improve future clinical care. Future studies could look at retention of knowledge in students by performing repeat surveys at longer intervals. Finally, to better prepare faculty for this workshop, it would be helpful to provide a brief orientation of the simulation and how faculty can best assist the students to ensure a positive experience. There would be minimal preparation time for instructors who place LARC in their clinical practice as the uterus models are simple and the IUD insertion models are high fidelity and closely mimic the real applicators.

Despite these limitations, this study indicates that a simple simulation for IUDs can be used for first- and second-year medical students to improve knowledge of and comfort with counseling and placement of IUDs. Future study and utilization of a low-tech simulation workshop for IUDs should be considered not only in other academic settings, as in this study, but also in community hospitals or in the developing world.

Appendices

A. Student Pretest Survey.docx

B. IUD Simulation PowerPoint Didactic.pptx

C. Student Posttest Survey.docx

D. Faculty Guide for IUD Workshop.docx

All appendices are peer reviewed as integral parts of the Original Publication.

Carlie Field, MD: Resident Physician, Department of Obstetrics and Gynecology, University of Washington Medical Center

Lyndsey S. Benson, MD, MS: Assistant Professor, Department of Obstetrics and Gynecology, University of Washington Medical Center

Alyssa Stephenson-Famy, MD: Associate Professor, Department of Obstetrics and Gynecology, University of Washington Medical Center

Sarah Prager, MD, MAS: Professor, Department of Obstetrics and Gynecology, University of Washington Medical Center

Acknowledgments

Demonstration kits were provided by the pharmaceutical companies for the CuT 380A (Teva Pharmaceuticals, North Wales, Pennsylvania) and the levonorgestrel-containing intrauterine devices (Mirena, Kyleena, and Skyla, Bayer HealthCare Pharmaceuticals, Inc., Whippany, New Jersey; Liletta, Allergan/Medicines 360, San Francisco, California).

We would like to thank Dr. Deborah Bartz for allowing us to use portions of a previously published survey related to intrauterine contraception.

Copyright © 2019 Field et al. This is an open-access publication distributed under the terms of the Creative Commons Attribution license.

Disclosures
None to report.

Funding/Support
None to report.

Prior Presentations
This research was presented at the 2018 CREOG/APGO Annual Meeting on March 1, 2018, in Washington, DC.

Ethical Approval
The University of Washington Institutional Review Board approved this study.

References

1. Trussell J, Hassan F, Lowin J, Law A, Filonenko A. Achieving cost-neutrality with long-acting reversible contraceptive methods. *Contraception*. 2015;91(1):49-56. https://doi.org/10.1016/j.contraception.2014.08.011
2. Lunde B, Smith P, Grewal M, Kumaraswami T, Cowett A, Harwood B. Long acting contraception provision by rural primary care physicians. *J Womens Health (Larchmt)*. 2014;23(6):519-524. https://doi.org/10.1089/jwh.2013.4286
3. Fleming KL, Sokoloff A, Raine TR. Attitudes and beliefs about the intrauterine device among teenagers and young women. *Contraception*. 2010;82(2):178-182. https://doi.org/10.1016/j.contraception.2010.02.020
4. Peipert JF, Zhao Q, Allworth JE, et al. Continuation and satisfaction of reversible contraception. *Obstet Gynecol*. 2011;117(5):1105-1113. https://doi.org/10.1097/AOG.0b013e31821188ad
5. Finer LB, Jerman J, Kavanaugh ML. Changes in use of long-acting contraceptive methods in the United States, 2007–2009. *Fertil Steril*. 2012;98(4):893-897. https://doi.org/10.1016/j.fertnstert.2012.06.027
6. Kittur ND, Secura GM, Peipert JF, Madden T, Finer LB, Allsworth JE. Comparison of contraceptive use between the Contraceptive CHOICE Project and state and national data. *Contraception*. 2011;83(5):479-485. https://doi.org/10.1016/j.contraception.2010.10.001
7. Daniels K, Mosher WD, Jones J. *Contraceptive Methods Women Have Ever Used: United States, 1982–2010*. Hyattsville, MD: National Center for Health Statistics; 2013. National Health Statistics Report no. 62.
8. Jones J, Mosher W, Daniels K. *Current Contraceptive Use in the United States, 2006–2010, and Changes in Patterns of Use Since 1995*. Hyattsville, MD: National Center for Health Statistics; 2012. National Health Statistics Report no. 60.
9. Steinauer J, Landy U, Filippone H, Laube D, Darney PD, Jackson RA. Predictors of abortion provision among practicing obstetrician-gynecologists: a national survey. *Am J Obstet Gynecol*. 2008;198(1):39.e1-39.e6. https://doi.org/10.1016/j.ajog.2007.06.002
10. *APGO Medical Student Educational Objectives*. 10th ed. Crofton, MD: Association of Professors of Gynecology and Obstetrics; 2014.
11. Jude DC, Gilbert GG, Magrane D. Simulation training in the obstetrics and gynecology clerkship. *Am J Obstet Gynecol*. 2006;195(5):1489-1492. https://doi.org/10.1016/j.ajog.2006.05.003
12. Fernando N, McAdam T, Cleland J, Yule S, McKenzie H, Youngson G. How can we prepare medical students for theatre-based learning? *Med Educ*. 2007;41(10):968-974. https://doi.org/10.1111/j.1365-2923.2007.02839.x
13. Brunt LM, Halpin VJ, Klingensmith ME, et al. Accelerated skills preparation and assessment for senior medical students entering surgical internship. *J Am Coll Surg*. 2008;206(5):897-904. https://doi.org/10.1016/j.jamcollsurg.2007.12.018
14. Leopold SS, Morgan HD, Kadel NJ, Gardner GC, Schaad DC, Wolf FM. Impact of educational intervention on confidence and competence in the performance of a simple surgical task. *J Bone Joint Surg Am*. 2005;87(5):1031-1037. https://doi.org/10.2106/JBJS.D.02434
15. Khadivzadeh T, Erfanian F. The effects of simulated patients and simulated gynecologic models on student anxiety in providing IUD services. *Simul Healthc*. 2012;7(5):282-287. https://doi.org/10.1097/SIH.0b013e31826064b7
16. Goldthwaite LM, Sheeder J, Teal SB, Tocce KM. Comfort with skills and knowledge after immediate postpartum intrauterine device training. *Obstet Gynecol*. 2016;128(suppl 1):6S-11S. https://doi.org/10.1097/AOG.0000000000001650
17. Nippita S, Haviland MJ, Voit SF, Perez-Peralta J, Hacker MR, Paul ME. Randomized trial of high- and low-fidelity simulation to teach intrauterine contraception placement. *Am J Obstet Gynecol*. 2018;218(2):258.e1-258.e11. https://doi.org/10.1016/j.ajog.2017.11.553
18. Steinauer J, Preskill F, Devaskar S, Landy U, Darney P. The papaya workshop: using the papaya to teach intrauterine gynecologic procedures. *MedEdPORTAL*. 2013;9:9388. https://doi.org/10.15766/mep_2374-8265.9388
19. Bartz D, Paris A, Maurer R, Gardner R, Johnson N. Medical student simulation training in intrauterine contraception insertion and removal: an intervention to improve comfort, skill, and attitudes. *Contracept Reprod Med*. 2016;1:3. https://doi.org/10.1186/s40834-016-0009-2
20. Harris PA, Taylor R, Thielke R, Payne J, Gonzalez N, Conde JG. Research Electronic Data Capture (REDCap)—a metadata-driven methodology and workflow process for providing translational research informatics support. *J Biomed Inform*. 2009;42(2):377-381. https://doi.org/10.1016/j.jbi.2008.08.010

Received: November 8, 2018
Accepted: June 11, 2019
Published: October 18, 2019

Copyright © 2019 Field et al. This is an open-access publication distributed under the terms of the Creative Commons Attribution license.
