# Supplementary material for: Intrauterine Device Training Workshop for Preclinical Medical Students
Source: MedEdPORTAL. 2019 Oct 18;15:10841. doi: 10.15766/mep_2374-8265.10841 (PMC6944262; doi:10.15766/mep_2374-8265.10841)
Supplement: Supplementary file 1 — A. Student Pretest Survey.docx B. IUD Simulation PowerPoint Didactic.pptx C. Student Posttest Survey.docx D. Faculty Guide for IUD Workshop.docx [file mep-15-10841-s001.zip › A. Student Pretest Survey.docx]

**Pre-Training Intrauterine Contraception Questionnaire**

Please provide the last 4 digits of your cell phone (or home phone) number. _____________.

*This is used to pair your pre- and post- survey responses. It is not used to identify you in any way.*

1. Have you previously taken this course or filled out this survey? YES NO (Please circle one)

IF YOU ANSWERED YES TO THE ABOVE QUESTION, PLEASE DO NOT COMPLETE THE REMAINING QUESTIONS ON THIS SURVEY, THANK YOU!

1. Please circle your year in medical school: FIRST YEAR SECOND YEAR (Please circle one)
2. What is your current age: ________ years
3. What is your gender: ________________
4. If you had to choose a specialty today, which one would you be most likely to pick? (Please circle one)
   1. Family Medicine
   2. Pediatrics
   3. OB-GYN
   4. Internal Medicine
   5. Emergency Medicine
   6. Other: ___________
5. What is your main source of information about IUDs? (Please circle one)
   1. Medical school lecture
   2. Friends
   3. Family
   4. Healthcare provider
   5. Other: ____________
6. How many IUD placement procedures have you seen in a clinical setting? (Place one check mark)

___ (0)

___ (1-2)

___ (3-4)

___ (>5)

1. The following statements have to do with your personal experiences with IUDs. (Circle YES or NO)
   1. I or my partner has an IUD, or have had one in the past.

YES NO

- 1. I know of at least one friend or family member that has ever had an IUD

YES NO

1. If 100 women used any type of IUD for 1 year, how many would have an unintended pregnancy?
   1. <1%
   2. 1-2%
   3. 3-5%
   4. >5%
2. How quickly does fertility typically return after any type of IUD is removed?
   1. Within 1 month
   2. 1-2 months
   3. 3-6 months
   4. 6-12 months
3. What is the primary mechanism of action of a levonorgestrel IUD in contraception?
   1. preventing fertilization
   2. preventing implantation
   3. disrupting an implanted embryo
4. What is the primary mechanism of action of a copper IUD in contraception?
   1. preventing fertilization
   2. preventing implantation
   3. disrupting an implanted embryo

Intrauterine Contraception Questionnaire

|  | 1 | 2 | 3 | 4 | 5 |
| --- | --- | --- | --- | --- | --- |
|  | Strongly Agree | Agree | Neither Agree nor Disagree | Disagree | Strongly Disagree |
| **The following clinicians should be able to COUNSEL patient about IUDs:** |  |  |  |  |  |
| Ob-Gyns |  |  |  |  |  |
| Family Physicians |  |  |  |  |  |
| Internal Medicine Physicians |  |  |  |  |  |
| Pediatricians |  |  |  |  |  |
| Midwives/Nurse Practitioners |  |  |  |  |  |

|  | 1 | 2 | 3 | 4 | 5 |
| --- | --- | --- | --- | --- | --- |
|  | Strongly Agree | Agree | Neither Agree nor Disagree | Disagree | Strongly Disagree |
| **The following clinicians should be able to PLACE IUDs:** |  |  |  |  |  |
| Pediatricians |  |  |  |  |  |
| Midwives/Nurse Practitioners |  |  |  |  |  |
| Internal Medicine  Physicians |  |  |  |  |  |
| Ob-Gyns |  |  |  |  |  |
| Family Physicians |  |  |  |  |  |

|  | 1 | 2 | 3 | 4 | 5 |
| --- | --- | --- | --- | --- | --- |
|  | Strongly Agree | Agree | Neither Agree nor Disagree | Disagree | Strongly Disagree |
| I am interested in learning more about IUDs |  |  |  |  |  |
| I want to learn how to place IUDs |  |  |  |  |  |
| Contraceptive counseling will be a part of my practice |  |  |  |  |  |
| I would recommend an IUD to my family member |  |  |  |  |  |
| **Assuming she is an otherwise good candidate, I would recommend an IUD for a patient:** |  |  |  |  |  |
| Who has never been pregnant |  |  |  |  |  |
| Who is under 19 years old |  |  |  |  |  |
| Who currently has chlamydia |  |  |  |  |  |
| Who has had more than 1 vaginal delivery |  |  |  |  |  |

|  | 1 | 2 | 3 | 4 | 5 |
| --- | --- | --- | --- | --- | --- |
|  | Strongly Agree | Agree | Neither Agree nor Disagree | Disagree | Strongly Disagree |
| I feel confident that am able to counsel patient about the IUD |  |  |  |  |  |
| I know as much about the IUD as other contraceptive methods |  |  |  |  |  |
| I know the steps to place an IUD |  |  |  |  |  |
| I feel comfortable placing an IUD independently in plastic model |  |  |  |  |  |
| I could teach another student how to place an IUD in a plastic model |  |  |  |  |  |
| I feel comfortable placing an IUD in a patient under faculty supervision |  |  |  |  |  |

END OF PRE-TEST

*With permission from the authors, portions of this survey have been used from a previously published manuscript (Bartz, et al).
