# Supplementary material for: Intrauterine Device Training Workshop for Preclinical Medical Students
Source: MedEdPORTAL. 2019 Oct 18;15:10841. doi: 10.15766/mep_2374-8265.10841 (PMC6944262; doi:10.15766/mep_2374-8265.10841)
Supplement: Supplementary file 1 — A. Student Pretest Survey.docx B. IUD Simulation PowerPoint Didactic.pptx C. Student Posttest Survey.docx D. Faculty Guide for IUD Workshop.docx [file mep-15-10841-s001.zip › B. IUD Simulation PowerPoint Didactic.pptx]

## Slide 1
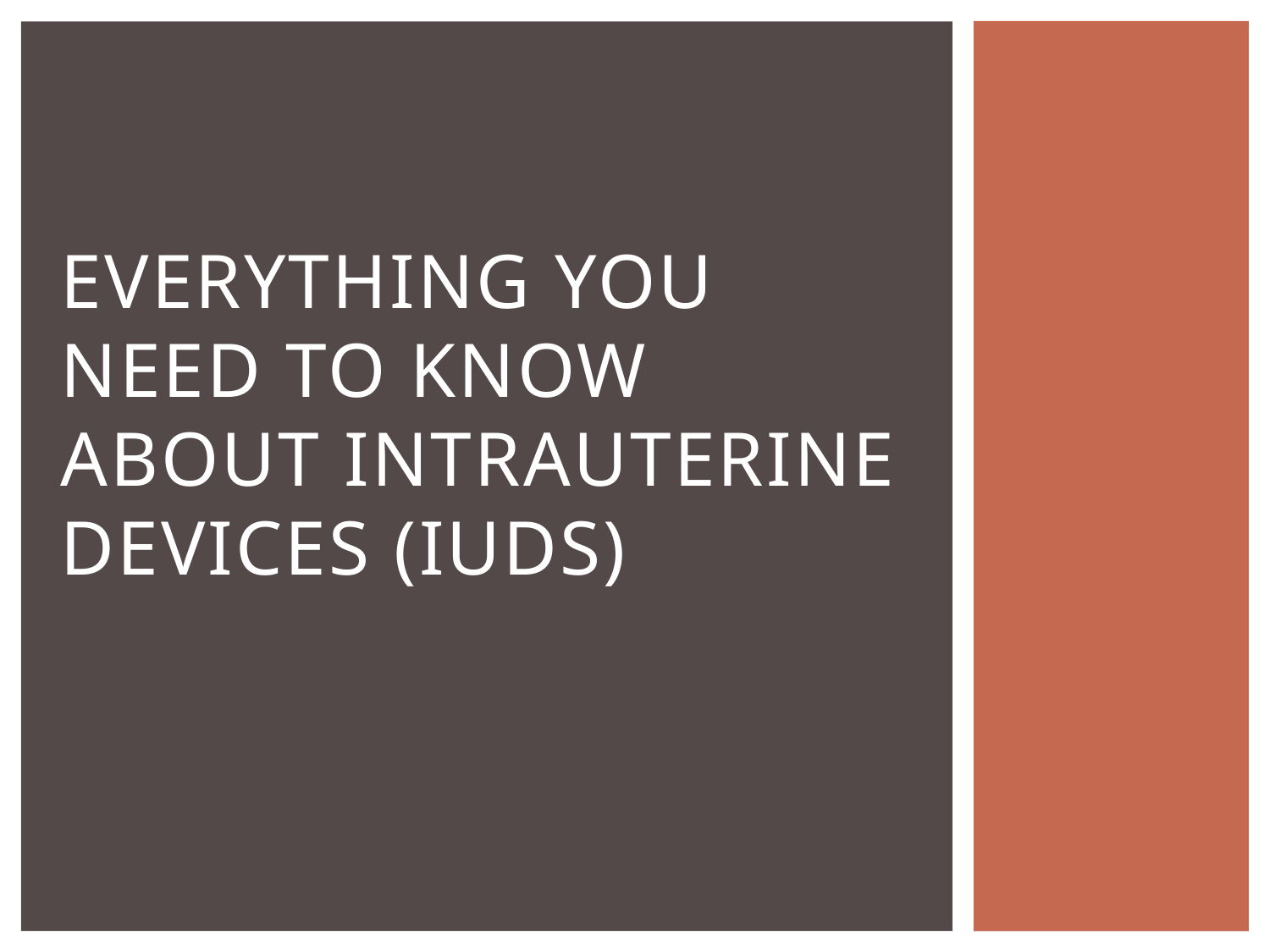

# Everything you need to know about intrauterine devices (iuds)

## Slide 2
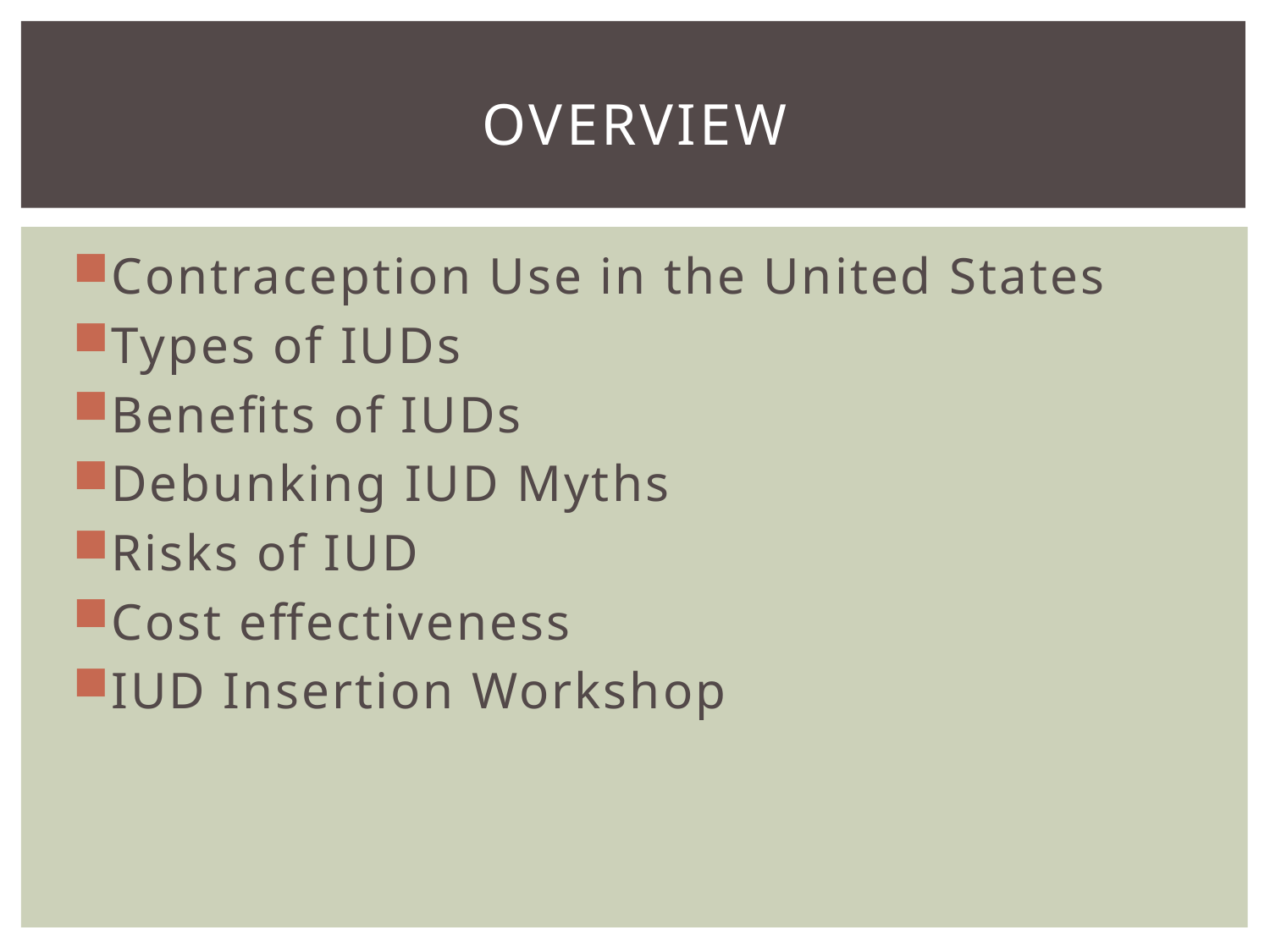

# Overview
Contraception Use in the United States
Types of IUDs
Benefits of IUDs
Debunking IUD Myths
Risks of IUD
Cost effectiveness
IUD Insertion Workshop

## Slide 3
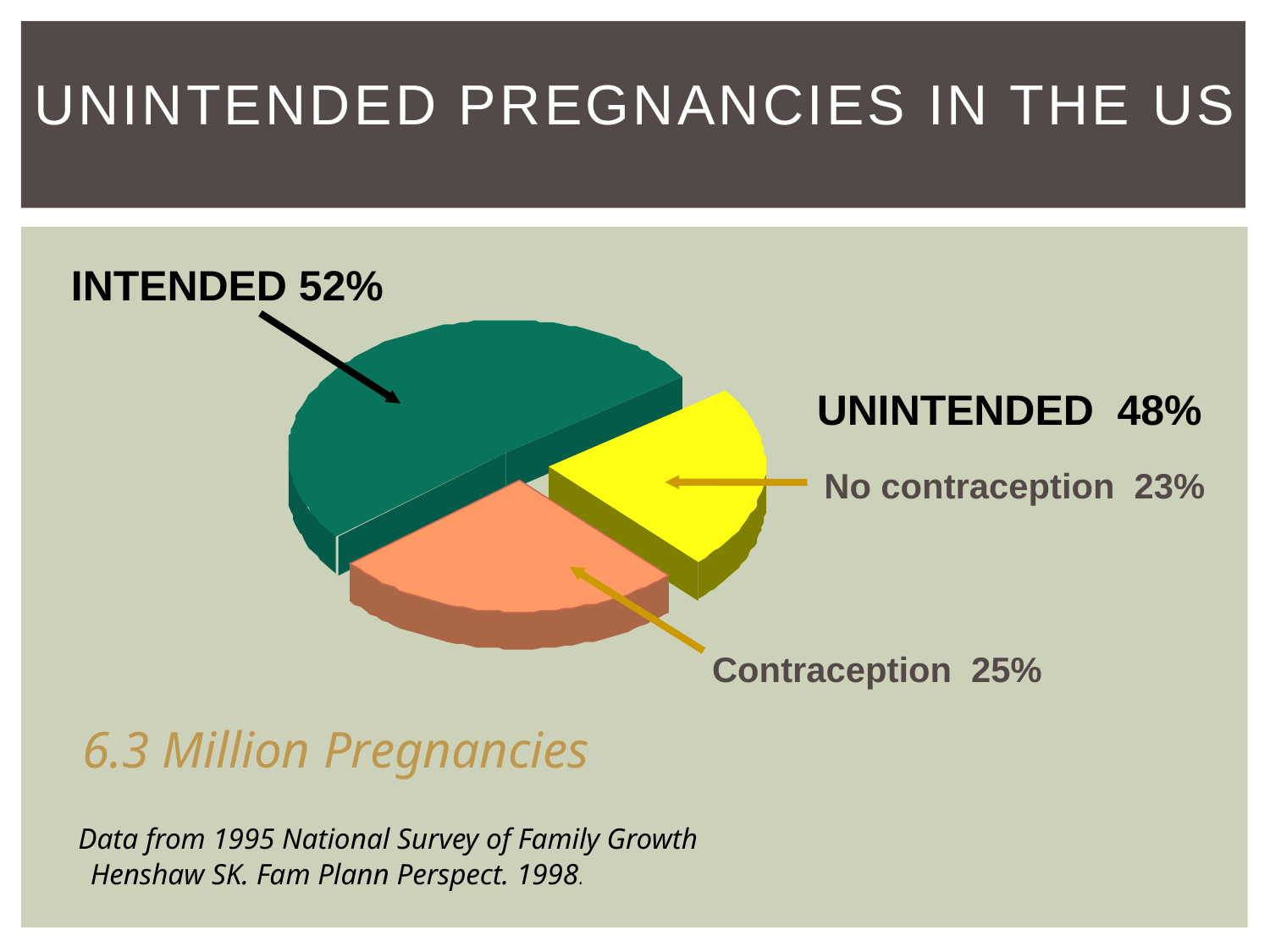

# Unintended Pregnancies in the US
INTENDED 52%
UNINTENDED 48%
No contraception 23%
Contraception 25%
6.3 Million Pregnancies
Data from 1995 National Survey of Family Growth
Henshaw SK. Fam Plann Perspect. 1998.

## Slide 4
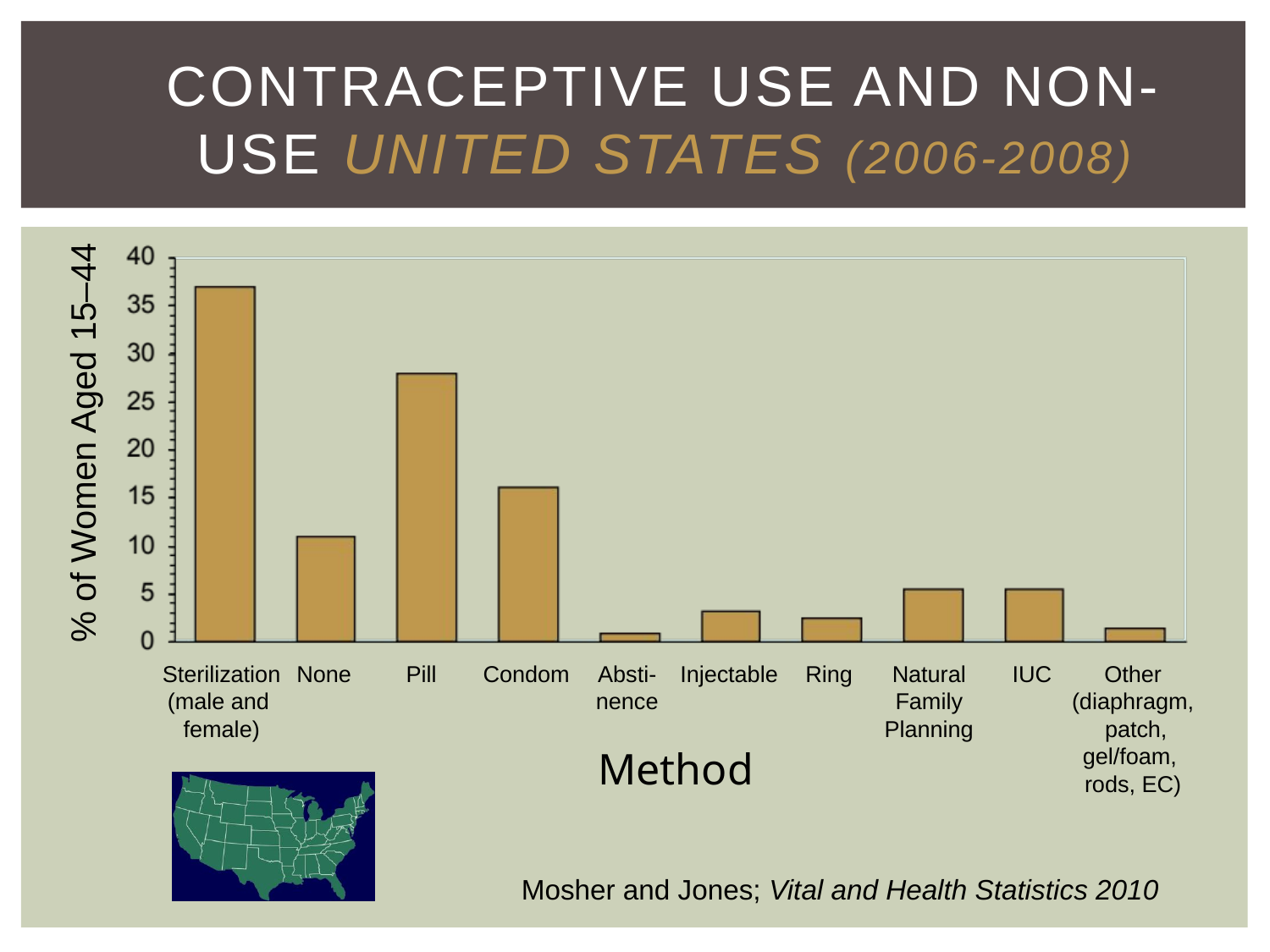

# Contraceptive Use and Non-Use United States (2006-2008)
% of Women Aged 15–44
Sterilization
(male and
female)
None
Pill
Condom
Absti-
nence
Injectable
Ring
Natural
Family
Planning
IUC
Other
(diaphragm,
 patch,
gel/foam,
rods, EC)
Method
Mosher and Jones; Vital and Health Statistics 2010

## Slide 5
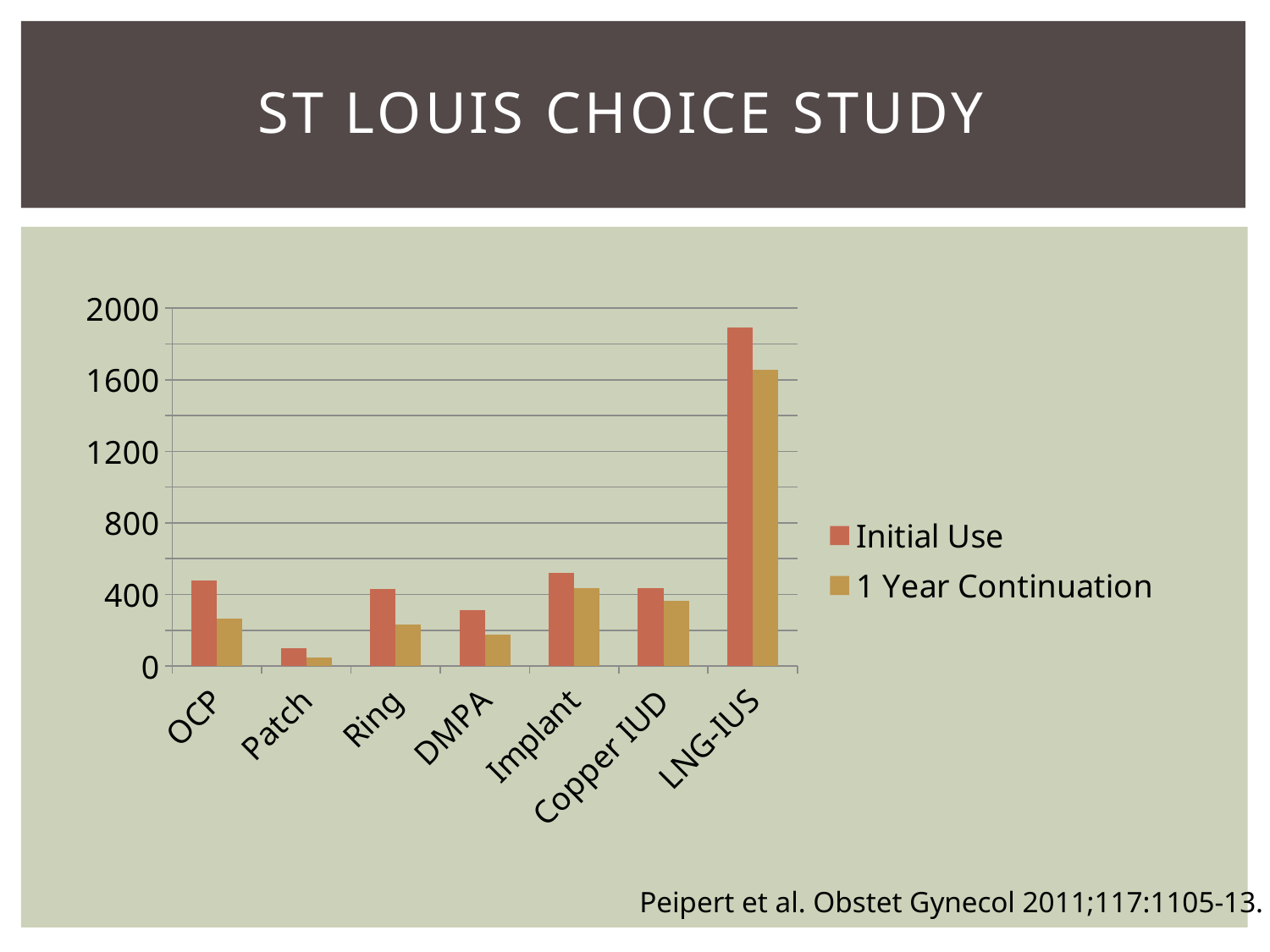

# St Louis CHOICE Study
### Chart
| Category | Initial Use | 1 Year Continuation |
|---|---|---|
| OCP | 478.0 | 263.0 |
| Patch | 99.0 | 49.0 |
| Ring | 431.0 | 234.0 |
| DMPA | 313.0 | 177.0 |
| Implant | 522.0 | 435.0 |
| Copper IUD | 434.0 | 365.0 |
| LNG-IUS | 1890.0 | 1654.0 |Peipert et al. Obstet Gynecol 2011;117:1105-13.

## Slide 6
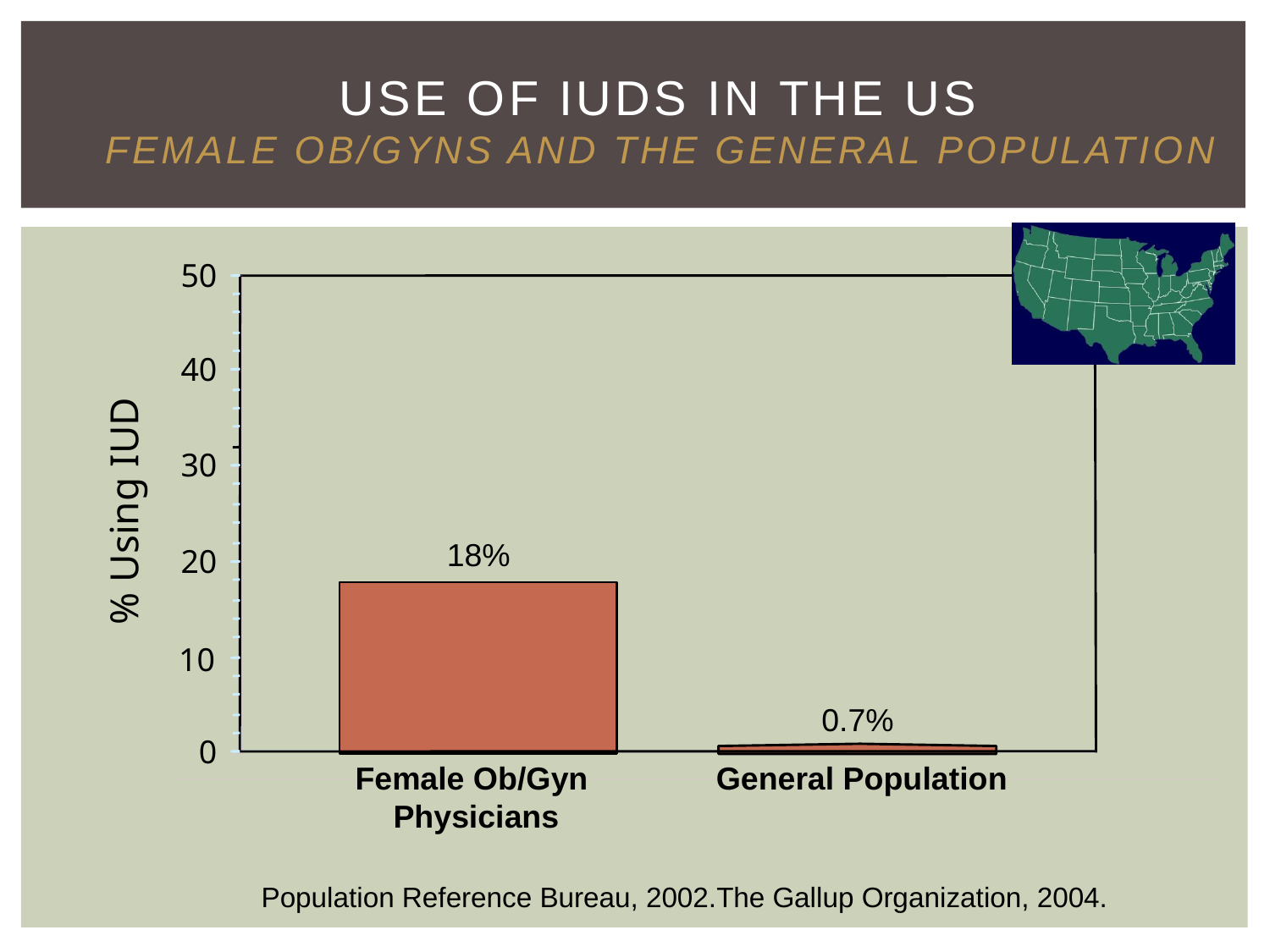

# Use of IUDs in the US Female Ob/Gyns and the General Population
50
40
30
% Using IUD
18%
20
10
0.7%
0
Female Ob/Gyn
Physicians
General Population
Population Reference Bureau, 2002.The Gallup Organization, 2004.

## Slide 7
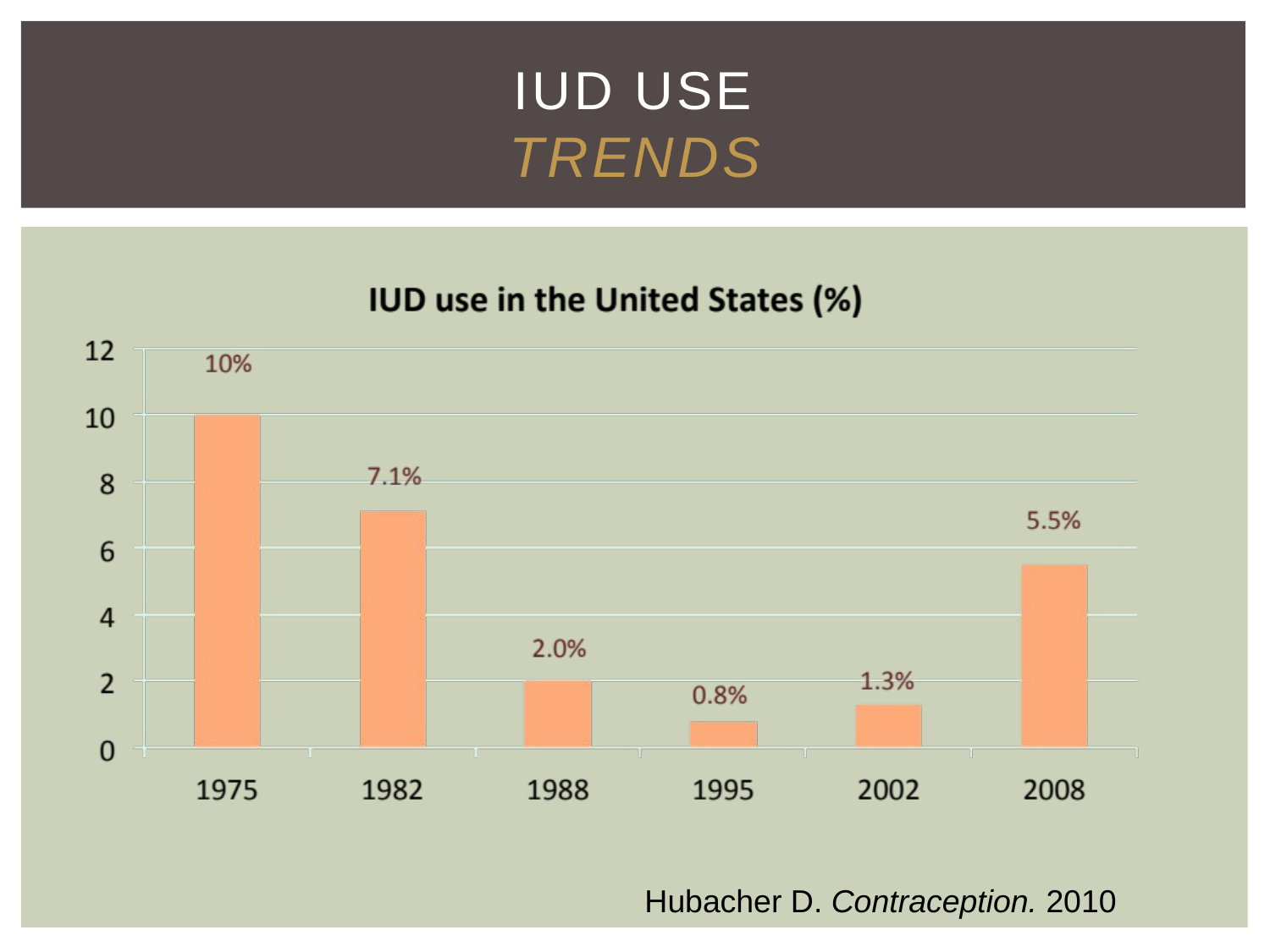

# IUD Use Trends
Hubacher D. Contraception. 2010

## Slide 8
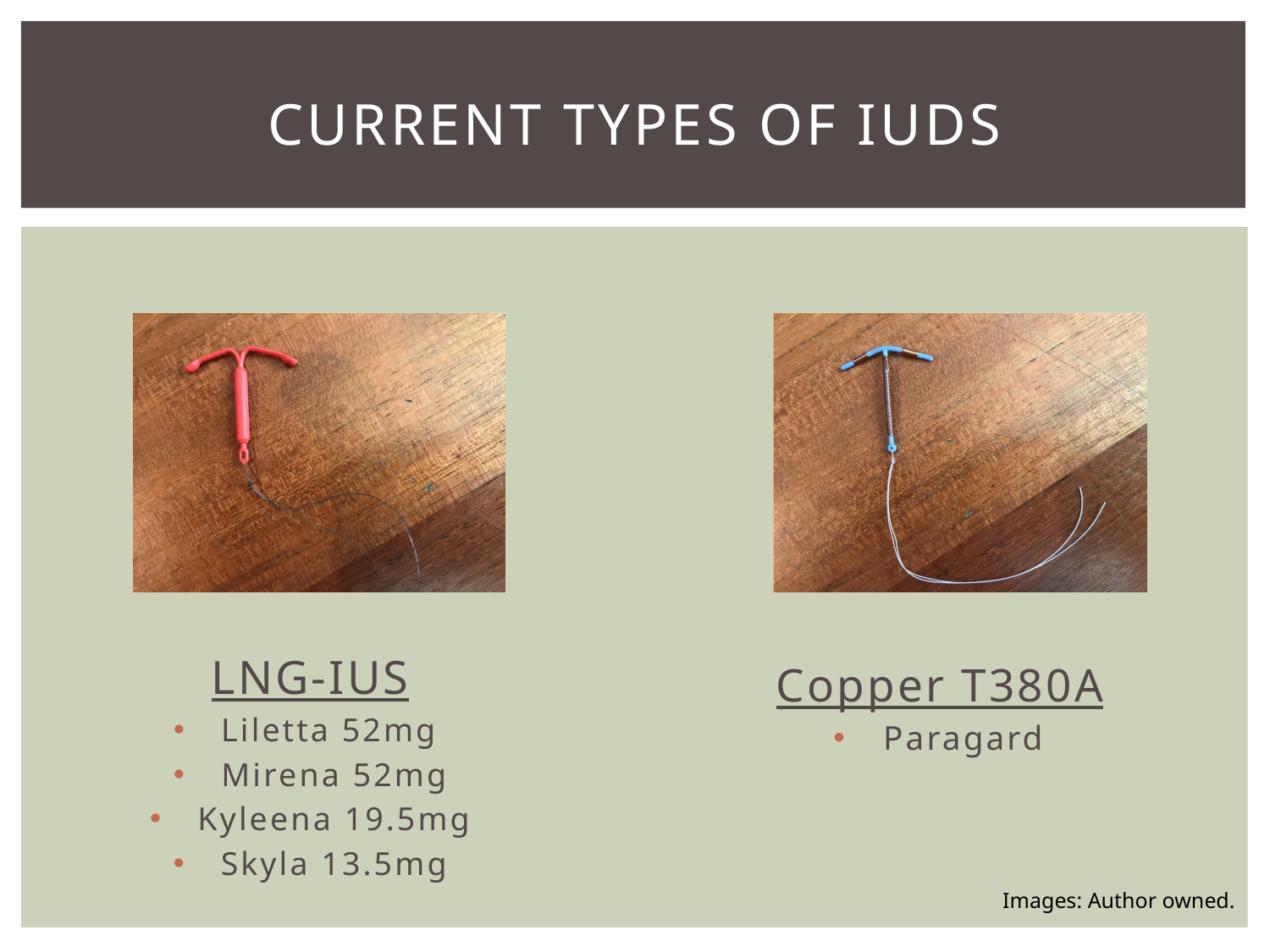

# Current Types of IUDs
Copper T380A
Paragard
LNG-IUS
Liletta 52mg
Mirena 52mg
Kyleena 19.5mg
Skyla 13.5mg
Images: Author owned.

## Slide 9
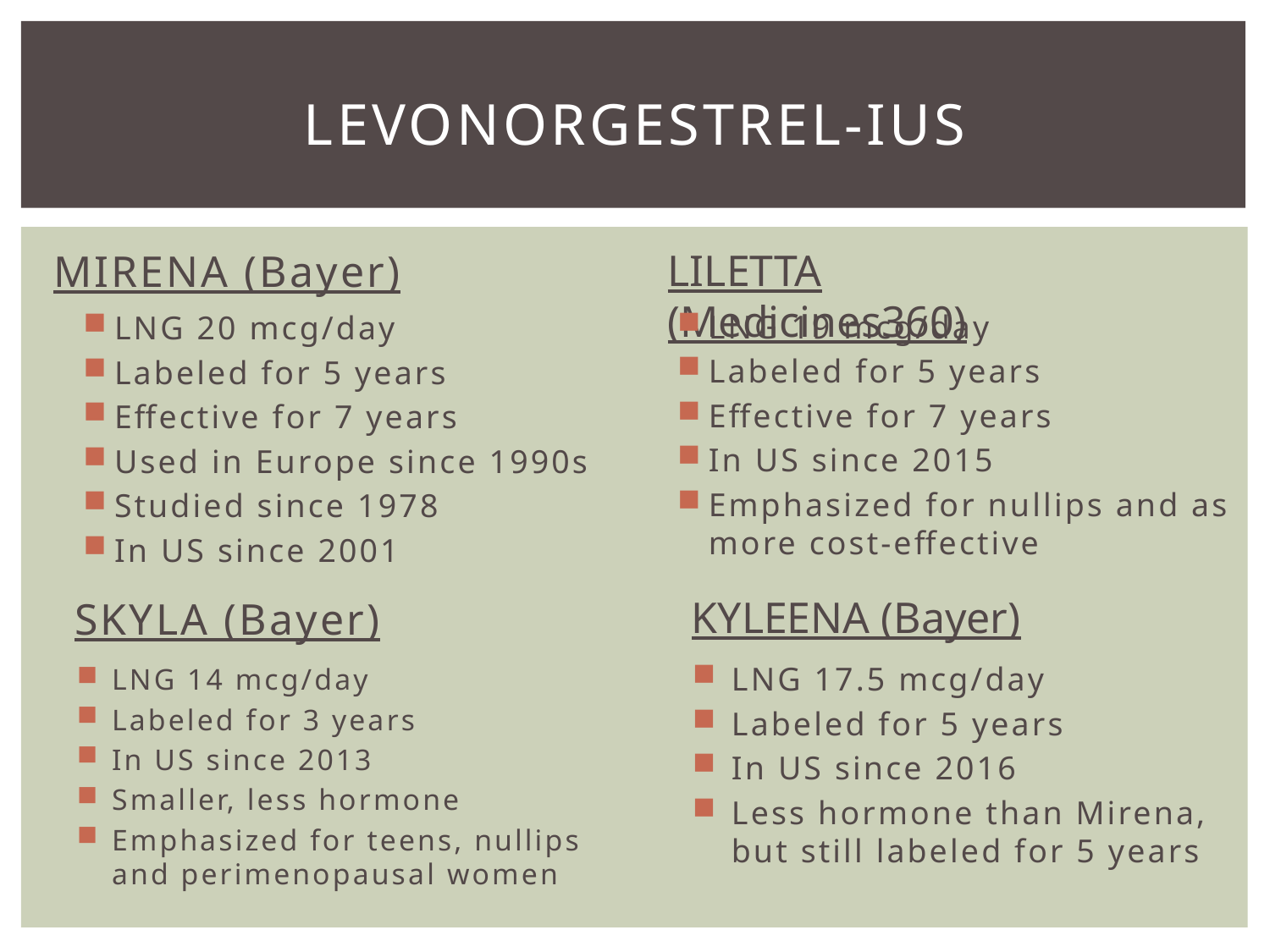

# LevoNorGestrel-IUS
MIRENA (Bayer)
LILETTA (Medicines360)
LNG 19 mcg/day
Labeled for 5 years
Effective for 7 years
In US since 2015
Emphasized for nullips and as more cost-effective
LNG 20 mcg/day
Labeled for 5 years
Effective for 7 years
Used in Europe since 1990s
Studied since 1978
In US since 2001
SKYLA (Bayer)
KYLEENA (Bayer)
LNG 17.5 mcg/day
Labeled for 5 years
In US since 2016
Less hormone than Mirena, but still labeled for 5 years
LNG 14 mcg/day
Labeled for 3 years
In US since 2013
Smaller, less hormone
Emphasized for teens, nullips and perimenopausal women

## Slide 10
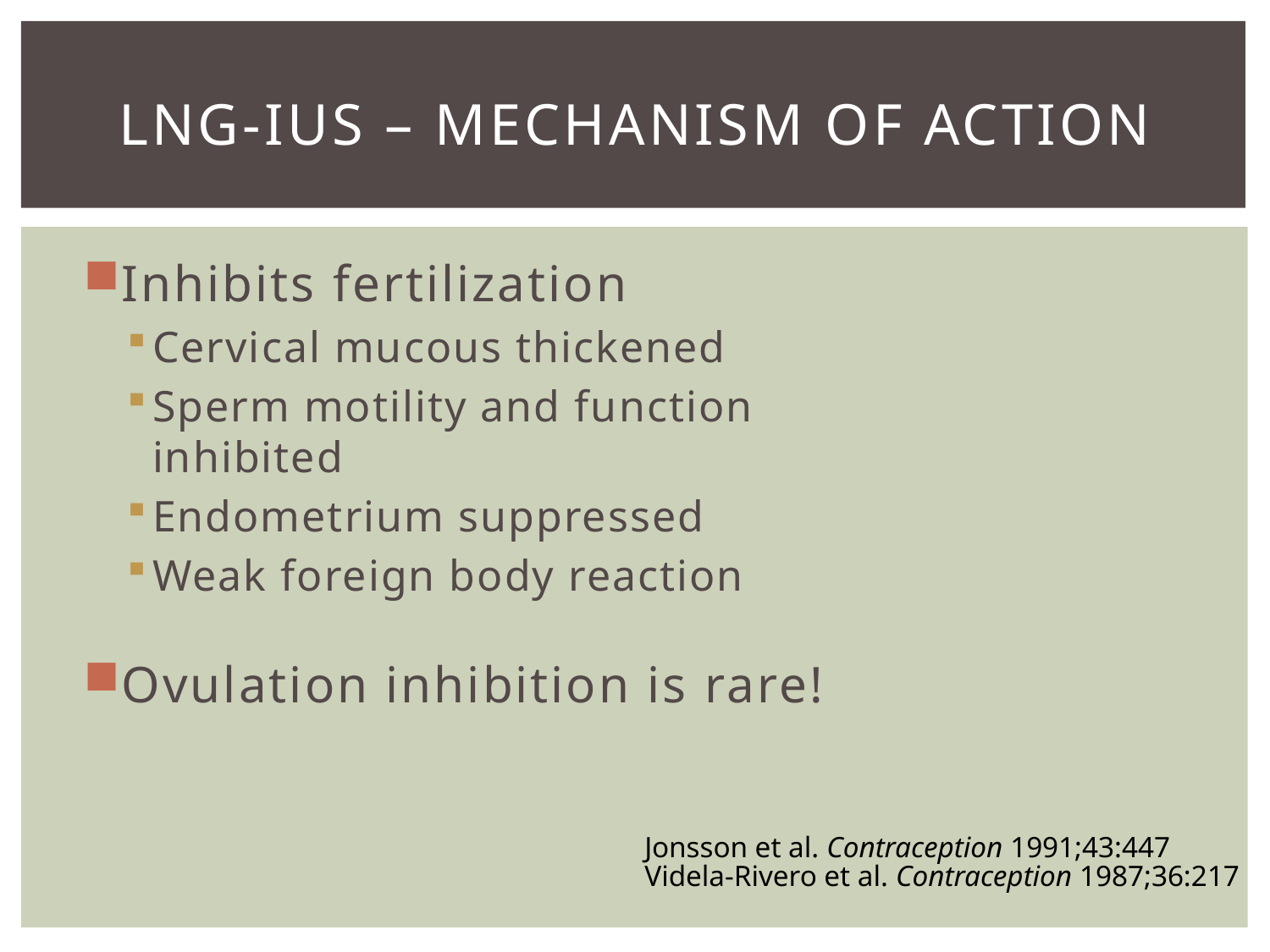

# LNG-IUS – Mechanism of action
Inhibits fertilization
Cervical mucous thickened
Sperm motility and function inhibited
Endometrium suppressed
Weak foreign body reaction
Ovulation inhibition is rare!
Jonsson et al. Contraception 1991;43:447
Videla-Rivero et al. Contraception 1987;36:217

## Slide 11
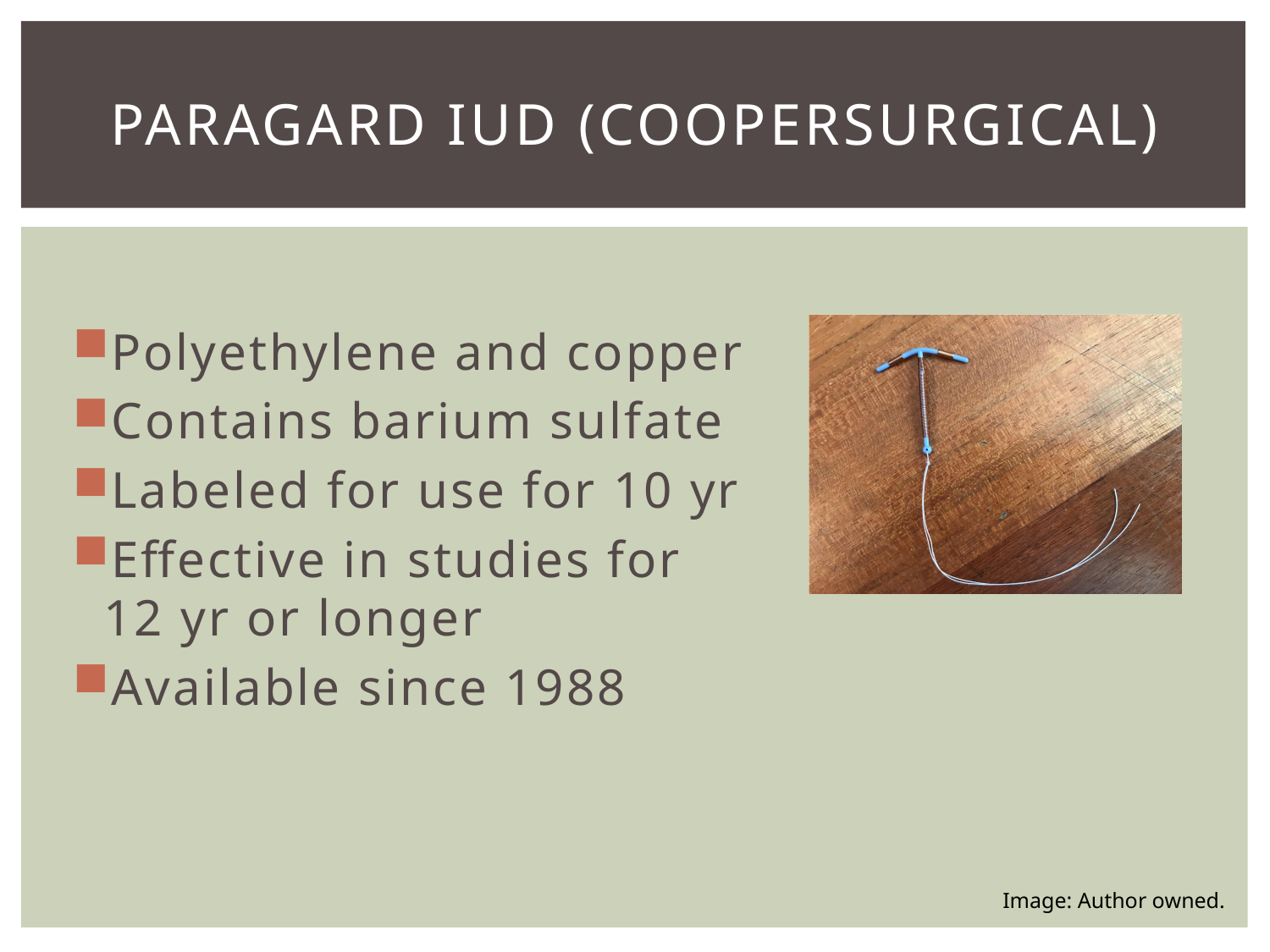

# Paragard IUD (Coopersurgical)
Polyethylene and copper
Contains barium sulfate
Labeled for use for 10 yr
Effective in studies for 12 yr or longer
Available since 1988
Image: Author owned.

## Slide 12
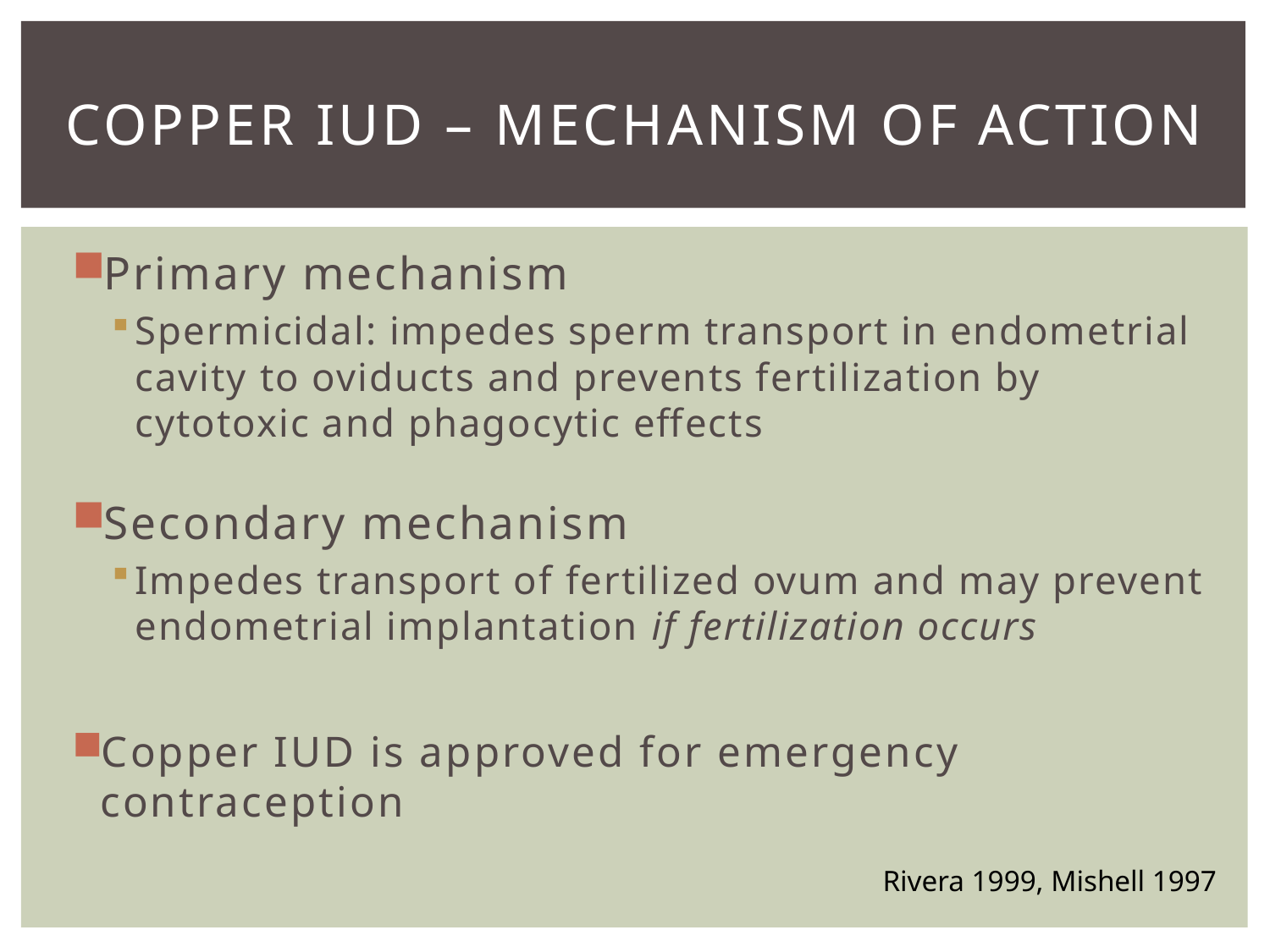

# Copper IUD – Mechanism of action
Primary mechanism
Spermicidal: impedes sperm transport in endometrial cavity to oviducts and prevents fertilization by cytotoxic and phagocytic effects
Secondary mechanism
Impedes transport of fertilized ovum and may prevent endometrial implantation if fertilization occurs
Copper IUD is approved for emergency contraception
Rivera 1999, Mishell 1997

## Slide 13
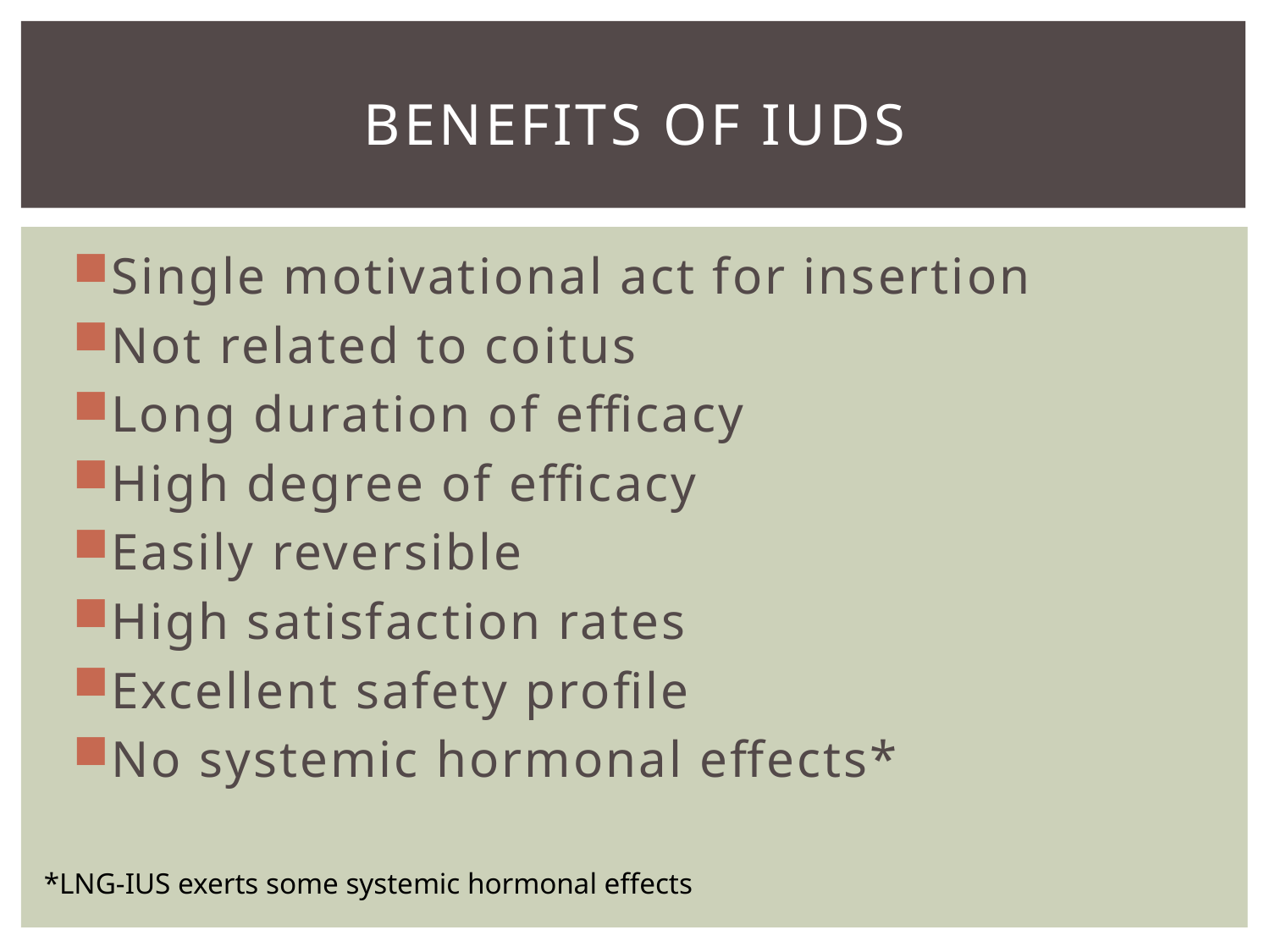

# Benefits of IUDs
Single motivational act for insertion
Not related to coitus
Long duration of efficacy
High degree of efficacy
Easily reversible
High satisfaction rates
Excellent safety profile
No systemic hormonal effects*
*LNG-IUS exerts some systemic hormonal effects

## Slide 14
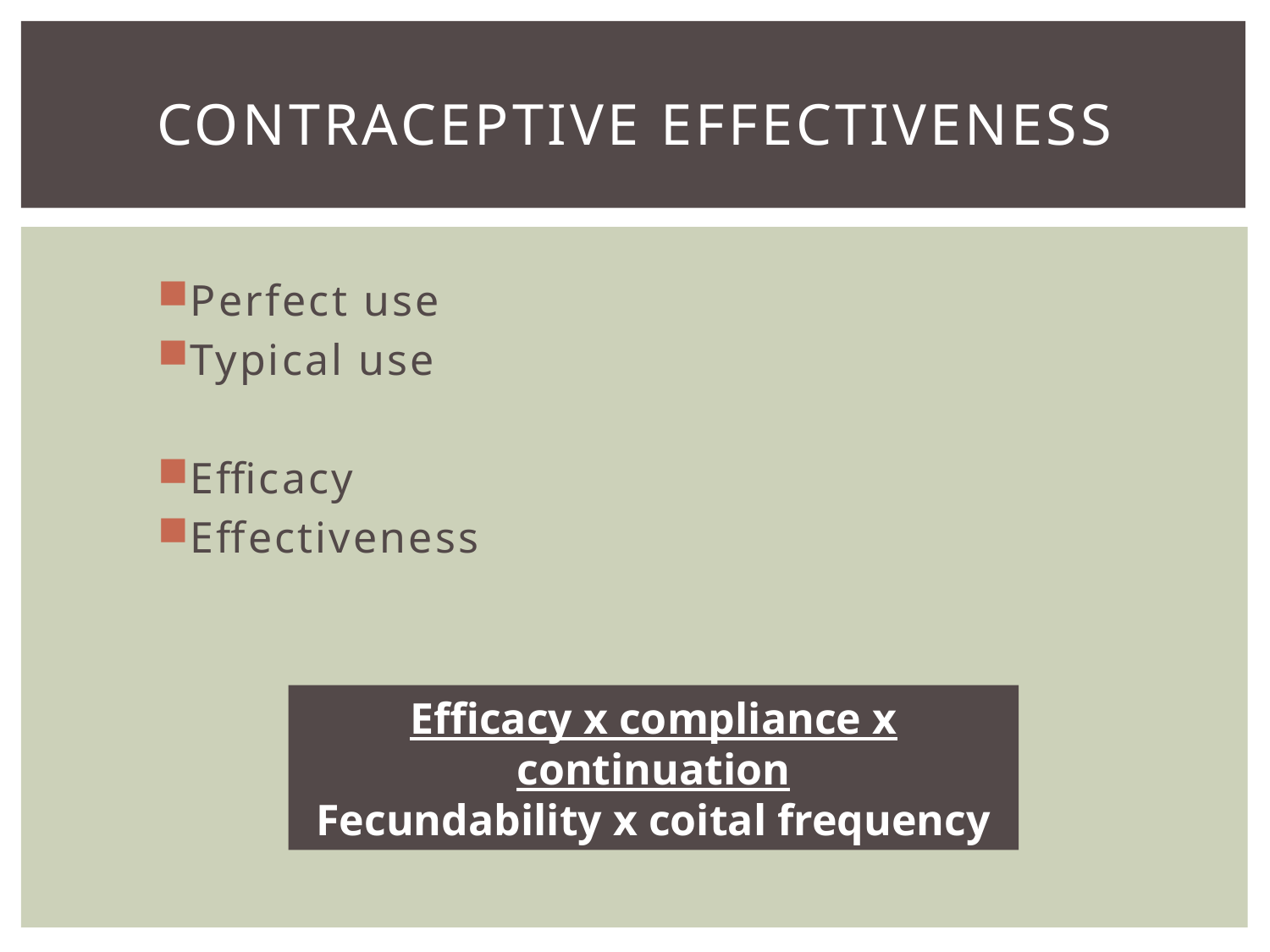

# Contraceptive Effectiveness
Perfect use
Typical use
Efficacy
Effectiveness
Efficacy x compliance x continuation
Fecundability x coital frequency

## Slide 15
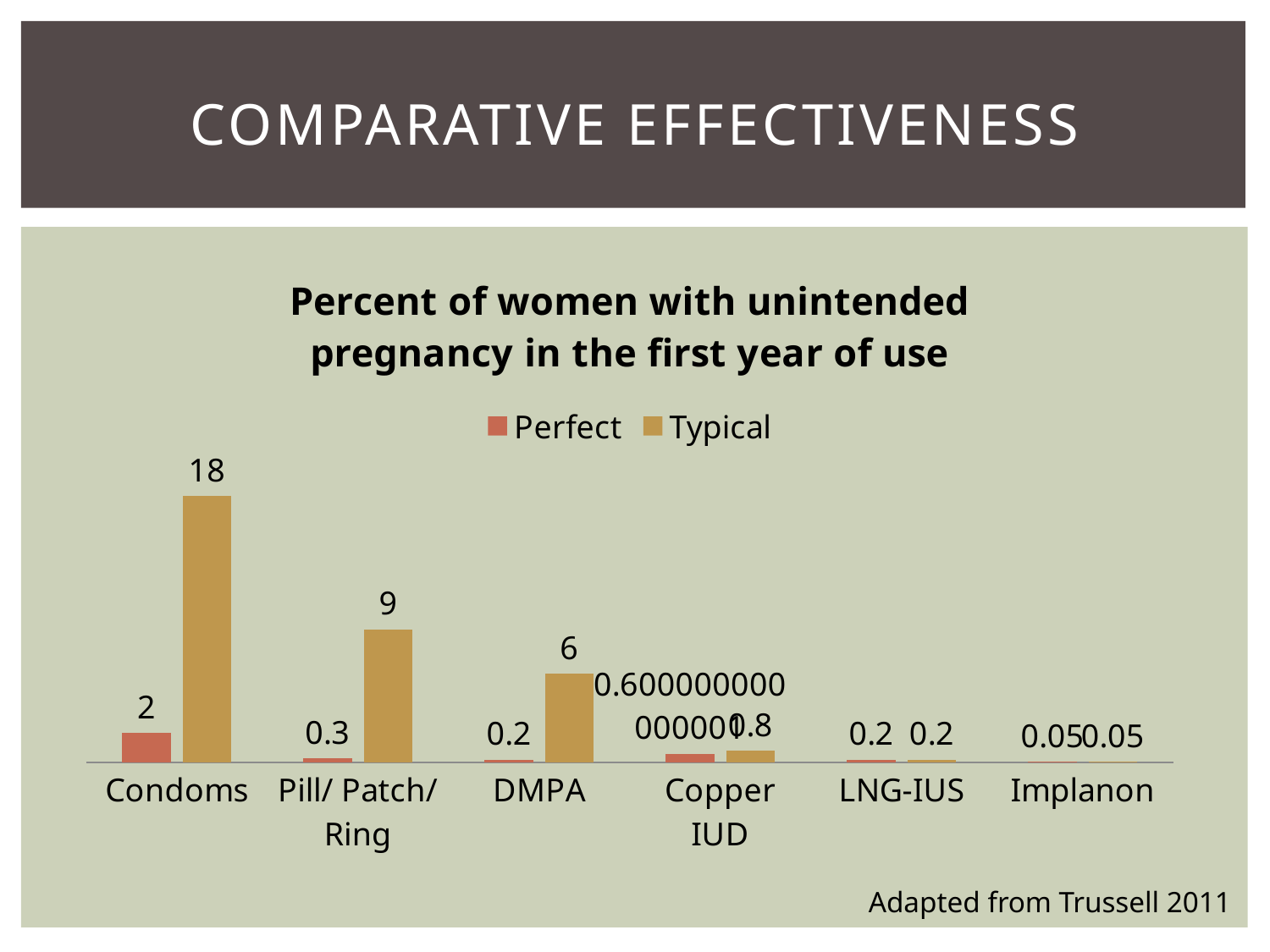

# Comparative Effectiveness
### Chart: Percent of women with unintended pregnancy in the first year of use
| Category | Perfect | Typical |
|---|---|---|
| Condoms | 2.0 | 18.0 |
| Pill/ Patch/ Ring | 0.3 | 9.0 |
| DMPA | 0.2 | 6.0 |
| Copper IUD | 0.600000000000001 | 0.8 |
| LNG-IUS | 0.2 | 0.2 |
| Implanon | 0.05 | 0.05 |Adapted from Trussell 2011

## Slide 16
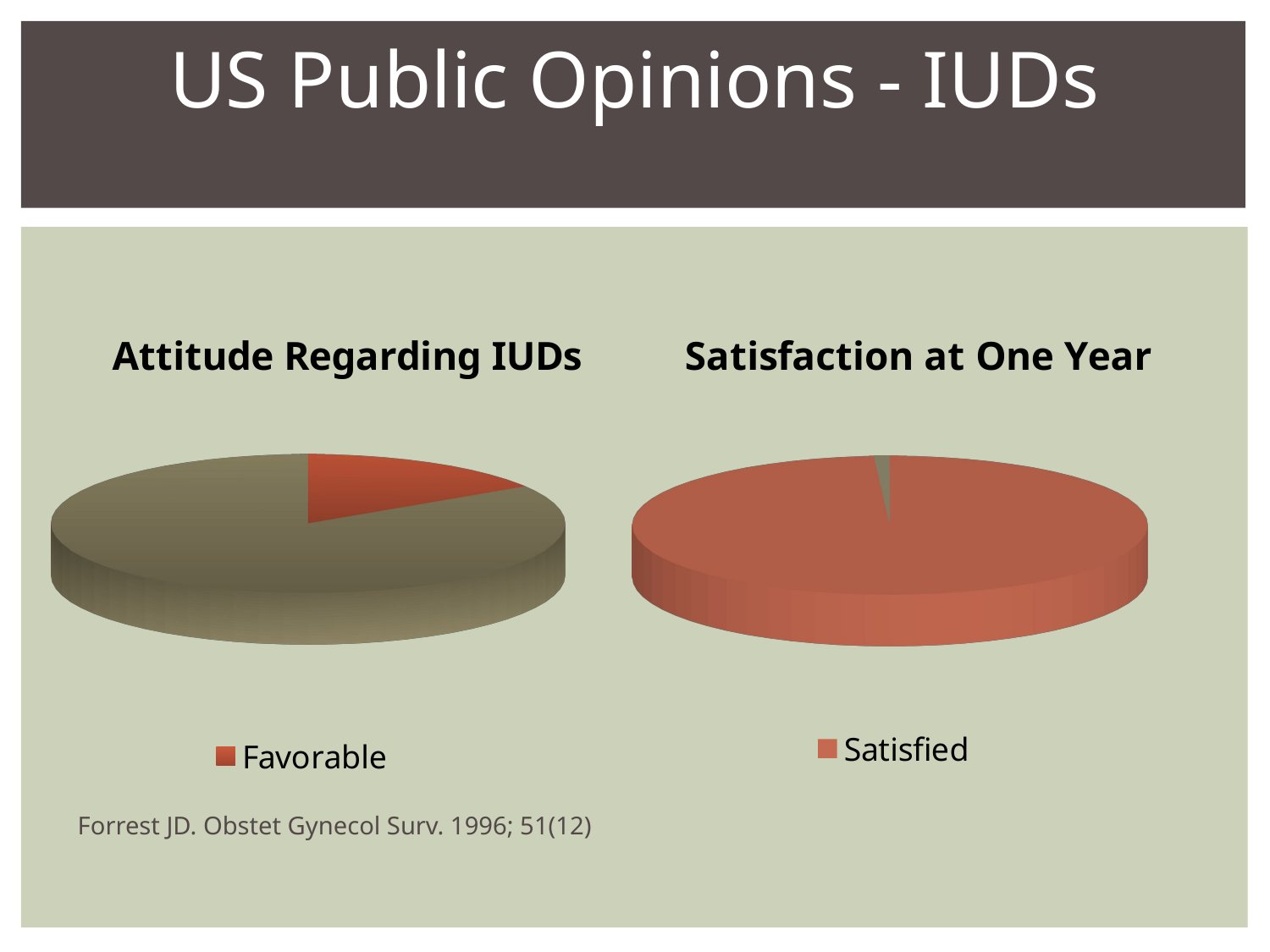

US Public Opinions - IUDs
[unsupported chart]
[unsupported chart]
Forrest JD. Obstet Gynecol Surv. 1996; 51(12)

## Slide 17
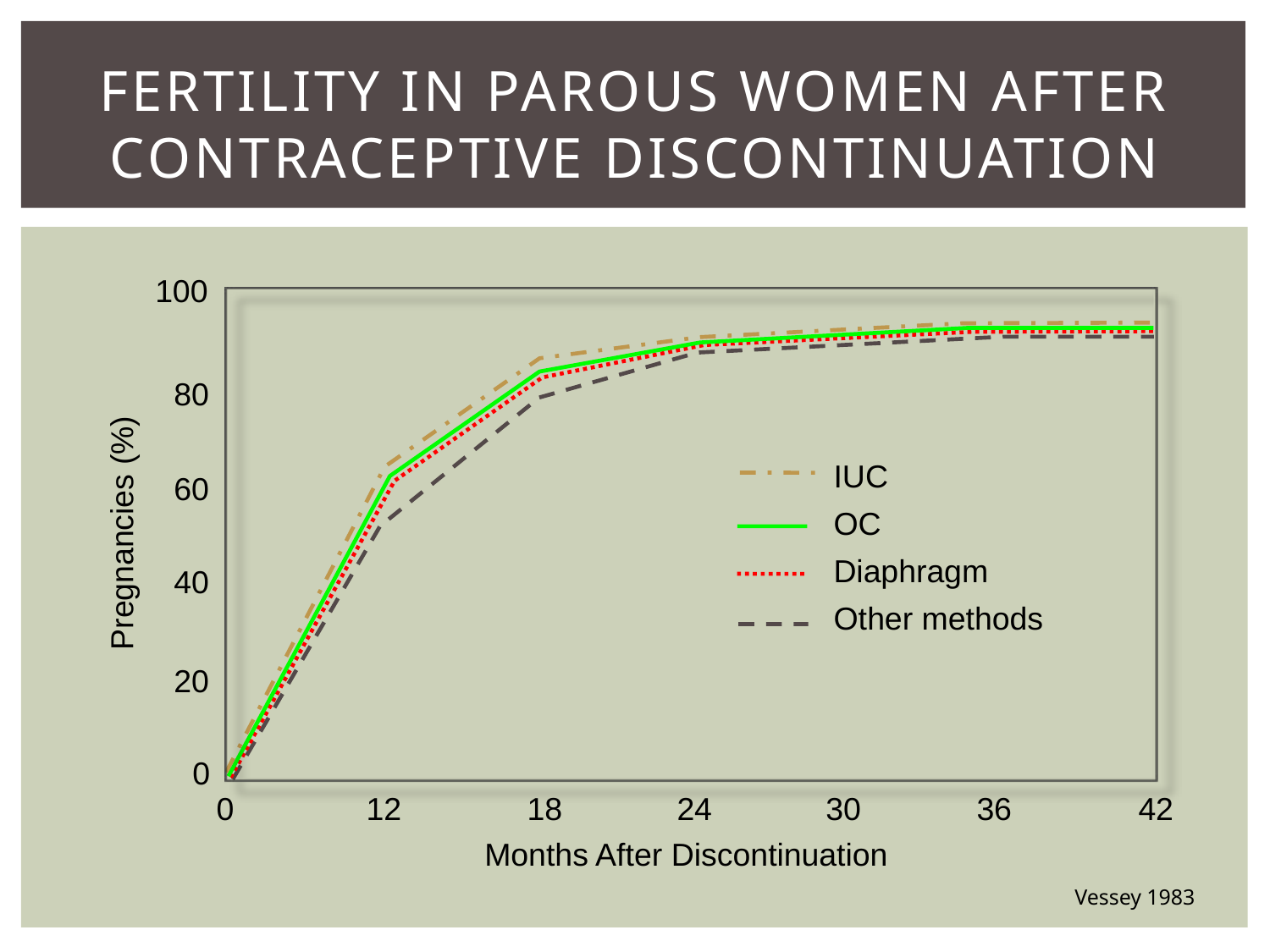

# Fertility in Parous Women after Contraceptive Discontinuation
100
80
IUC
OC
Diaphragm
Other methods
60
40
20
0
12
18
24
30
36
42
0
Months After Discontinuation
Pregnancies (%)
Vessey 1983

## Slide 18
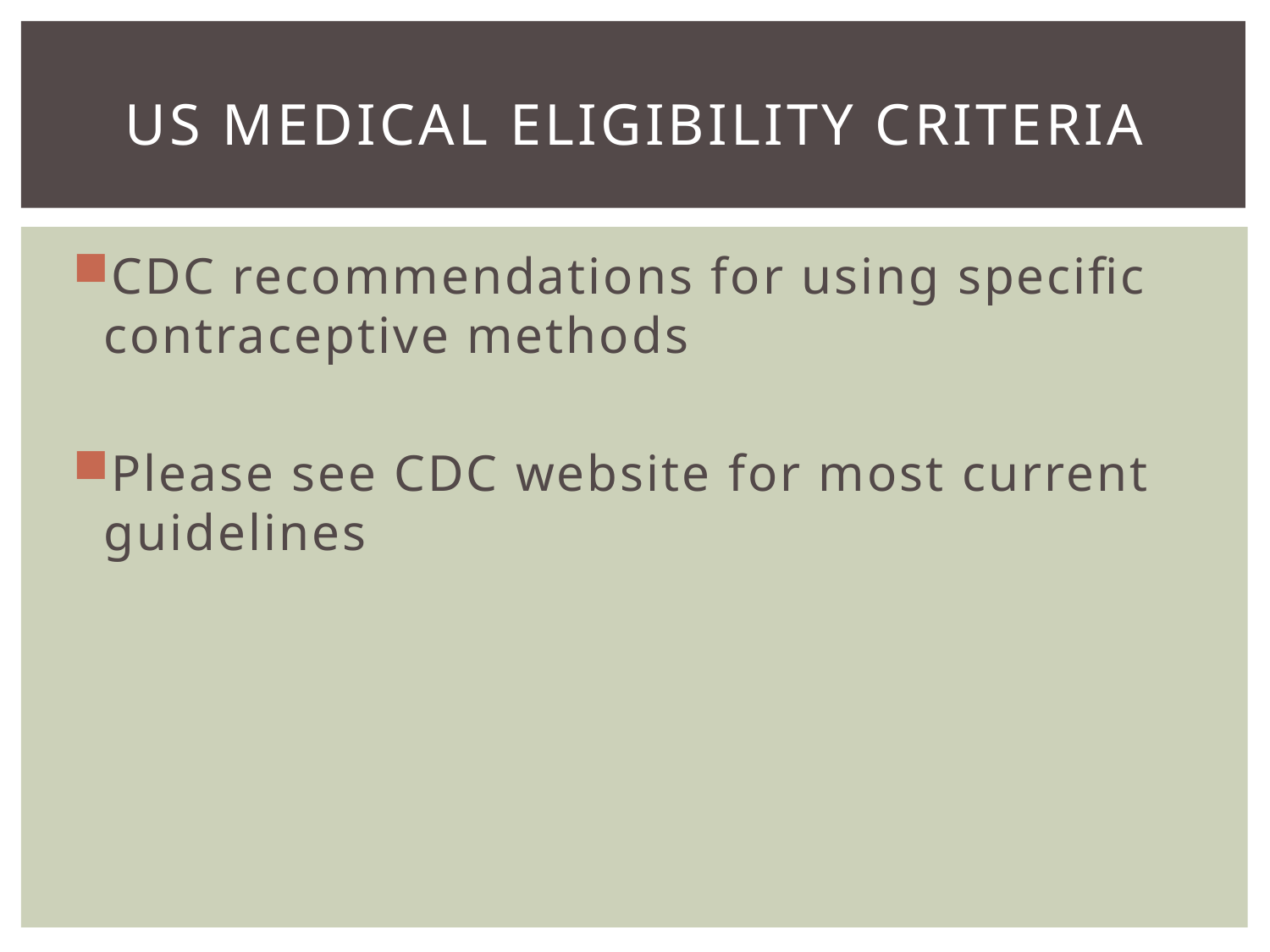

# US Medical Eligibility Criteria
CDC recommendations for using specific contraceptive methods
Please see CDC website for most current guidelines

## Slide 19
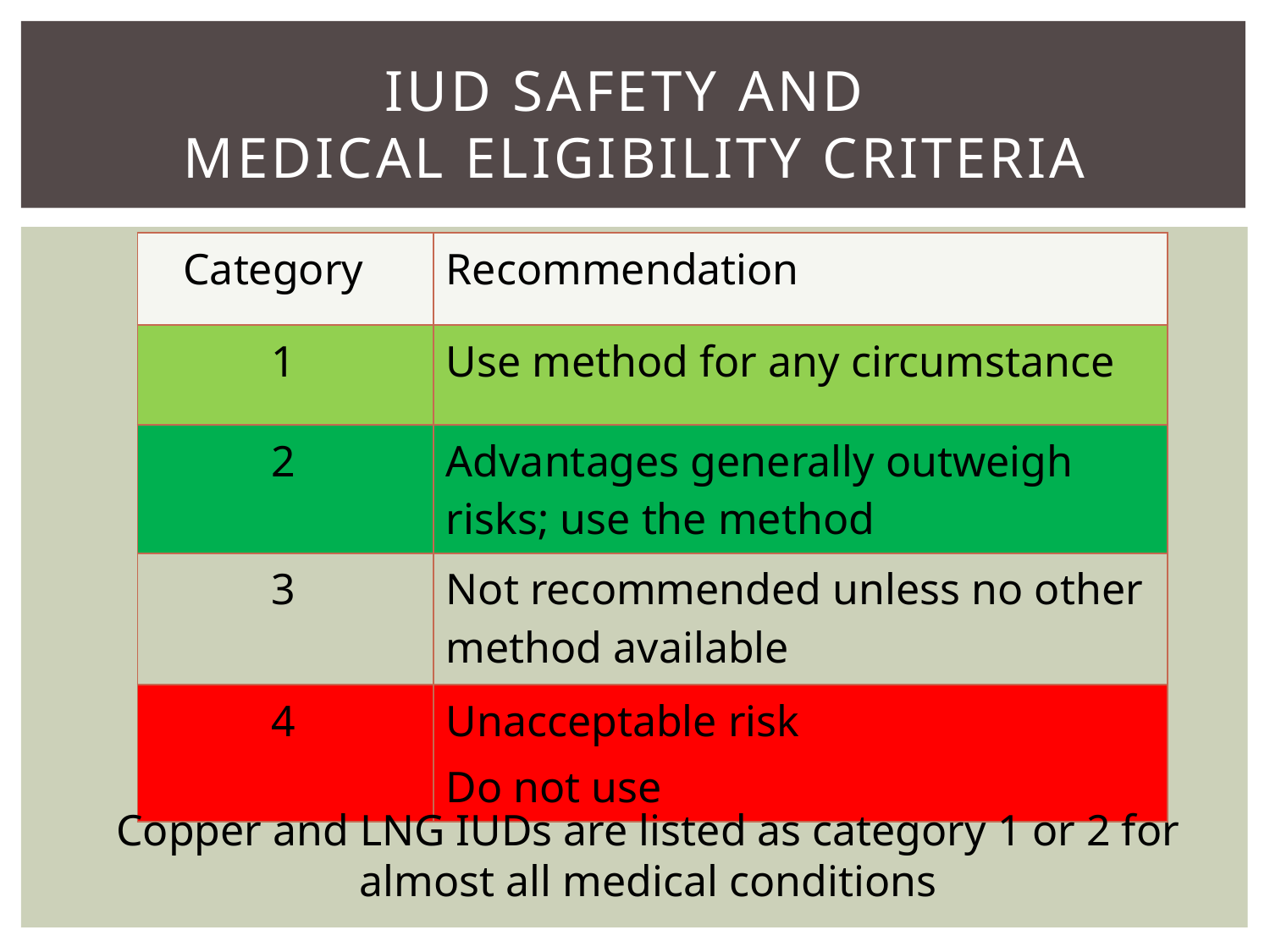

# IUD Safety and Medical Eligibility Criteria
| Category | Recommendation |
| --- | --- |
| 1 | Use method for any circumstance |
| 2 | Advantages generally outweigh risks; use the method |
| 3 | Not recommended unless no other method available |
| 4 | Unacceptable risk Do not use |
Copper and LNG IUDs are listed as category 1 or 2 for almost all medical conditions

## Slide 20
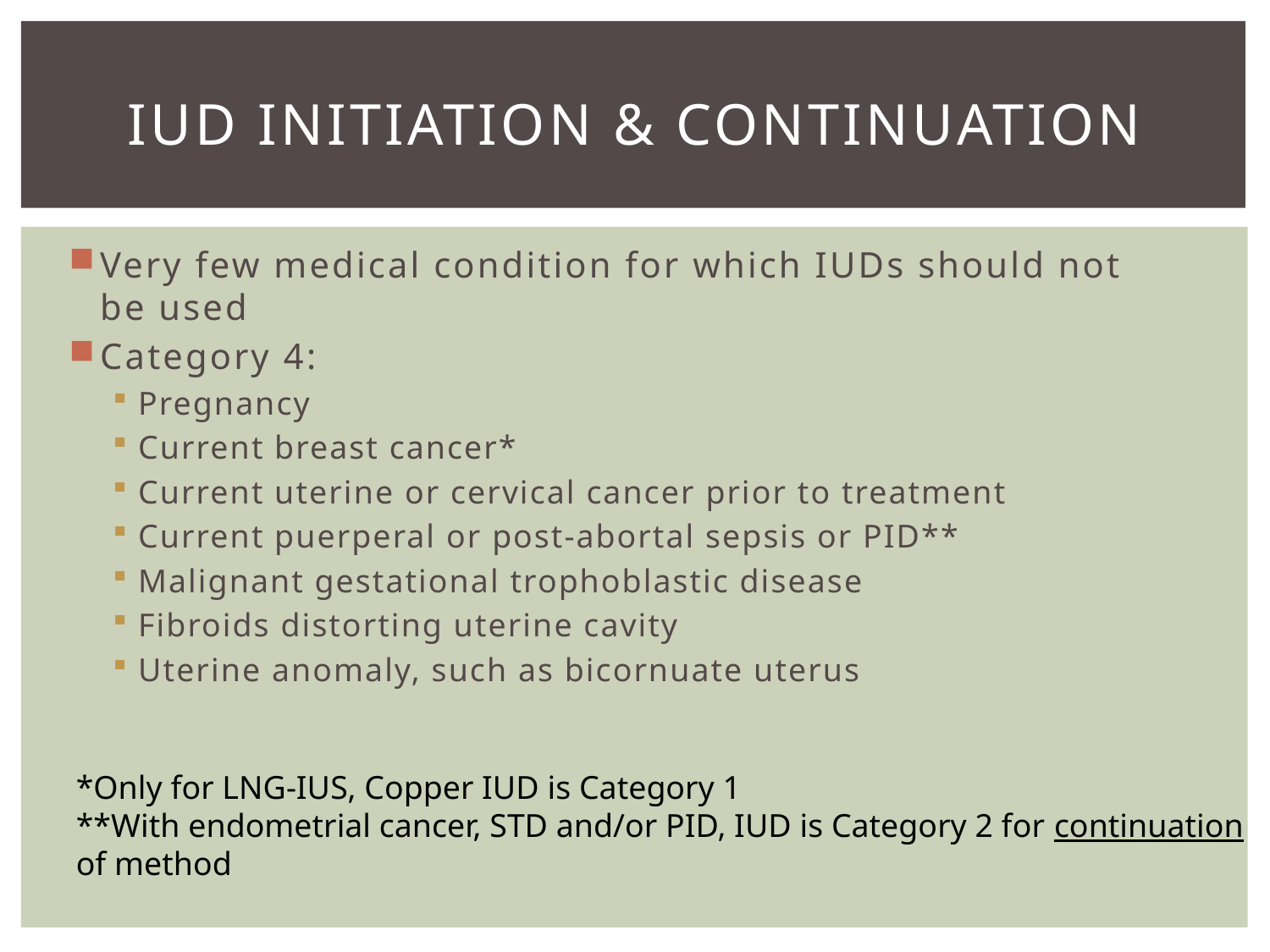

# IUD Initiation & Continuation
Very few medical condition for which IUDs should not be used
Category 4:
Pregnancy
Current breast cancer*
Current uterine or cervical cancer prior to treatment
Current puerperal or post-abortal sepsis or PID**
Malignant gestational trophoblastic disease
Fibroids distorting uterine cavity
Uterine anomaly, such as bicornuate uterus
*Only for LNG-IUS, Copper IUD is Category 1
**With endometrial cancer, STD and/or PID, IUD is Category 2 for continuation of method

## Slide 21
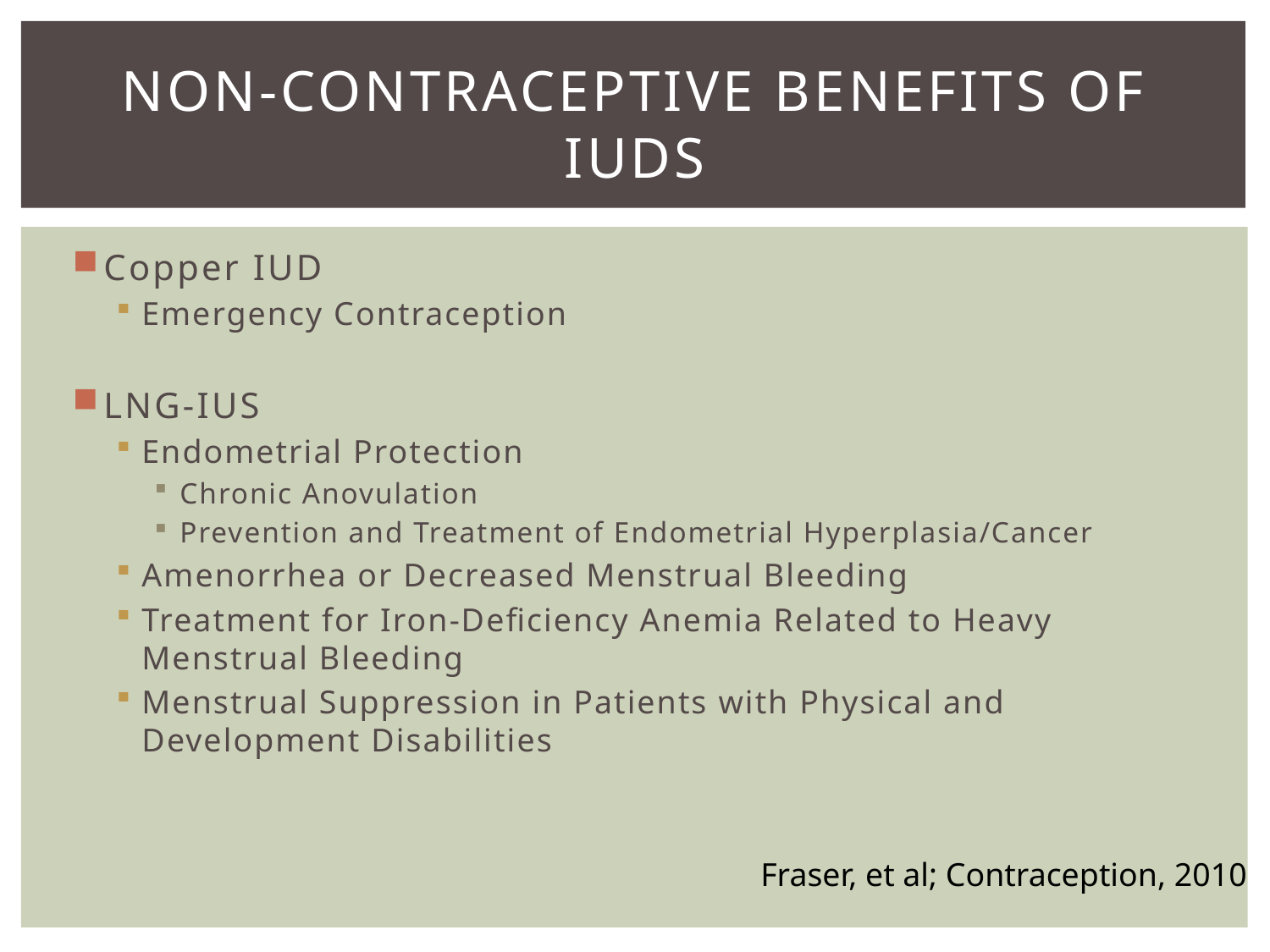

# Non-Contraceptive Benefits of IUDs
Copper IUD
Emergency Contraception
LNG-IUS
Endometrial Protection
Chronic Anovulation
Prevention and Treatment of Endometrial Hyperplasia/Cancer
Amenorrhea or Decreased Menstrual Bleeding
Treatment for Iron-Deficiency Anemia Related to Heavy Menstrual Bleeding
Menstrual Suppression in Patients with Physical and Development Disabilities
Fraser, et al; Contraception, 2010

## Slide 22
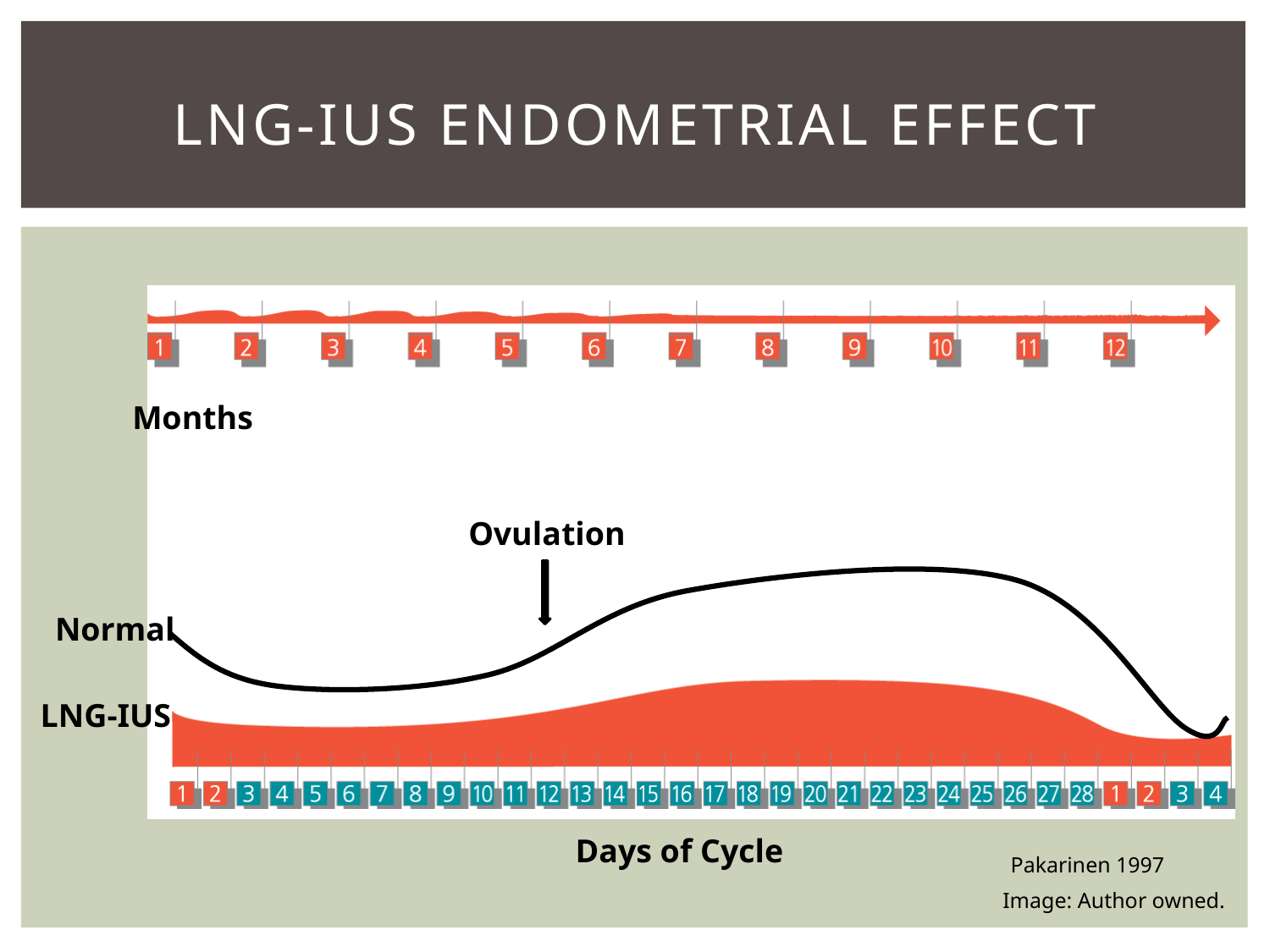

# LNG-IUS Endometrial Effect
Months
Ovulation
Normal
LNG-IUS
Days of Cycle
Pakarinen 1997
Image: Author owned.

## Slide 23
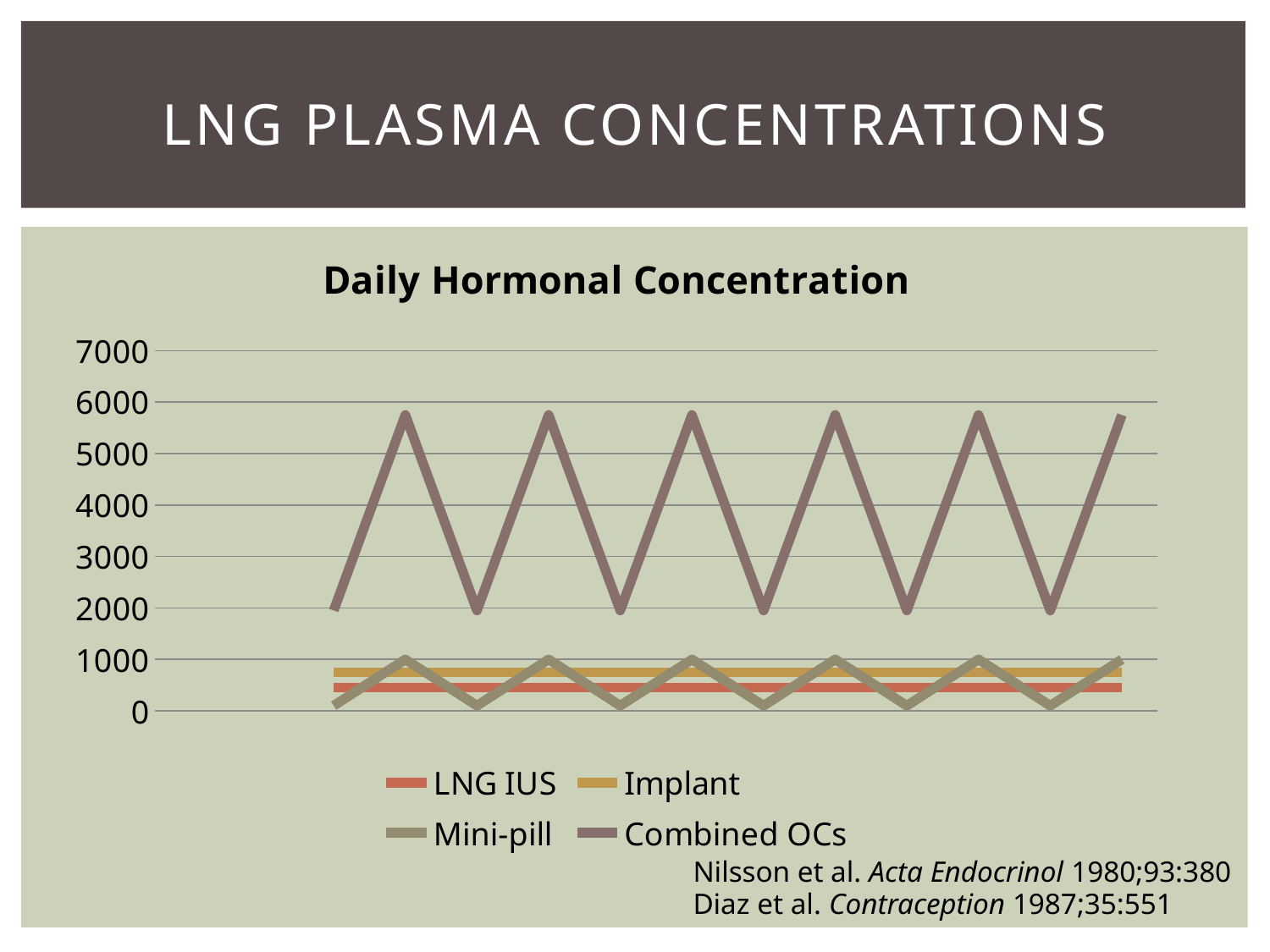

# LNG Plasma Concentrations
### Chart: Daily Hormonal Concentration
| Category | LNG IUS | Implant | Mini-pill | Combined OCs |
|---|---|---|---|---|
| 1.0 | None | None | None | None |
| 2.0 | None | None | None | None |
| 3.0 | 450.0 | 750.0 | 100.0 | 1950.0 |
| 4.0 | 450.0 | 750.0 | 1000.0 | 5750.0 |
| 5.0 | 450.0 | 750.0 | 100.0 | 1950.0 |
| 6.0 | 450.0 | 750.0 | 1000.0 | 5750.0 |
| 7.0 | 450.0 | 750.0 | 100.0 | 1950.0 |
| 8.0 | 450.0 | 750.0 | 1000.0 | 5750.0 |
| 9.0 | 450.0 | 750.0 | 100.0 | 1950.0 |
| 10.0 | 450.0 | 750.0 | 1000.0 | 5750.0 |
| 11.0 | 450.0 | 750.0 | 100.0 | 1950.0 |
| 12.0 | 450.0 | 750.0 | 1000.0 | 5750.0 |
| 13.0 | 450.0 | 750.0 | 100.0 | 1950.0 |
| 14.0 | 450.0 | 750.0 | 1000.0 | 5750.0 |Nilsson et al. Acta Endocrinol 1980;93:380
Diaz et al. Contraception 1987;35:551

## Slide 24
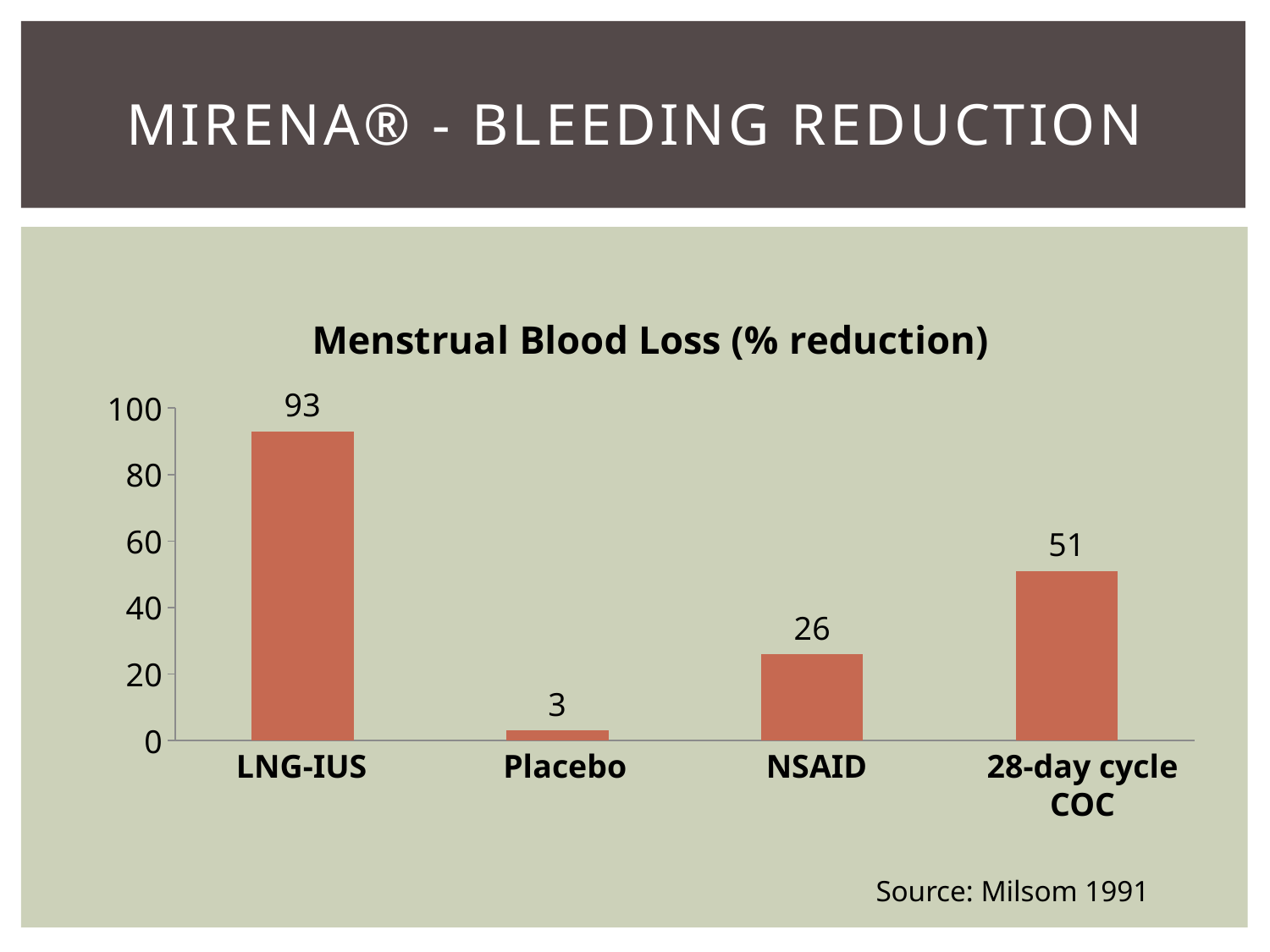

# Mirena® - Bleeding Reduction
### Chart:
| Category | Menstrual Blood Loss (% reduction) |
|---|---|
| LNG-IUS | 93.0 |
| Placebo | 3.0 |
| Prostaglandin-Synthetase Inhibitor | 26.0 |
| 28-day cycle COC | 51.0 |LNG-IUS
Placebo
NSAID
28-day cycle
COC
Source: Milsom 1991

## Slide 25
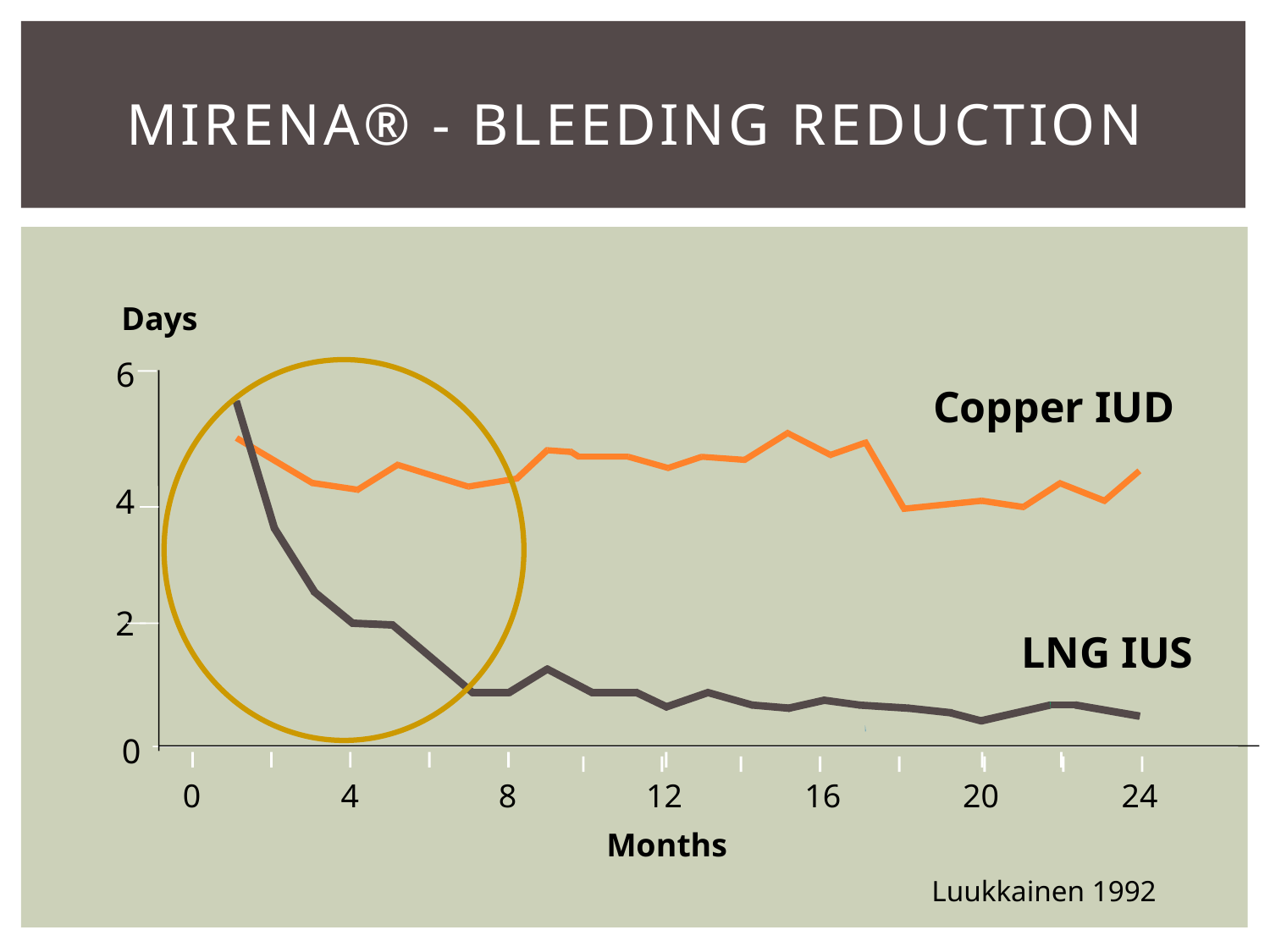

# Mirena® - Bleeding Reduction
Days
6
Copper IUD
4
2
LNG IUS
0
0
4
8
12
16
20
24
Months
Luukkainen 1992

## Slide 26
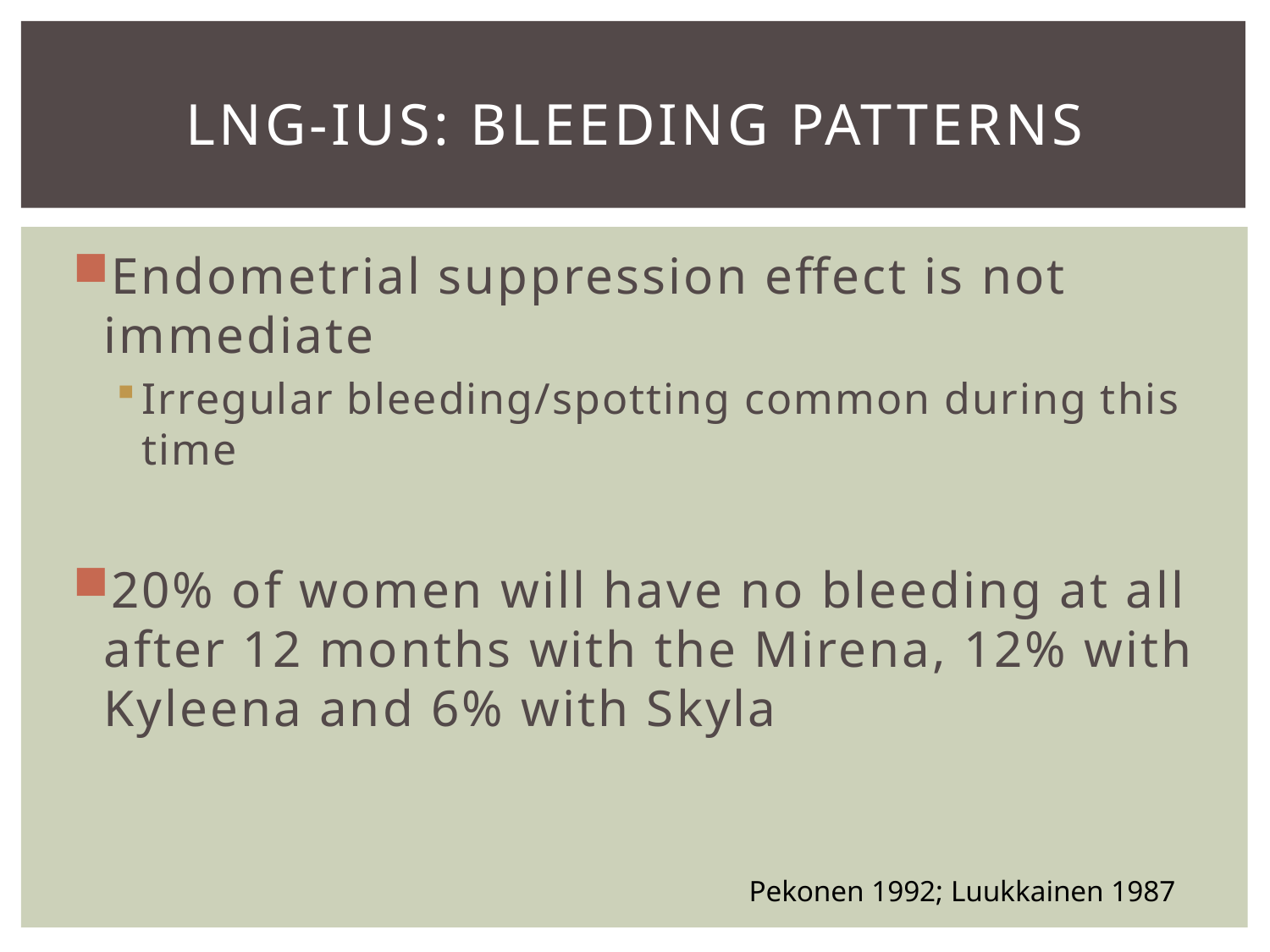

# LNG-IUS: Bleeding Patterns
Endometrial suppression effect is not immediate
Irregular bleeding/spotting common during this time
20% of women will have no bleeding at all after 12 months with the Mirena, 12% with Kyleena and 6% with Skyla
Pekonen 1992; Luukkainen 1987

## Slide 27
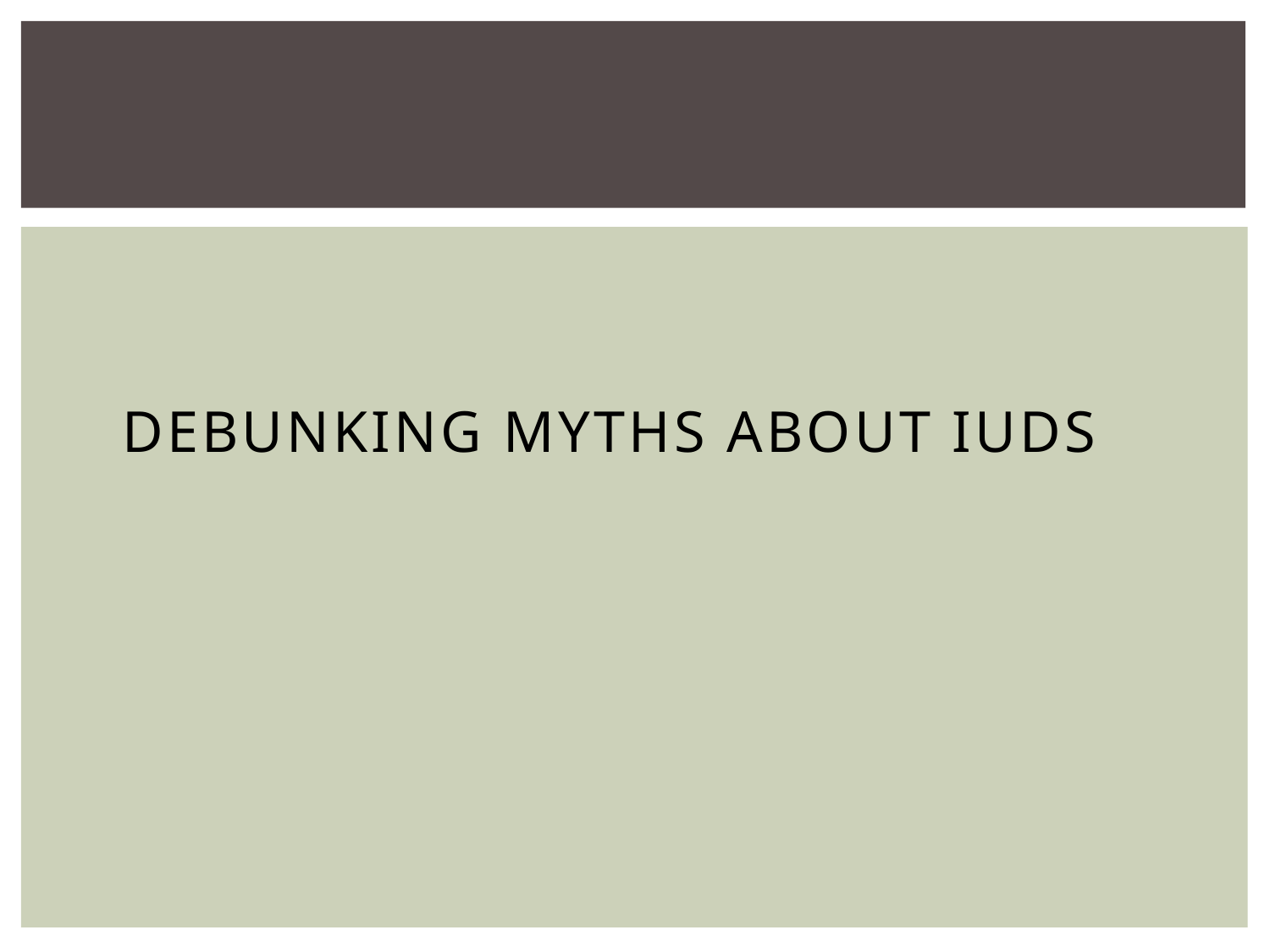

# Debunking Myths about IUDs

## Slide 28
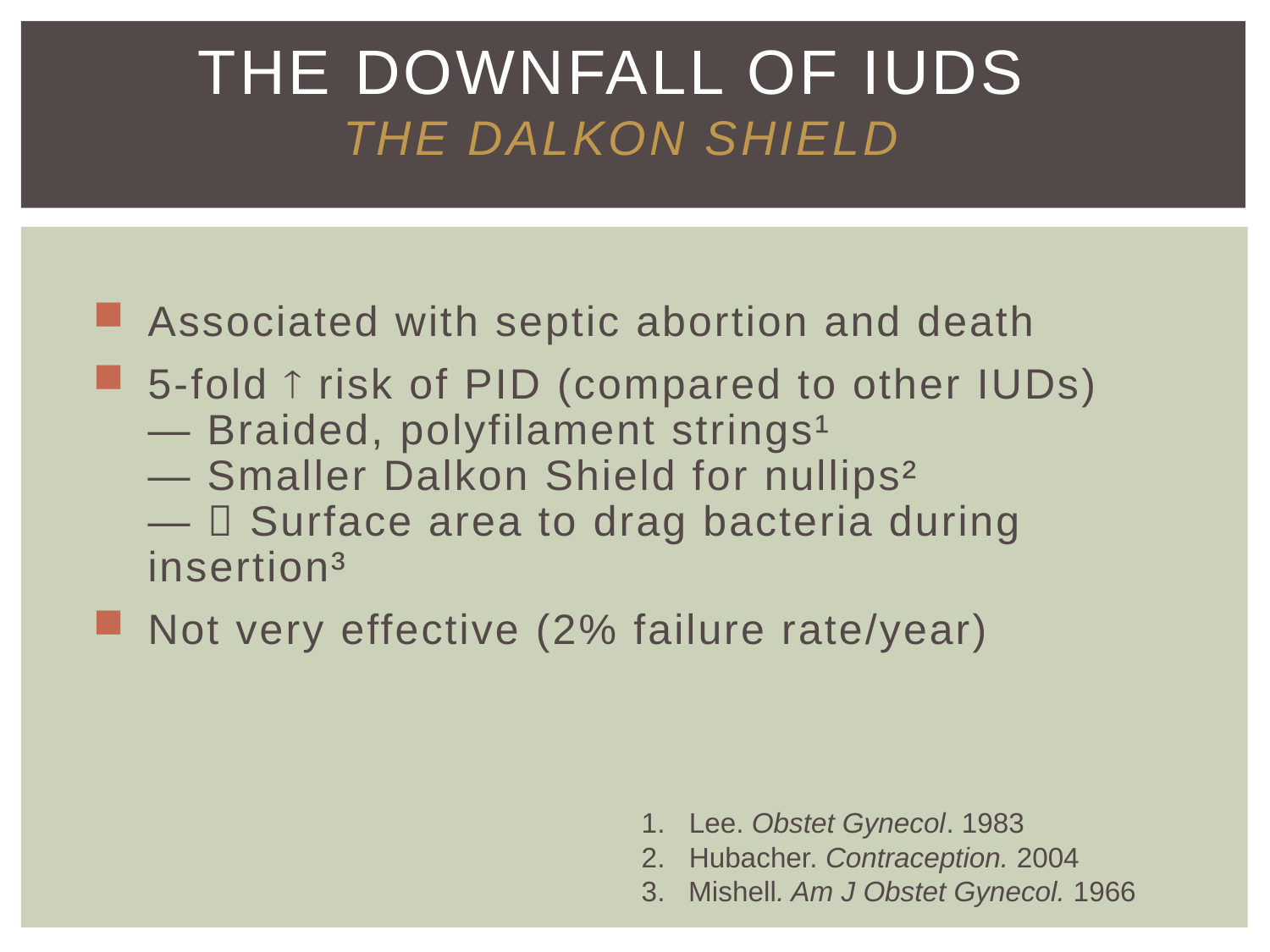

# The Downfall of IUDs The Dalkon Shield
Associated with septic abortion and death
5-fold  risk of PID (compared to other IUDs)
— Braided, polyfilament strings¹
— Smaller Dalkon Shield for nullips²
—  Surface area to drag bacteria during insertion³
Not very effective (2% failure rate/year)
Lee. Obstet Gynecol. 1983
Hubacher. Contraception. 2004
3. Mishell. Am J Obstet Gynecol. 1966

## Slide 29
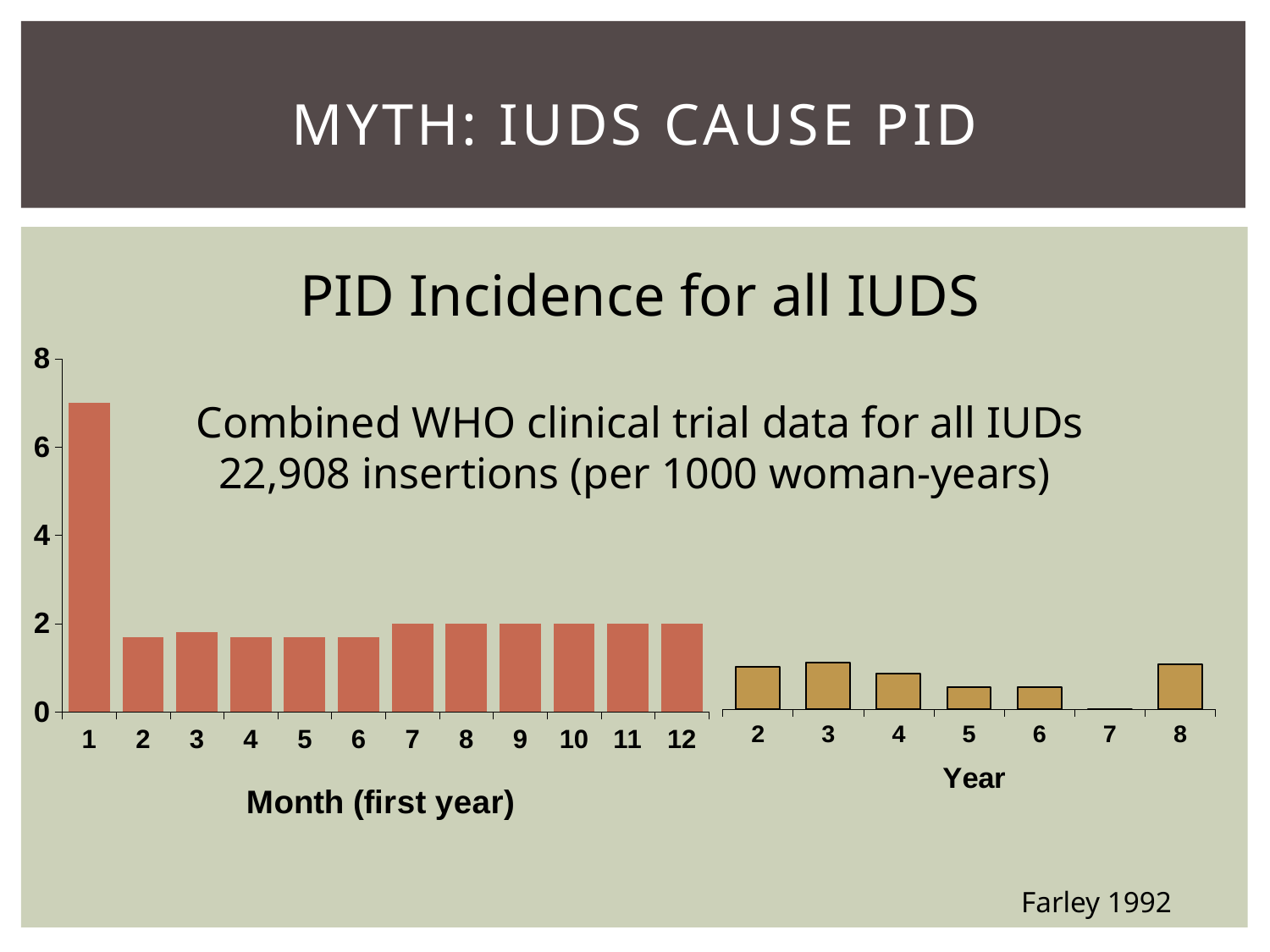

# Myth: IUDs Cause PID
PID Incidence for all IUDS
Combined WHO clinical trial data for all IUDs
22,908 insertions (per 1000 woman-years)
### Chart
| Category | |
|---|---|
| 1.0 | 7.0 |
| 2.0 | 1.7 |
| 3.0 | 1.8 |
| 4.0 | 1.7 |
| 5.0 | 1.7 |
| 6.0 | 1.7 |
| 7.0 | 2.0 |
| 8.0 | 2.0 |
| 9.0 | 2.0 |
| 10.0 | 2.0 |
| 11.0 | 2.0 |
| 12.0 | 2.0 |
### Chart
| Category | |
|---|---|
| 2.0 | 0.950000000000001 |
| 3.0 | 1.05 |
| 4.0 | 0.8 |
| 5.0 | 0.5 |
| 6.0 | 0.5 |
| 7.0 | 0.0 |
| 8.0 | 1.0 |Farley 1992

## Slide 30
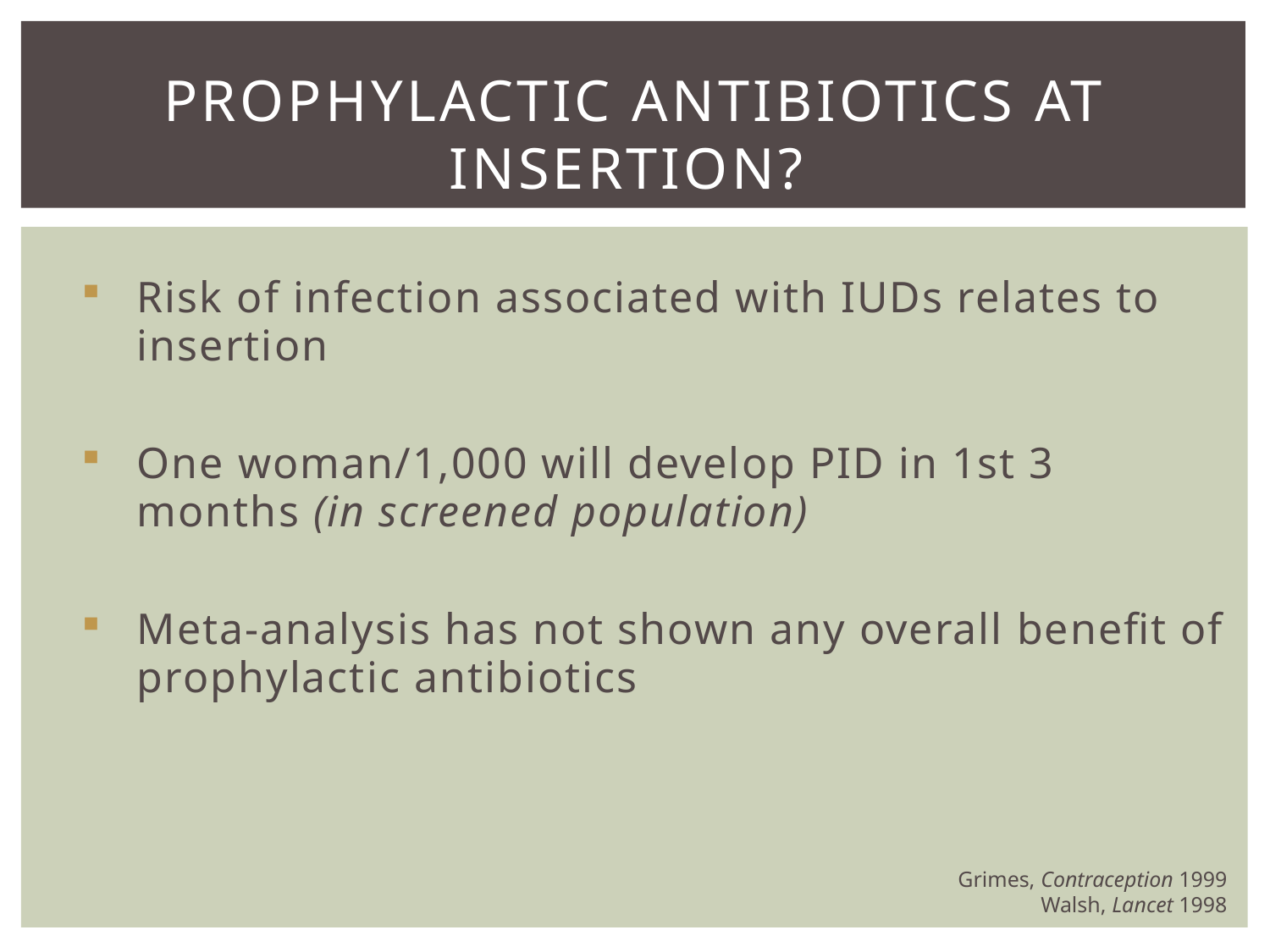

# Prophylactic Antibiotics at Insertion?
Risk of infection associated with IUDs relates to insertion
One woman/1,000 will develop PID in 1st 3 months (in screened population)
Meta-analysis has not shown any overall benefit of prophylactic antibiotics
Grimes, Contraception 1999
Walsh, Lancet 1998

## Slide 31
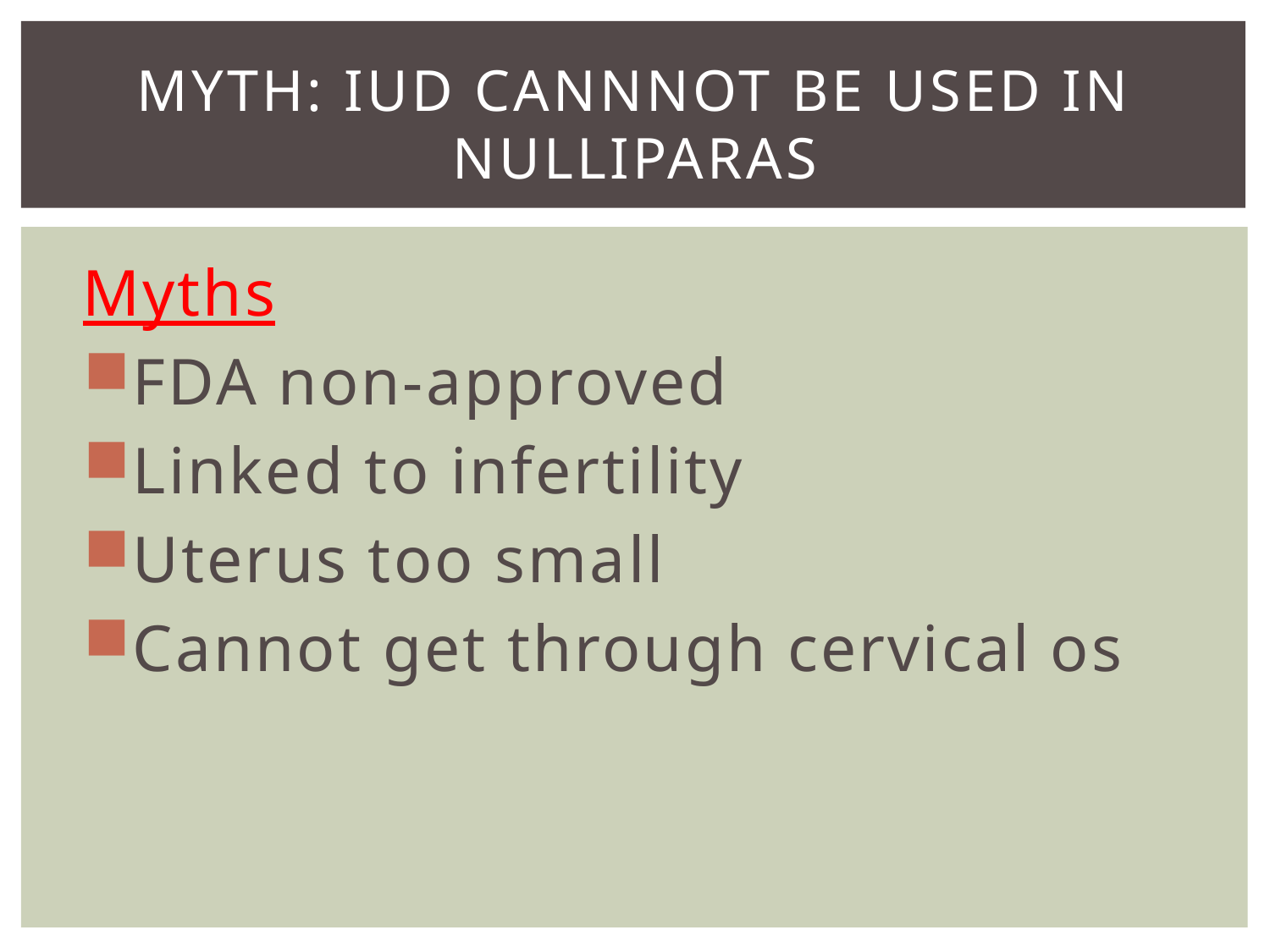

# Myth: iud cannnot be used in Nulliparas
Myths
FDA non-approved
Linked to infertility
Uterus too small
Cannot get through cervical os

## Slide 32
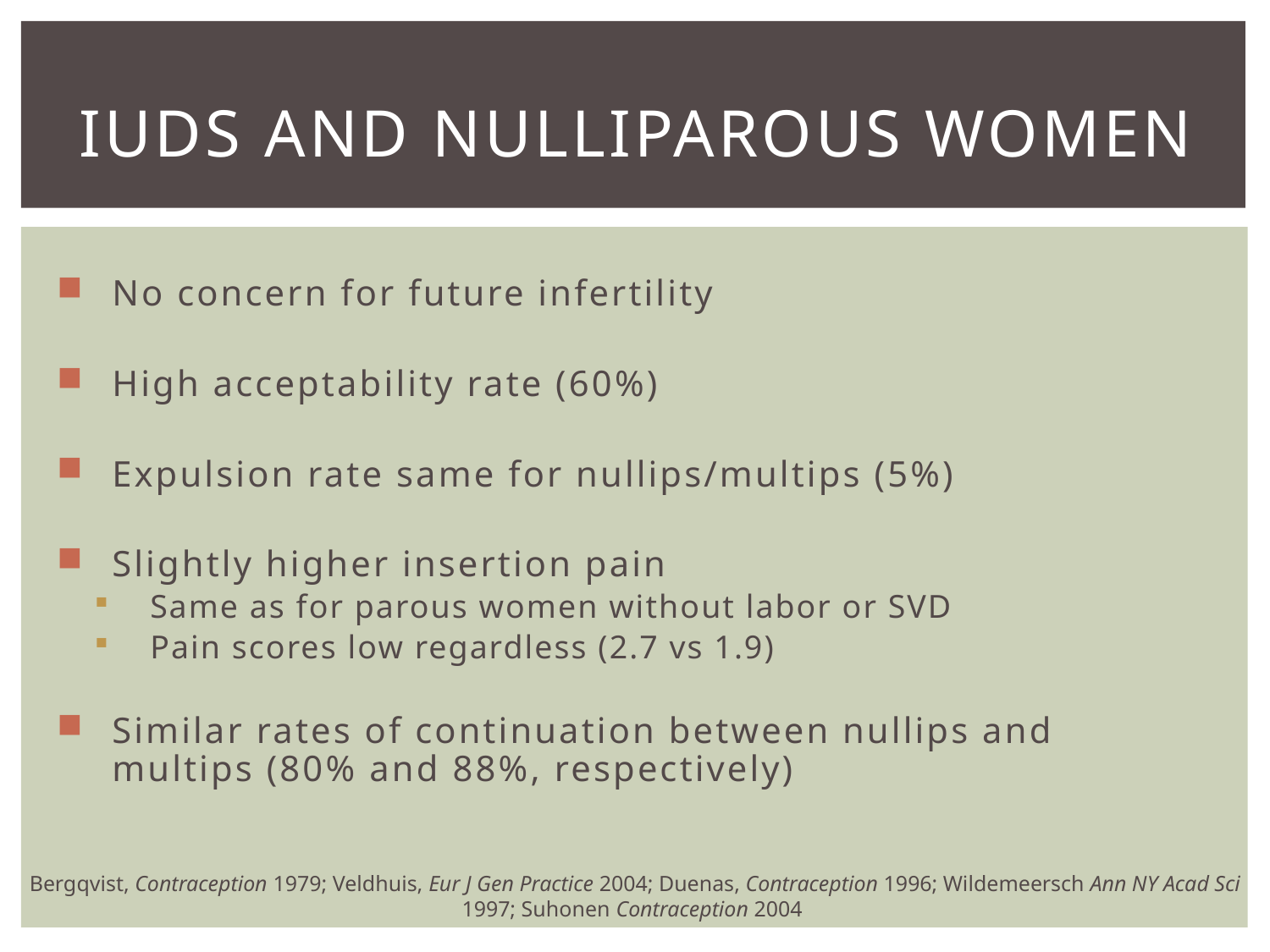

# IUDs and Nulliparous Women
No concern for future infertility
High acceptability rate (60%)
Expulsion rate same for nullips/multips (5%)
Slightly higher insertion pain
Same as for parous women without labor or SVD
Pain scores low regardless (2.7 vs 1.9)
Similar rates of continuation between nullips and multips (80% and 88%, respectively)
Bergqvist, Contraception 1979; Veldhuis, Eur J Gen Practice 2004; Duenas, Contraception 1996; Wildemeersch Ann NY Acad Sci 1997; Suhonen Contraception 2004

## Slide 33
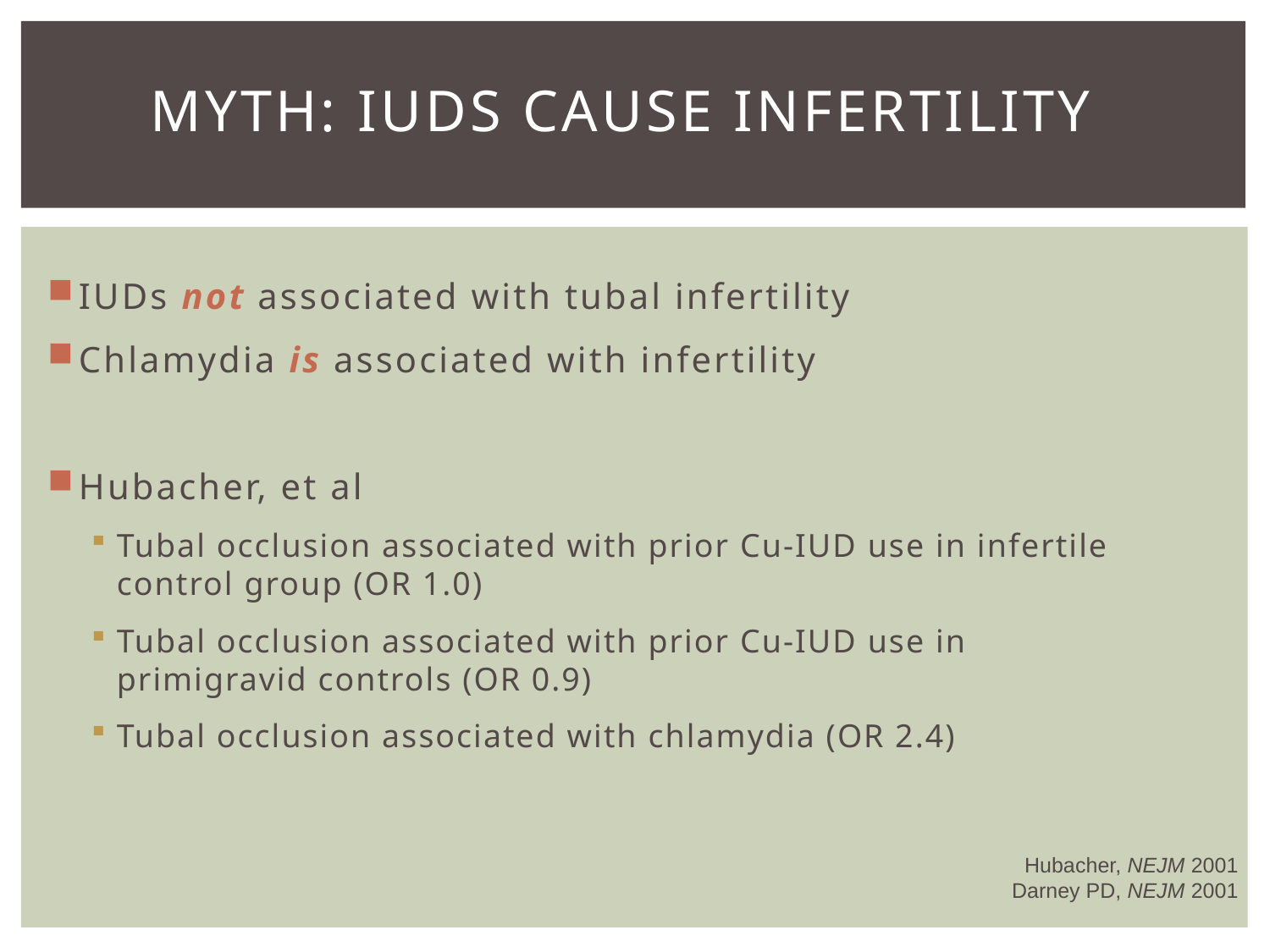

# Myth: IUDs cause Infertility
IUDs not associated with tubal infertility
Chlamydia is associated with infertility
Hubacher, et al
Tubal occlusion associated with prior Cu-IUD use in infertile control group (OR 1.0)
Tubal occlusion associated with prior Cu-IUD use in primigravid controls (OR 0.9)
Tubal occlusion associated with chlamydia (OR 2.4)
Hubacher, NEJM 2001
Darney PD, NEJM 2001

## Slide 34
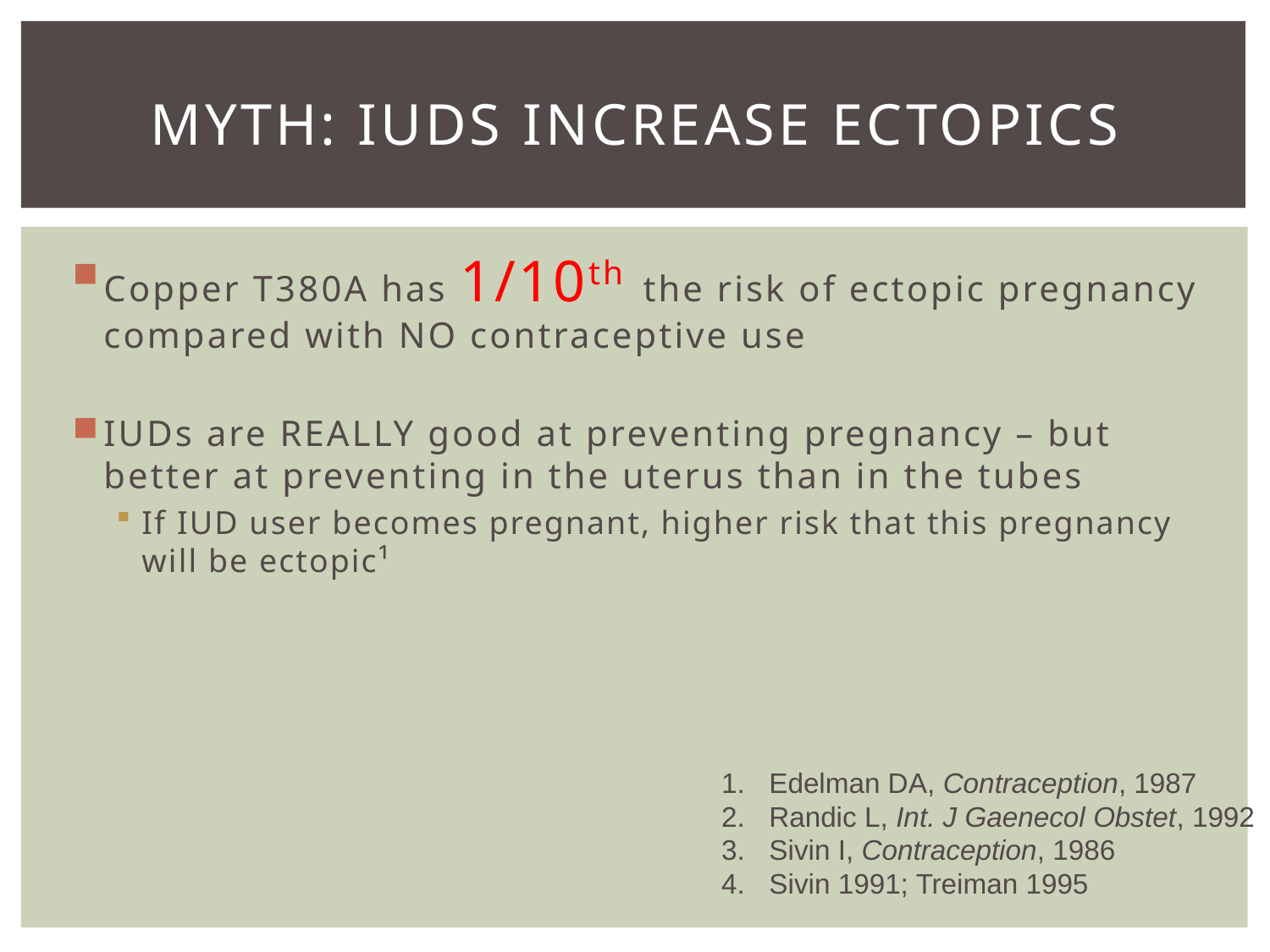

# Myth: IUDs Increase Ectopics
Copper T380A has 1/10th the risk of ectopic pregnancy compared with NO contraceptive use
IUDs are REALLY good at preventing pregnancy – but better at preventing in the uterus than in the tubes
If IUD user becomes pregnant, higher risk that this pregnancy will be ectopic¹
Edelman DA, Contraception, 1987
Randic L, Int. J Gaenecol Obstet, 1992
Sivin I, Contraception, 1986
Sivin 1991; Treiman 1995

## Slide 35
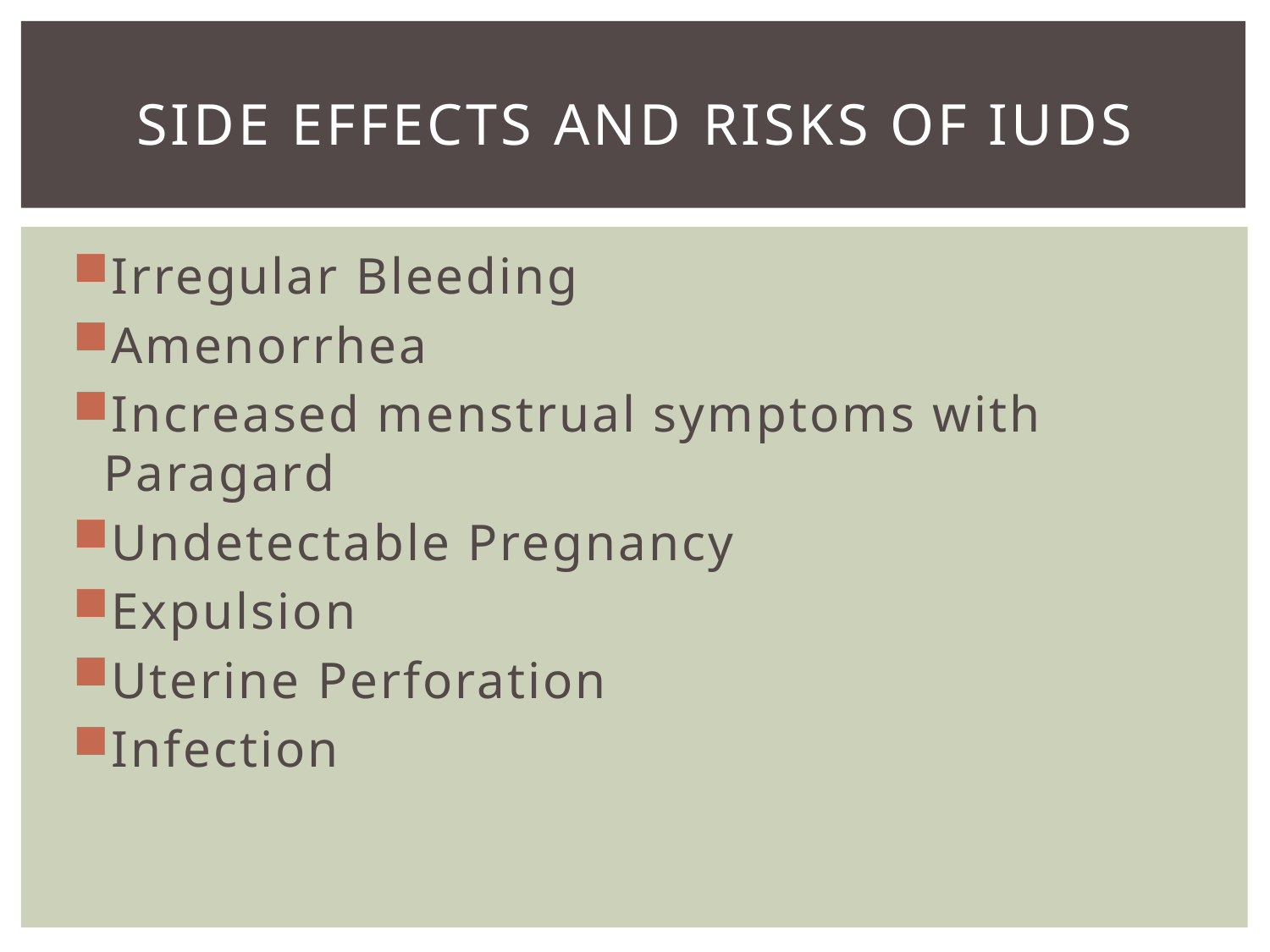

# side effects and risks of IUDs
Irregular Bleeding
Amenorrhea
Increased menstrual symptoms with Paragard
Undetectable Pregnancy
Expulsion
Uterine Perforation
Infection

## Slide 36
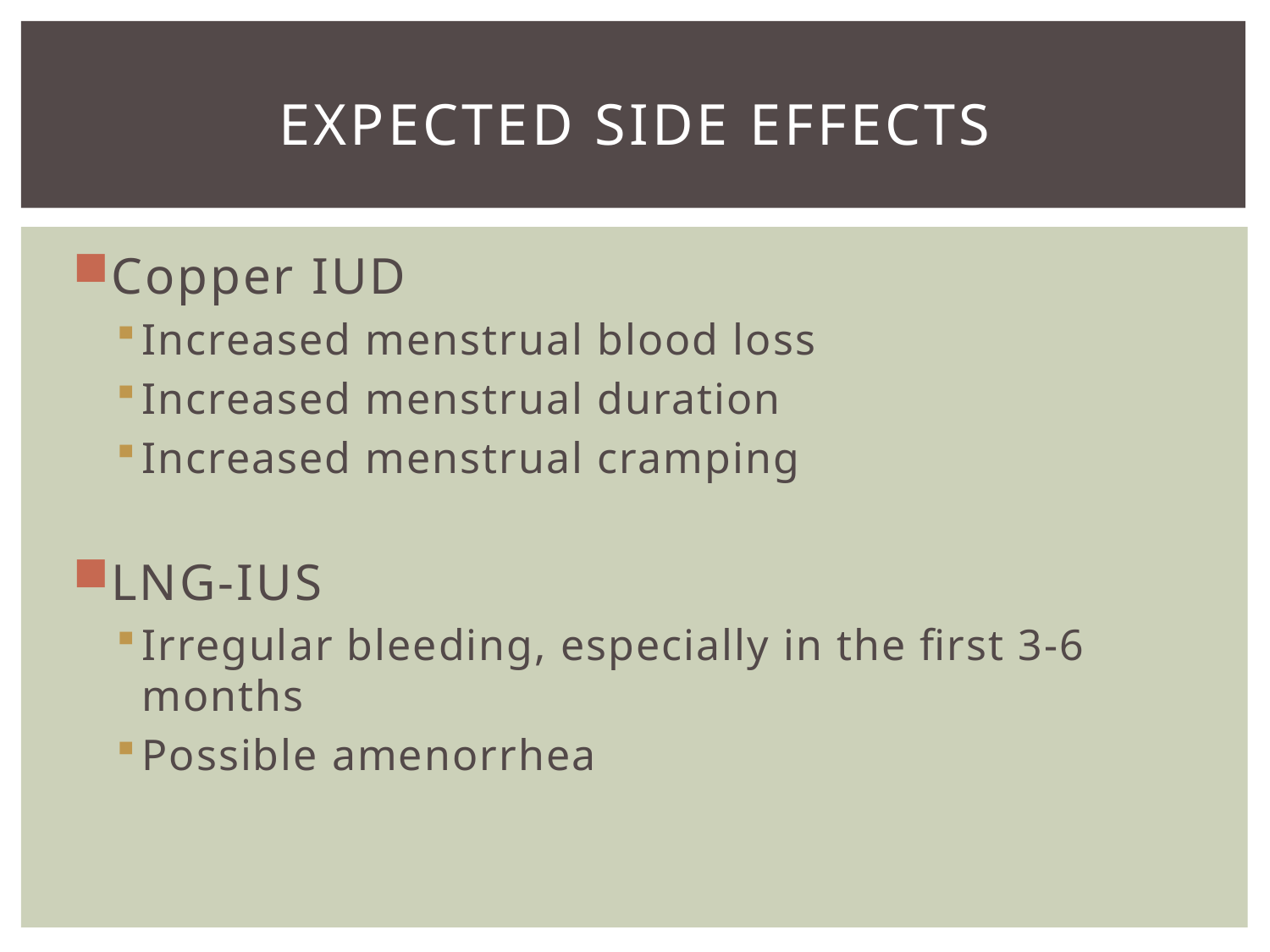

# Expected Side Effects
Copper IUD
Increased menstrual blood loss
Increased menstrual duration
Increased menstrual cramping
LNG-IUS
Irregular bleeding, especially in the first 3-6 months
Possible amenorrhea

## Slide 37
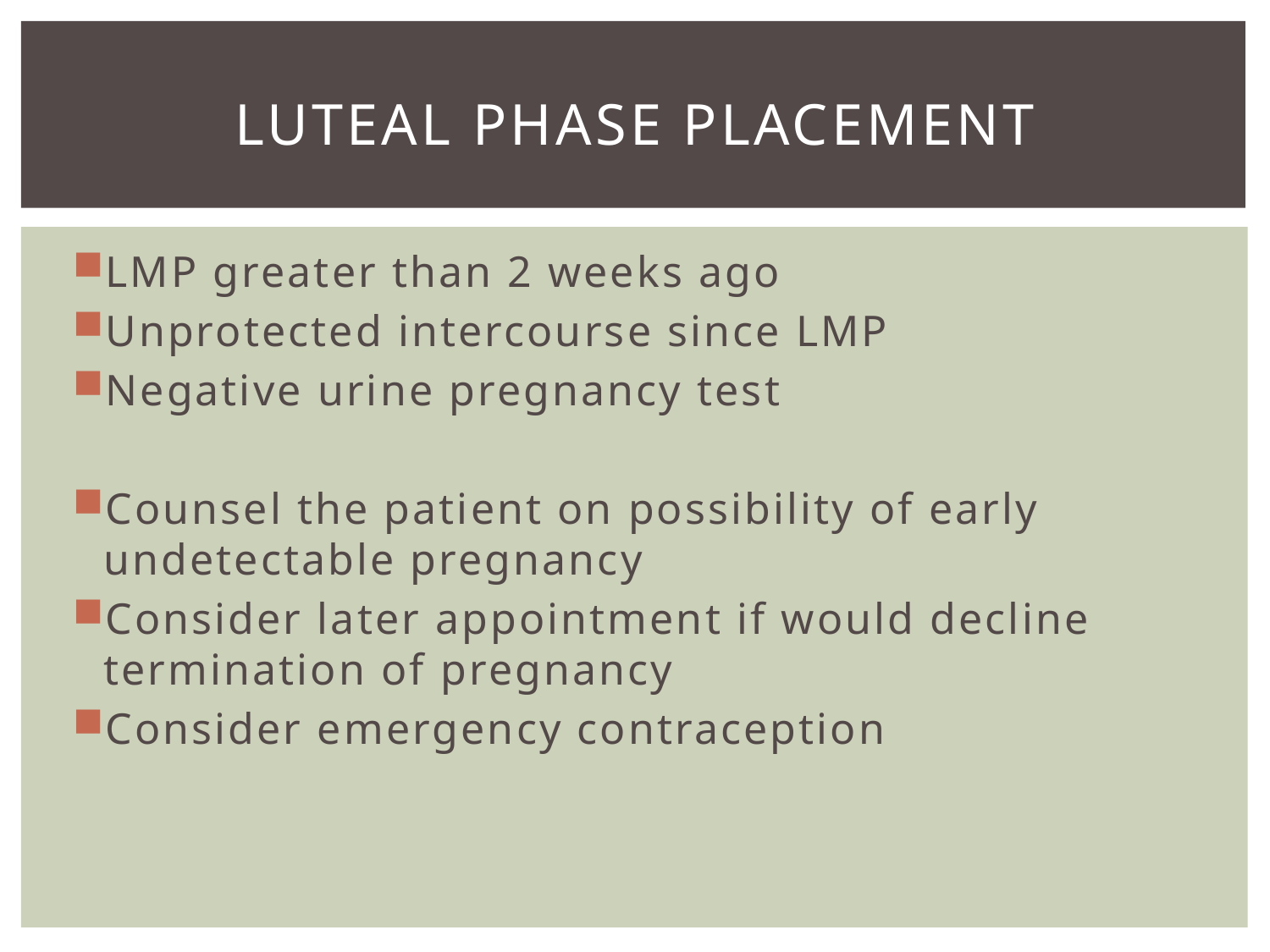

# Luteal Phase Placement
LMP greater than 2 weeks ago
Unprotected intercourse since LMP
Negative urine pregnancy test
Counsel the patient on possibility of early undetectable pregnancy
Consider later appointment if would decline termination of pregnancy
Consider emergency contraception

## Slide 38
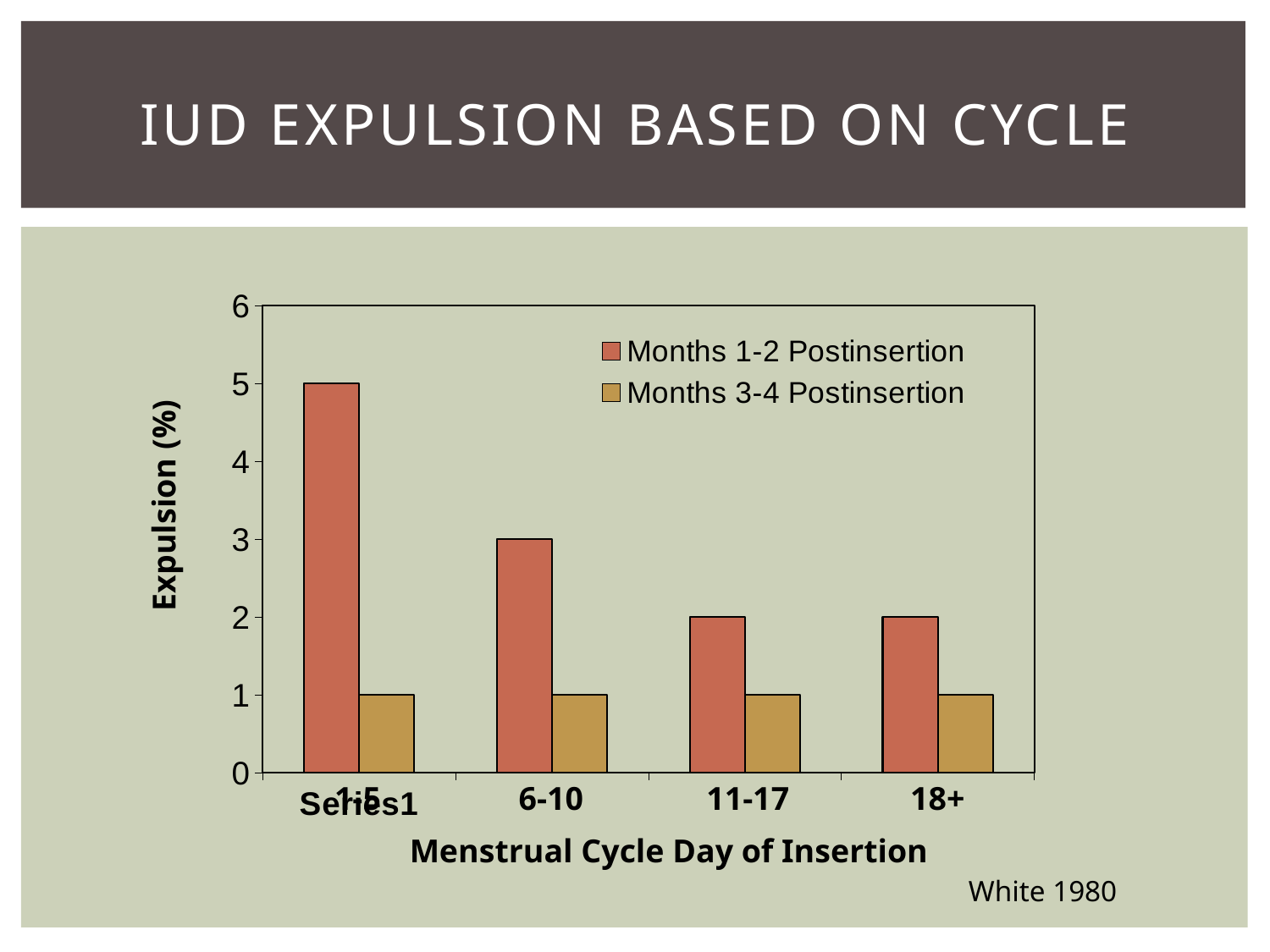

# IUD Expulsion Based on Cycle
### Chart
| Category | Months 1-2 Postinsertion | Months 3-4 Postinsertion |
|---|---|---|
| | 5.0 | 1.0 |
| | 3.0 | 1.0 |
| | 2.0 | 1.0 |
| | 2.0 | 1.0 |Expulsion (%)
1-5
6-10
11-17
18+
Menstrual Cycle Day of Insertion
White 1980

## Slide 39
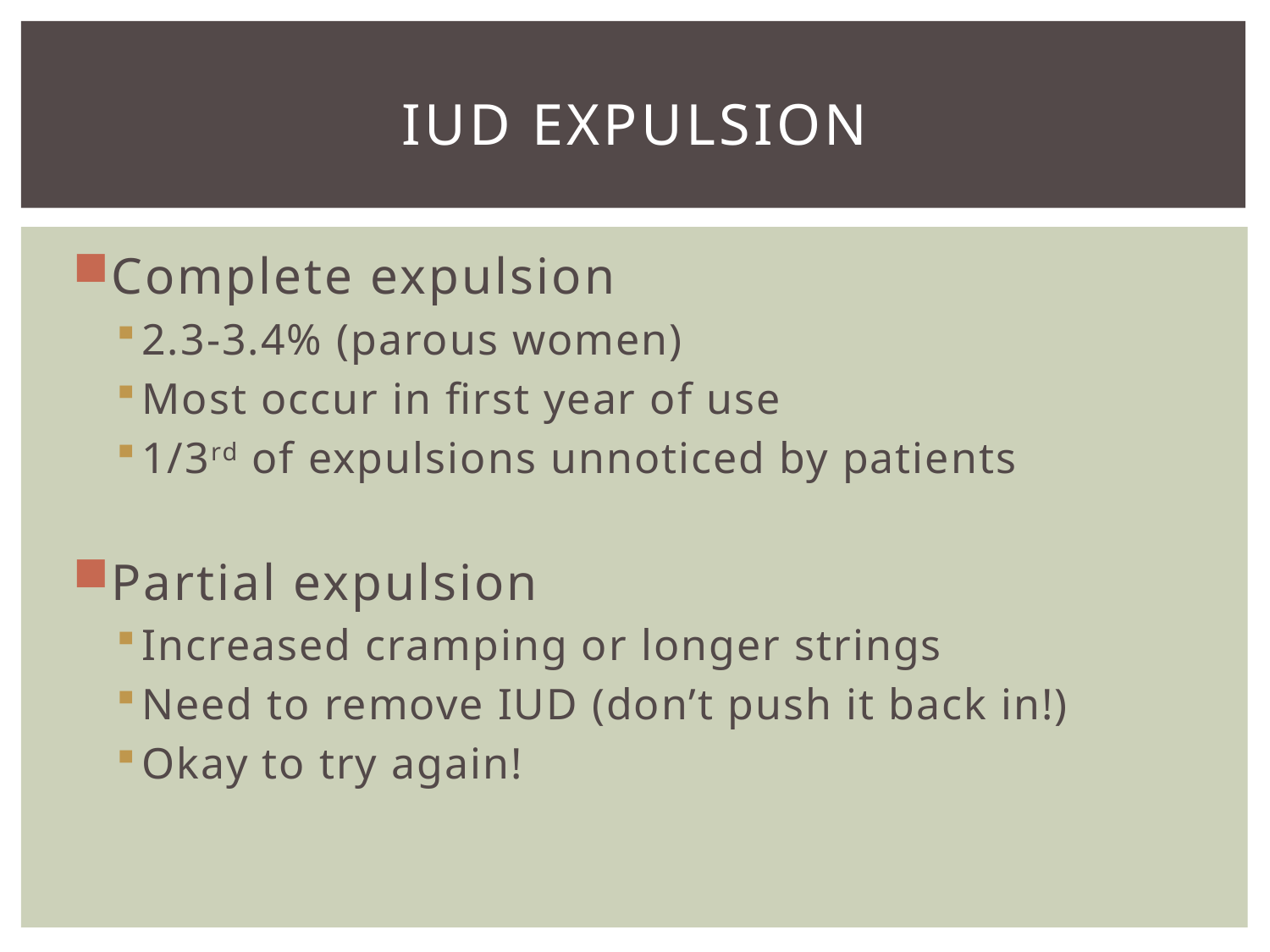

# IUD Expulsion
Complete expulsion
2.3-3.4% (parous women)
Most occur in first year of use
1/3rd of expulsions unnoticed by patients
Partial expulsion
Increased cramping or longer strings
Need to remove IUD (don’t push it back in!)
Okay to try again!

## Slide 40
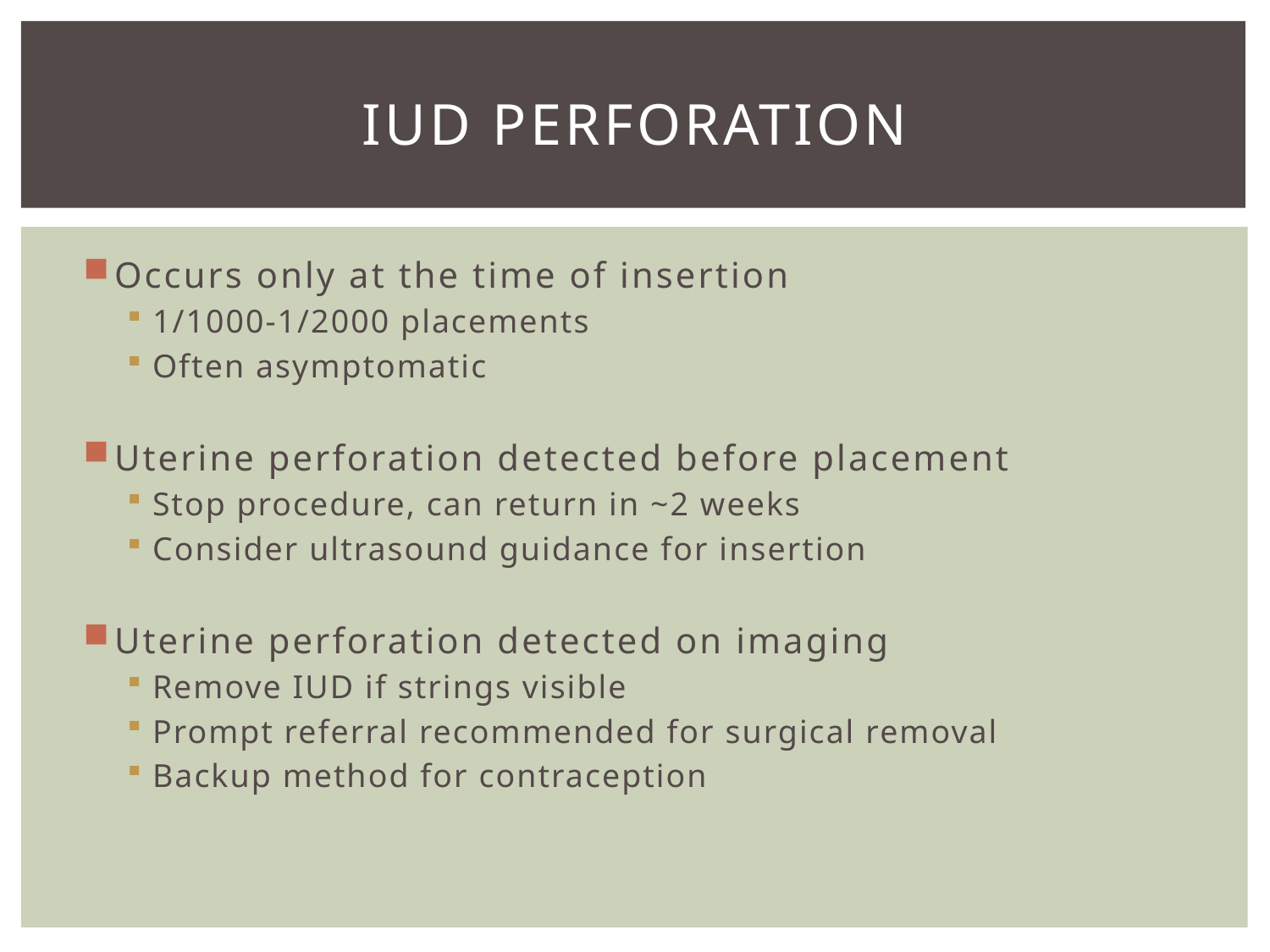

# IUD Perforation
Occurs only at the time of insertion
1/1000-1/2000 placements
Often asymptomatic
Uterine perforation detected before placement
Stop procedure, can return in ~2 weeks
Consider ultrasound guidance for insertion
Uterine perforation detected on imaging
Remove IUD if strings visible
Prompt referral recommended for surgical removal
Backup method for contraception

## Slide 41
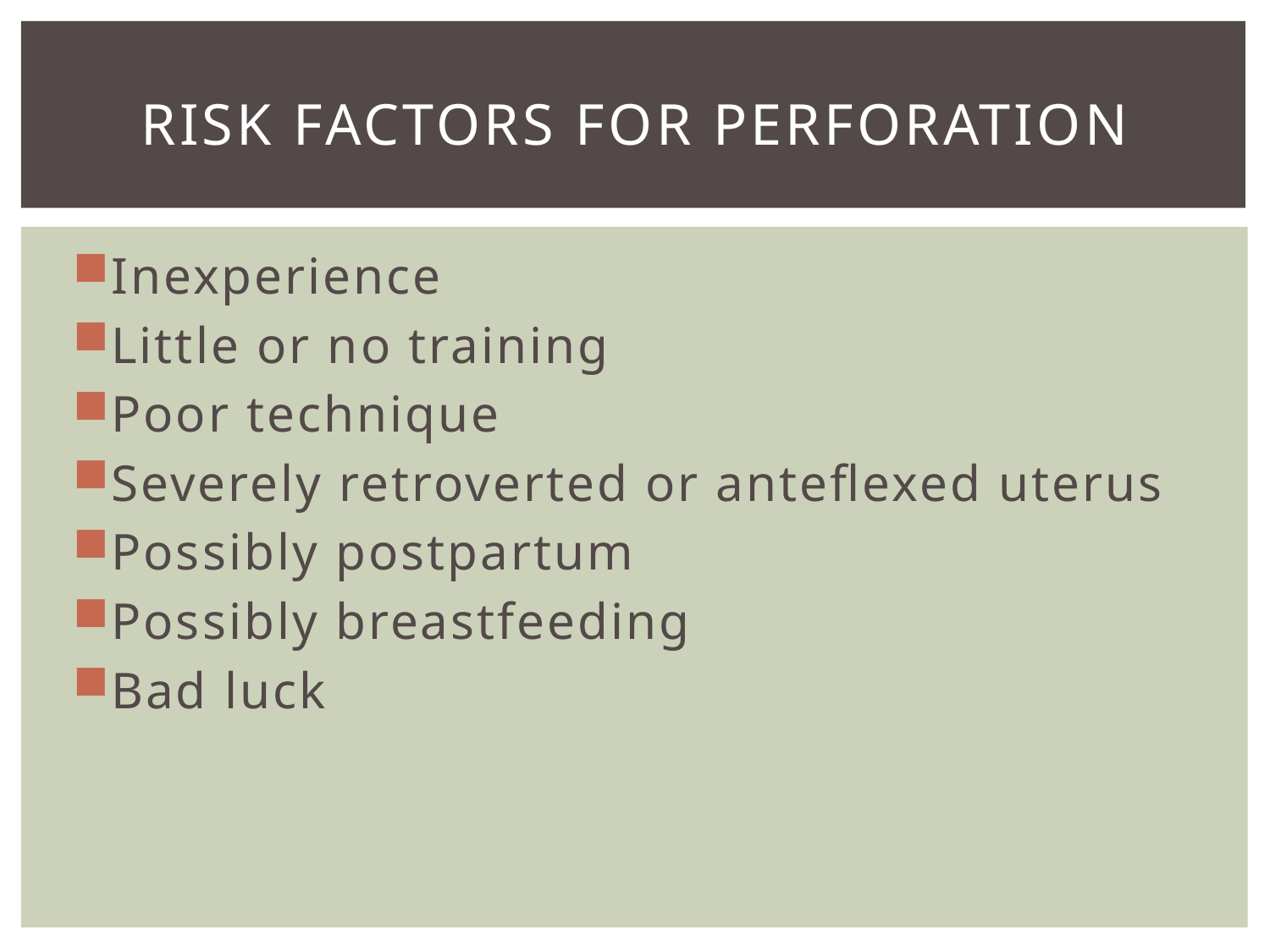

# Risk Factors for Perforation
Inexperience
Little or no training
Poor technique
Severely retroverted or anteflexed uterus
Possibly postpartum
Possibly breastfeeding
Bad luck

## Slide 42
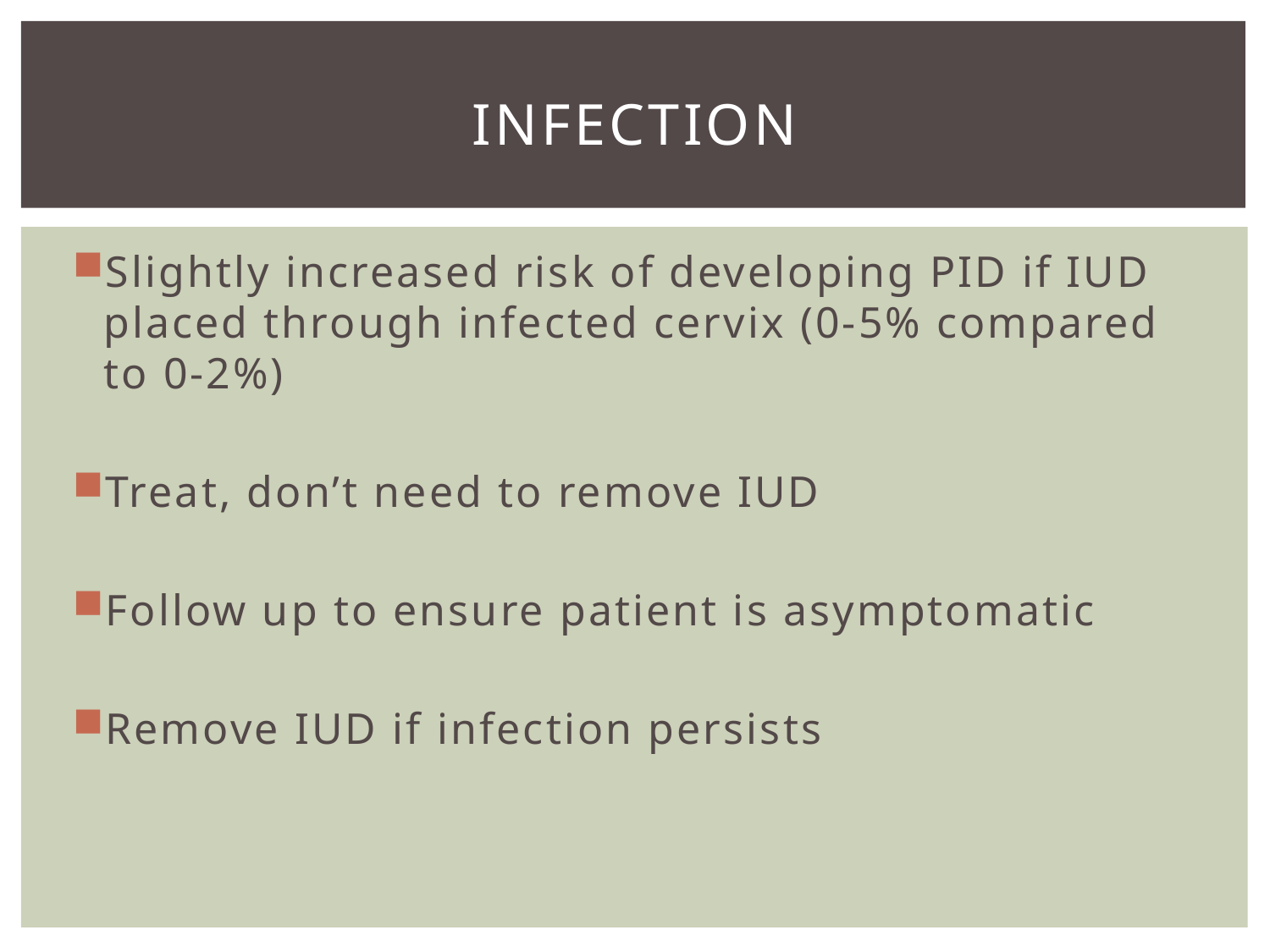

# Infection
Slightly increased risk of developing PID if IUD placed through infected cervix (0-5% compared to 0-2%)
Treat, don’t need to remove IUD
Follow up to ensure patient is asymptomatic
Remove IUD if infection persists

## Slide 43
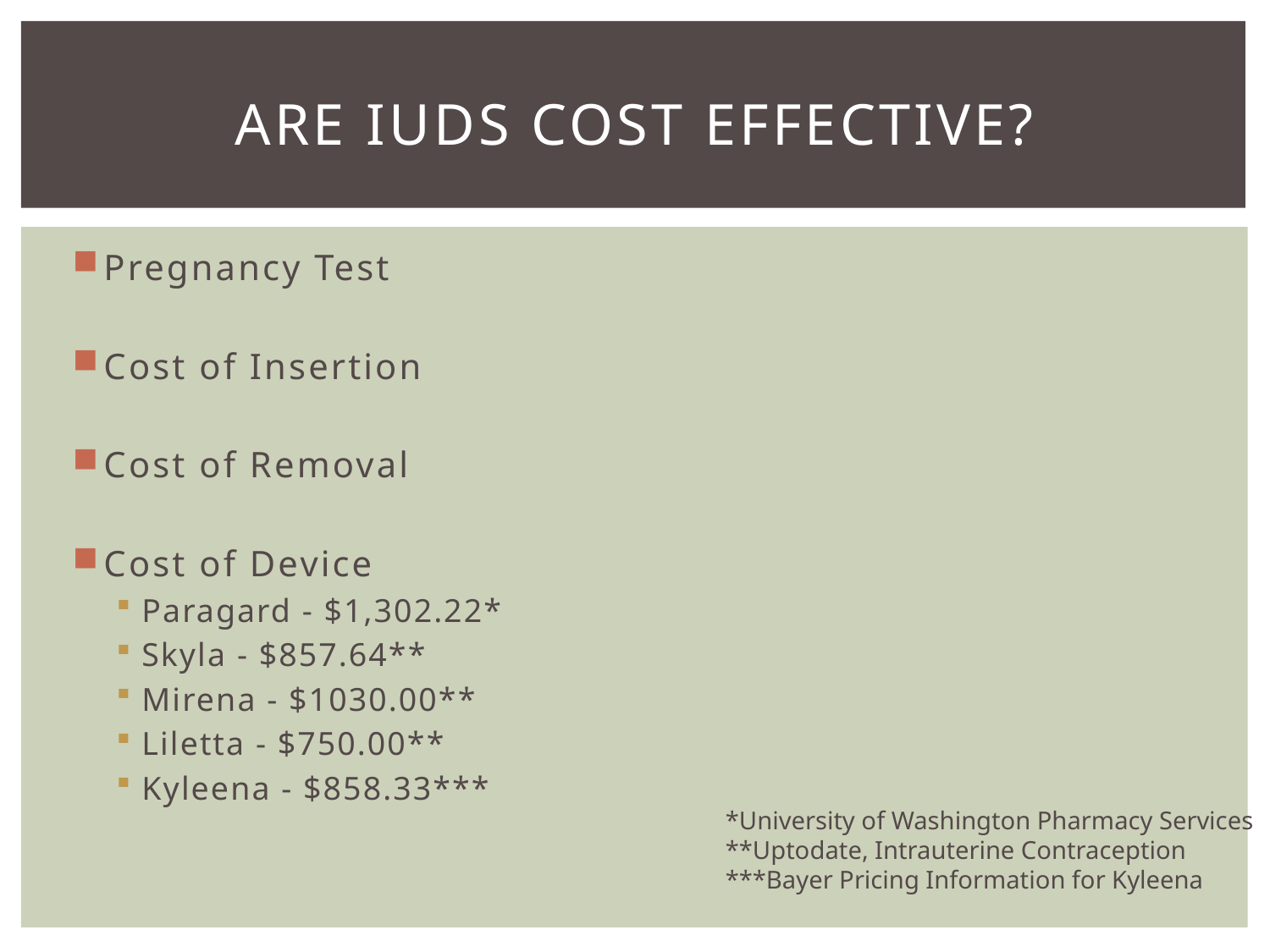

# Are IUDs cost effective?
Pregnancy Test
Cost of Insertion
Cost of Removal
Cost of Device
Paragard - $1,302.22*
Skyla - $857.64**
Mirena - $1030.00**
Liletta - $750.00**
Kyleena - $858.33***
*University of Washington Pharmacy Services
**Uptodate, Intrauterine Contraception
***Bayer Pricing Information for Kyleena

## Slide 44
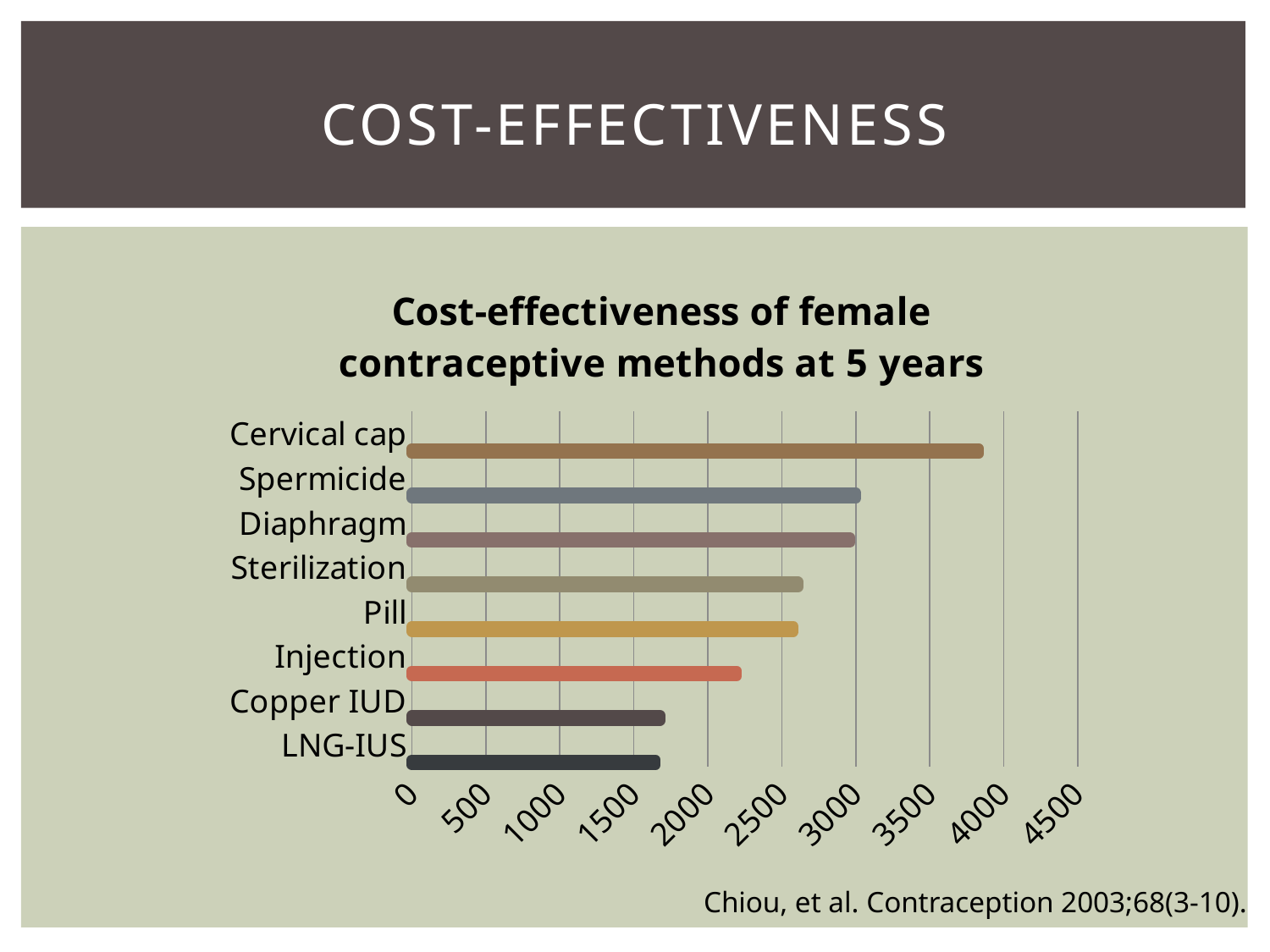

# Cost-effectiveness
### Chart: Cost-effectiveness of female contraceptive methods at 5 years
| Category | #REF! | | | | | | | |
|---|---|---|---|---|---|---|---|---|
| LNG-IUS | 1646.2 | None | None | None | None | None | None | None |
| Copper IUD | 1677.6 | None | None | None | None | None | None | None |
| Injection | 2194.5 | None | None | None | None | None | None | None |
| Pill | 2578.0 | None | None | None | None | None | None | None |
| Sterilization | 2611.0 | None | None | None | None | None | None | None |
| Diaphragm | 2959.5 | None | None | None | None | None | None | None |
| Spermicide | 3002.2 | None | None | None | None | None | None | None |
| Cervical cap | 3831.3 | None | None | None | None | None | None | None |Chiou, et al. Contraception 2003;68(3-10).

## Slide 45
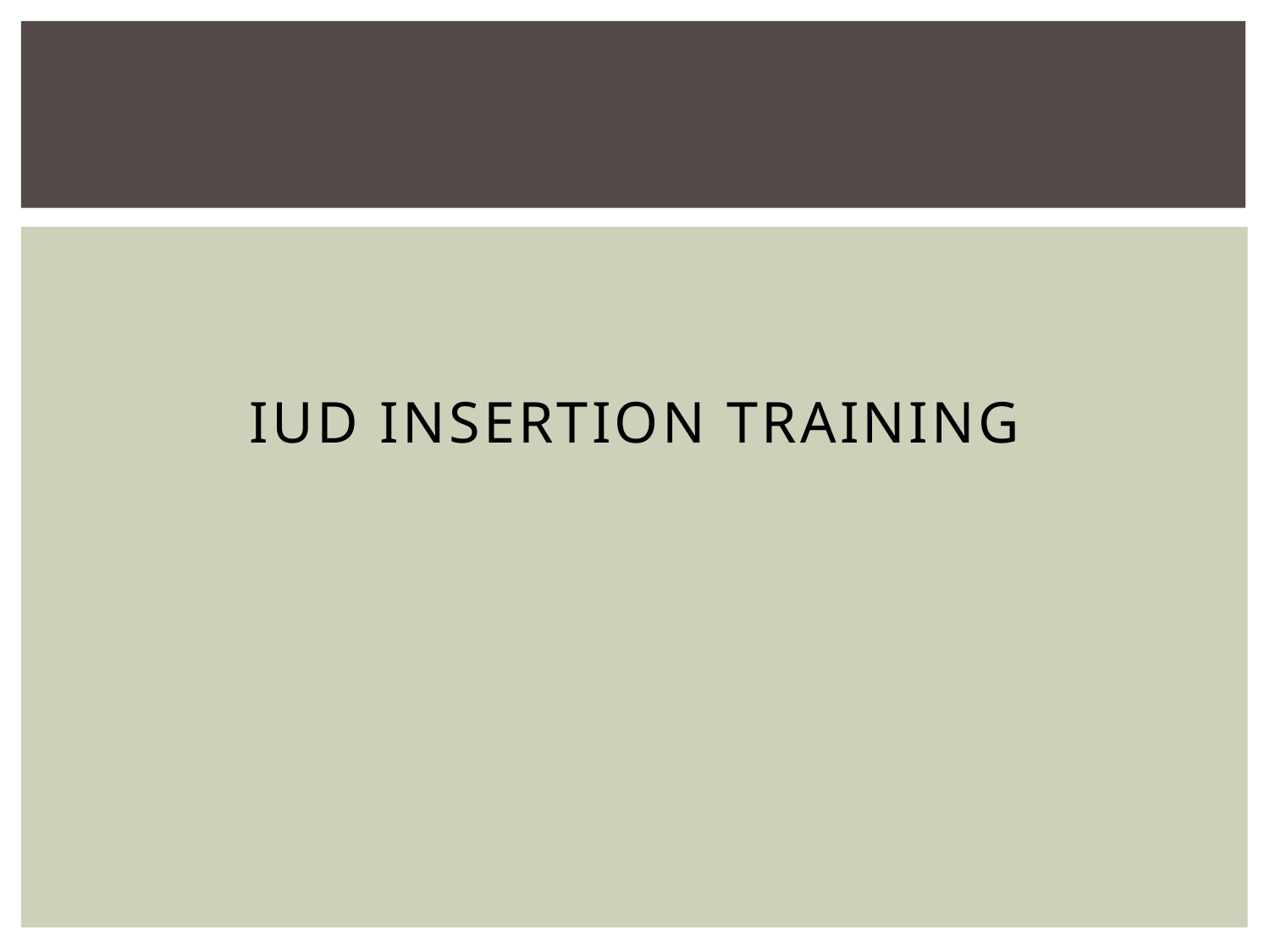

# Iud insertion training

## Slide 46
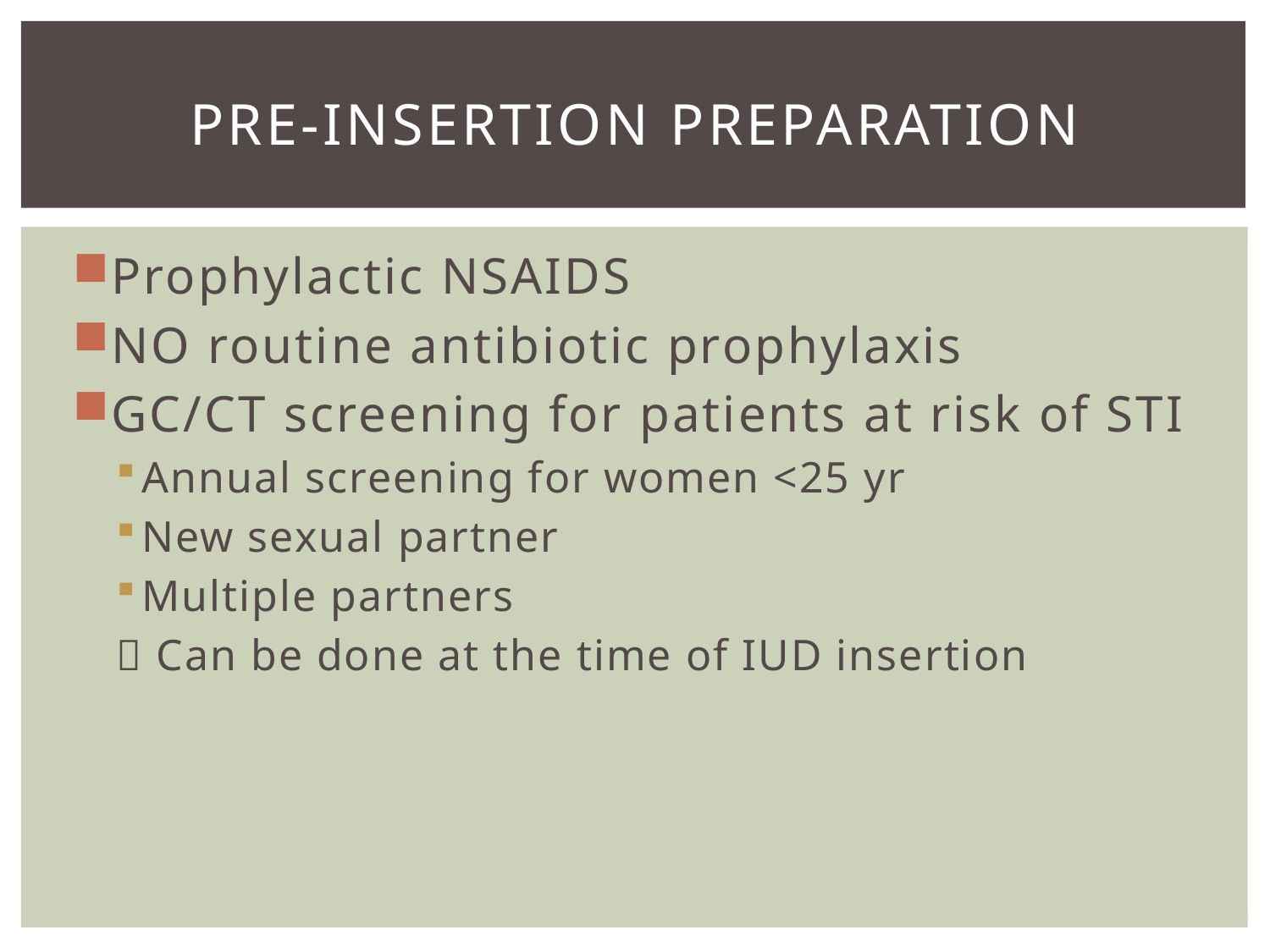

# Pre-Insertion Preparation
Prophylactic NSAIDS
NO routine antibiotic prophylaxis
GC/CT screening for patients at risk of STI
Annual screening for women <25 yr
New sexual partner
Multiple partners
 Can be done at the time of IUD insertion

## Slide 47
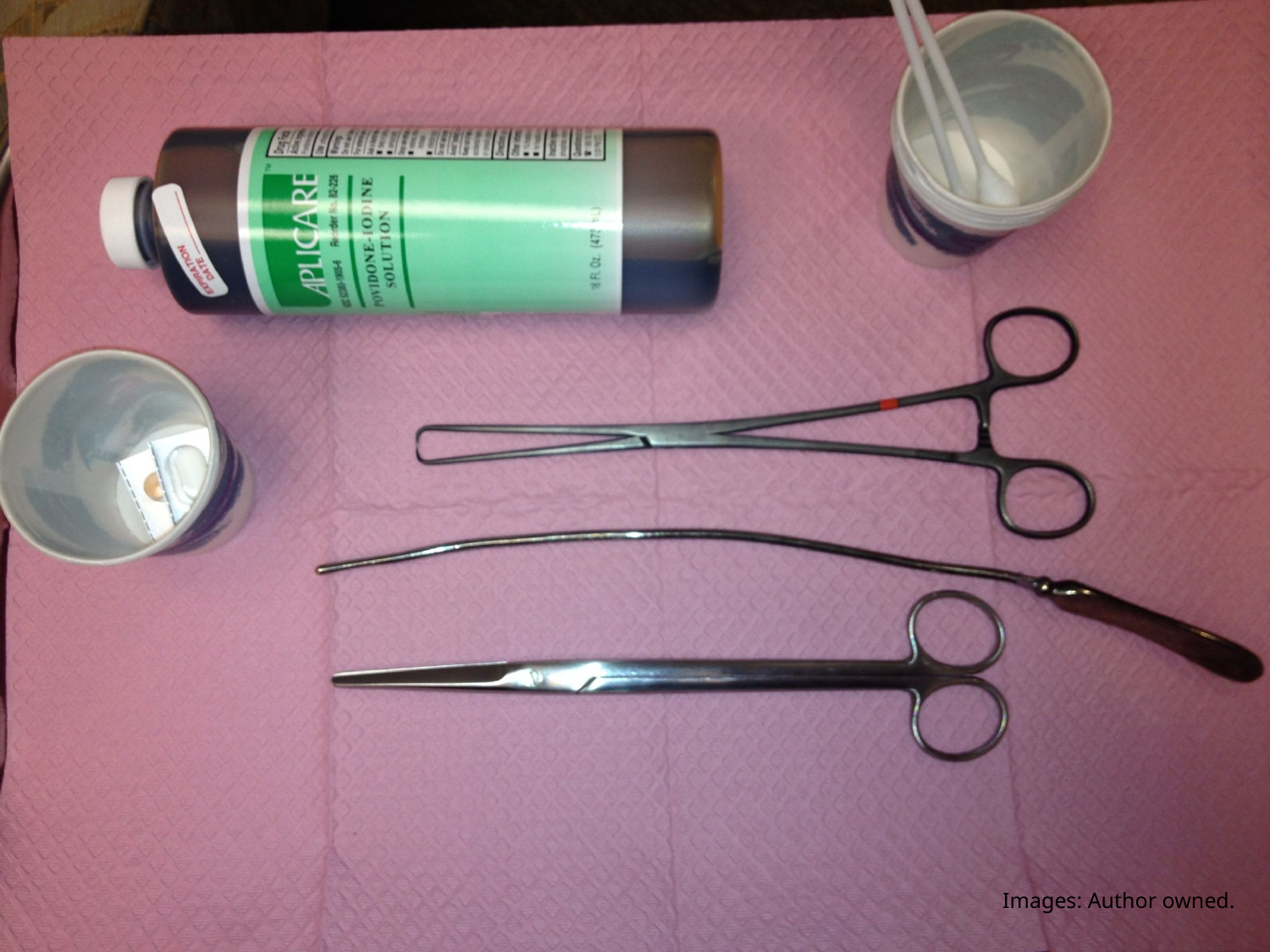

Tray Setup
Images: Author owned.

## Slide 48
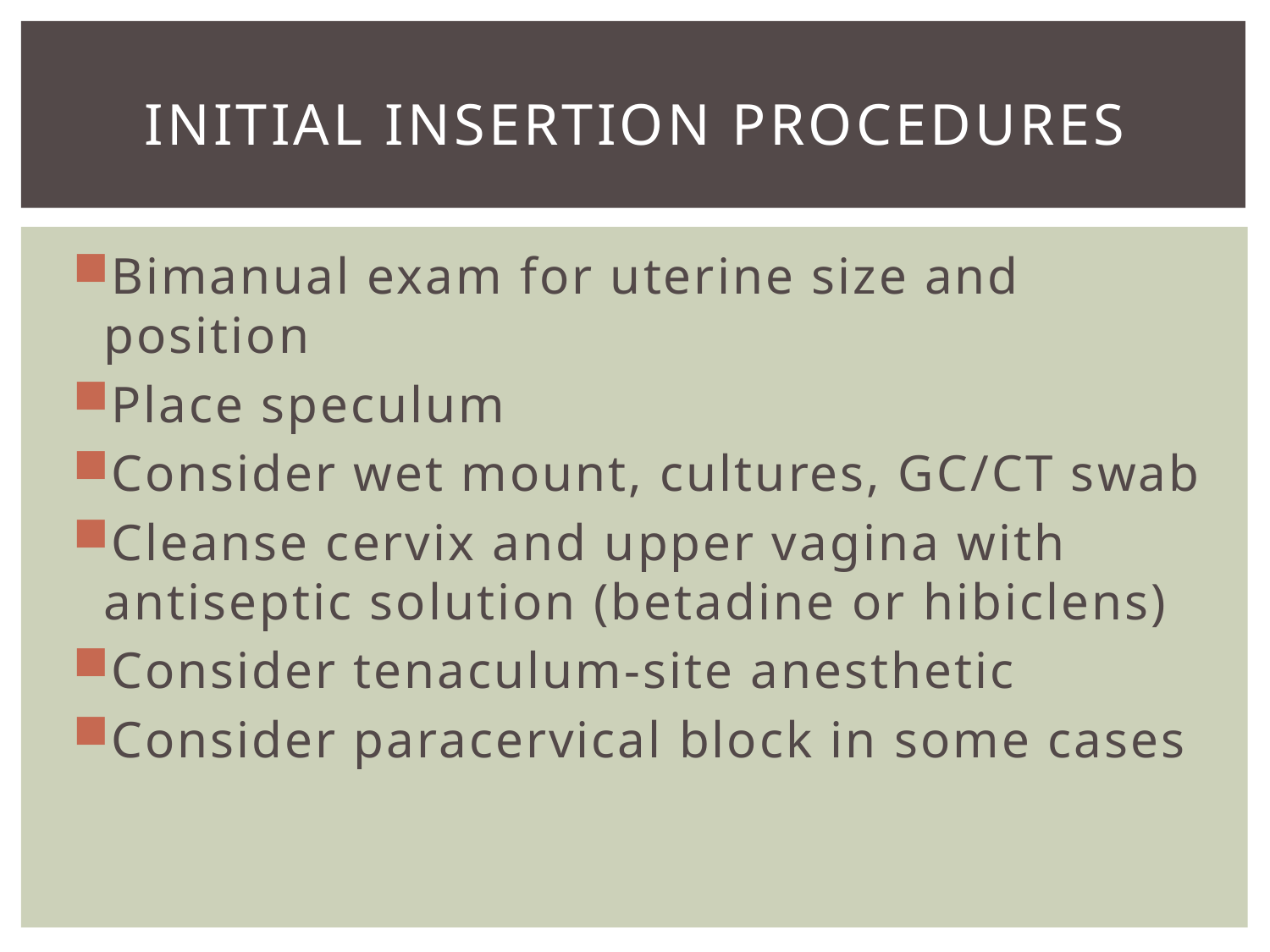

# Initial Insertion Procedures
Bimanual exam for uterine size and position
Place speculum
Consider wet mount, cultures, GC/CT swab
Cleanse cervix and upper vagina with antiseptic solution (betadine or hibiclens)
Consider tenaculum-site anesthetic
Consider paracervical block in some cases

## Slide 49
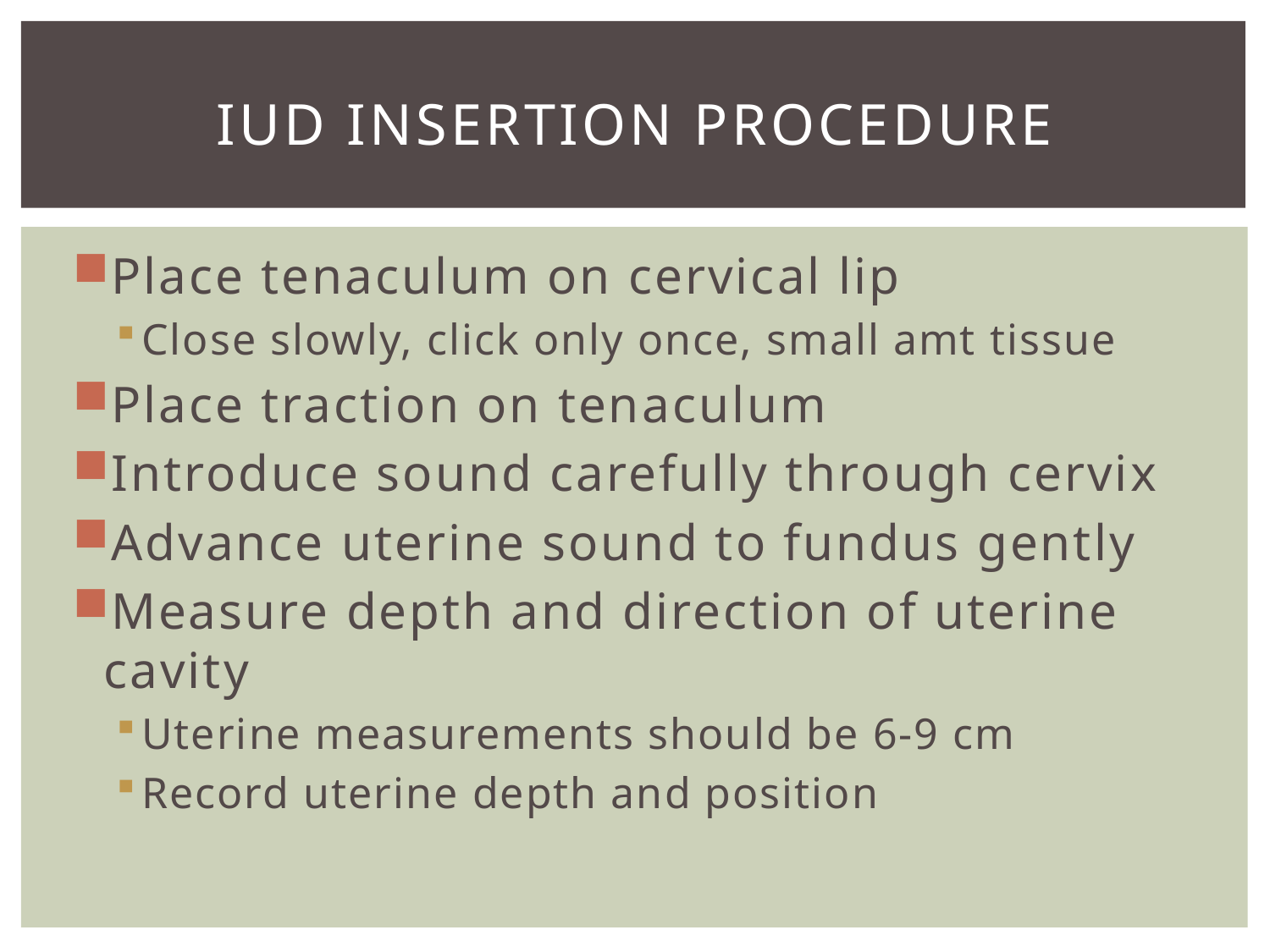

# IUD Insertion Procedure
Place tenaculum on cervical lip
Close slowly, click only once, small amt tissue
Place traction on tenaculum
Introduce sound carefully through cervix
Advance uterine sound to fundus gently
Measure depth and direction of uterine cavity
Uterine measurements should be 6-9 cm
Record uterine depth and position

## Slide 50
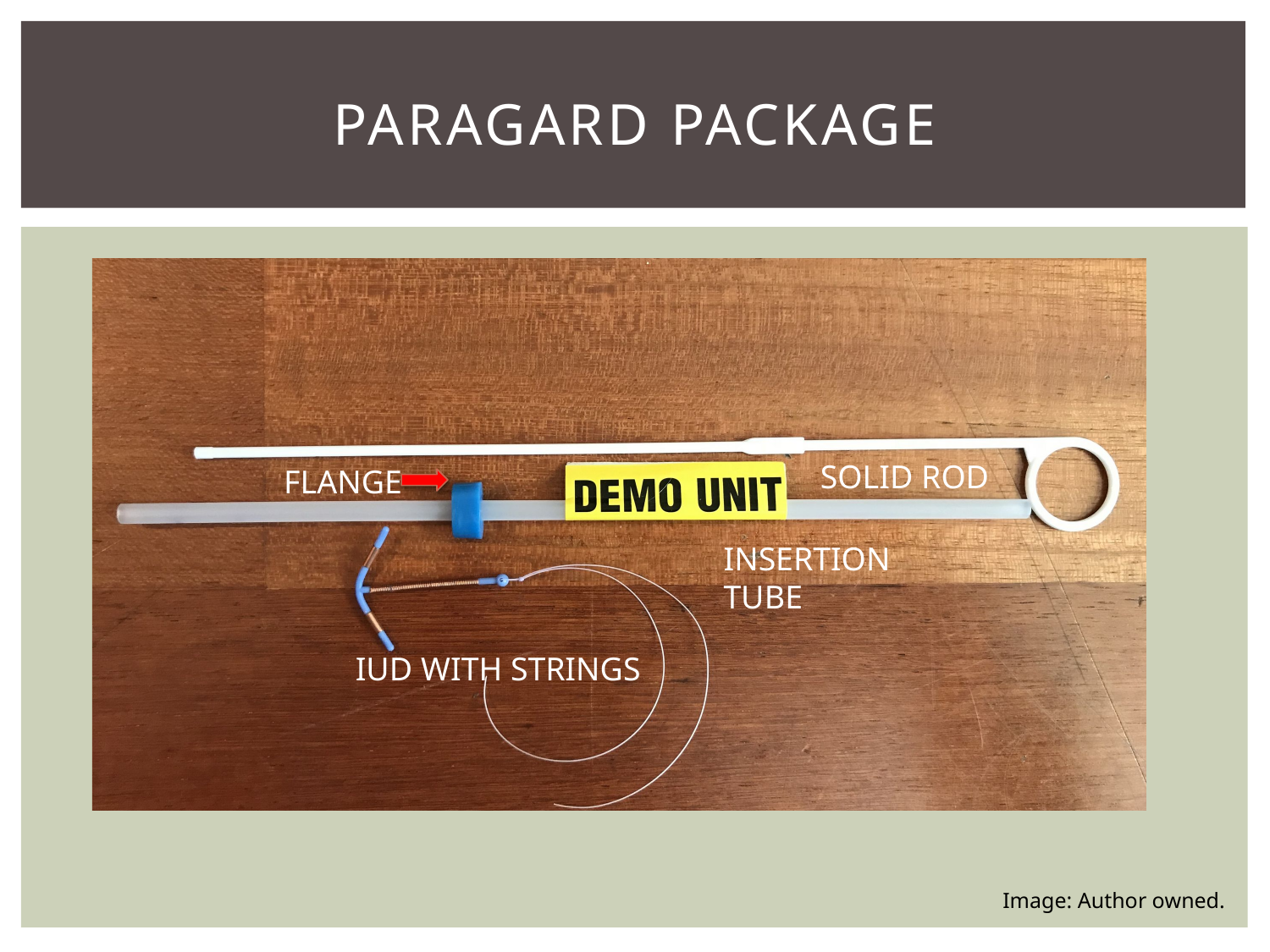

# Paragard package
SOLID ROD
FLANGE
INSERTION TUBE
IUD WITH STRINGS
Image: Author owned.

## Slide 51
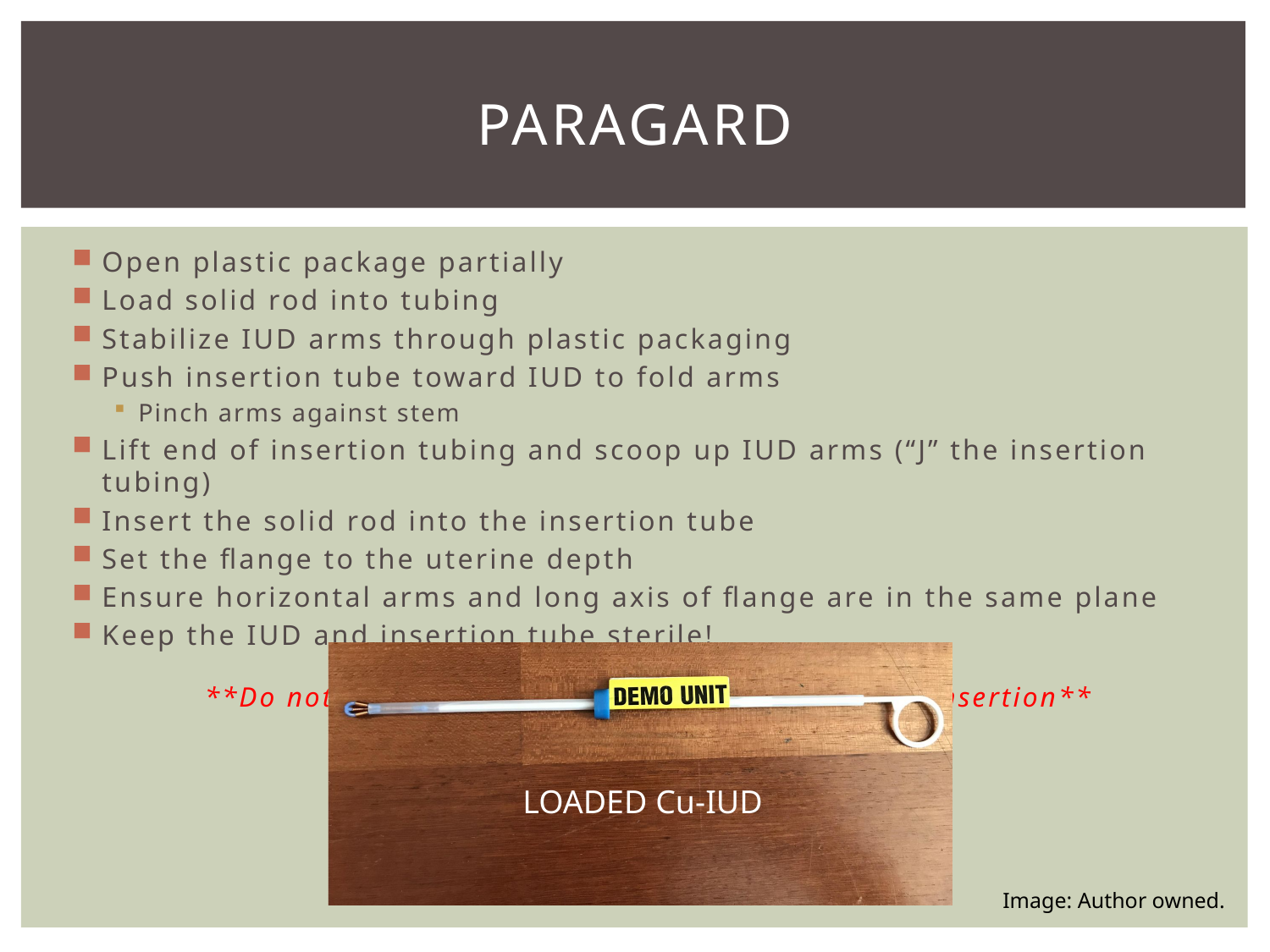

# Paragard
Open plastic package partially
Load solid rod into tubing
Stabilize IUD arms through plastic packaging
Push insertion tube toward IUD to fold arms
Pinch arms against stem
Lift end of insertion tubing and scoop up IUD arms (“J” the insertion tubing)
Insert the solid rod into the insertion tube
Set the flange to the uterine depth
Ensure horizontal arms and long axis of flange are in the same plane
Keep the IUD and insertion tube sterile!
**Do not bend arms more than 5 minutes prior to insertion**
LOADED Cu-IUD
Image: Author owned.

## Slide 52
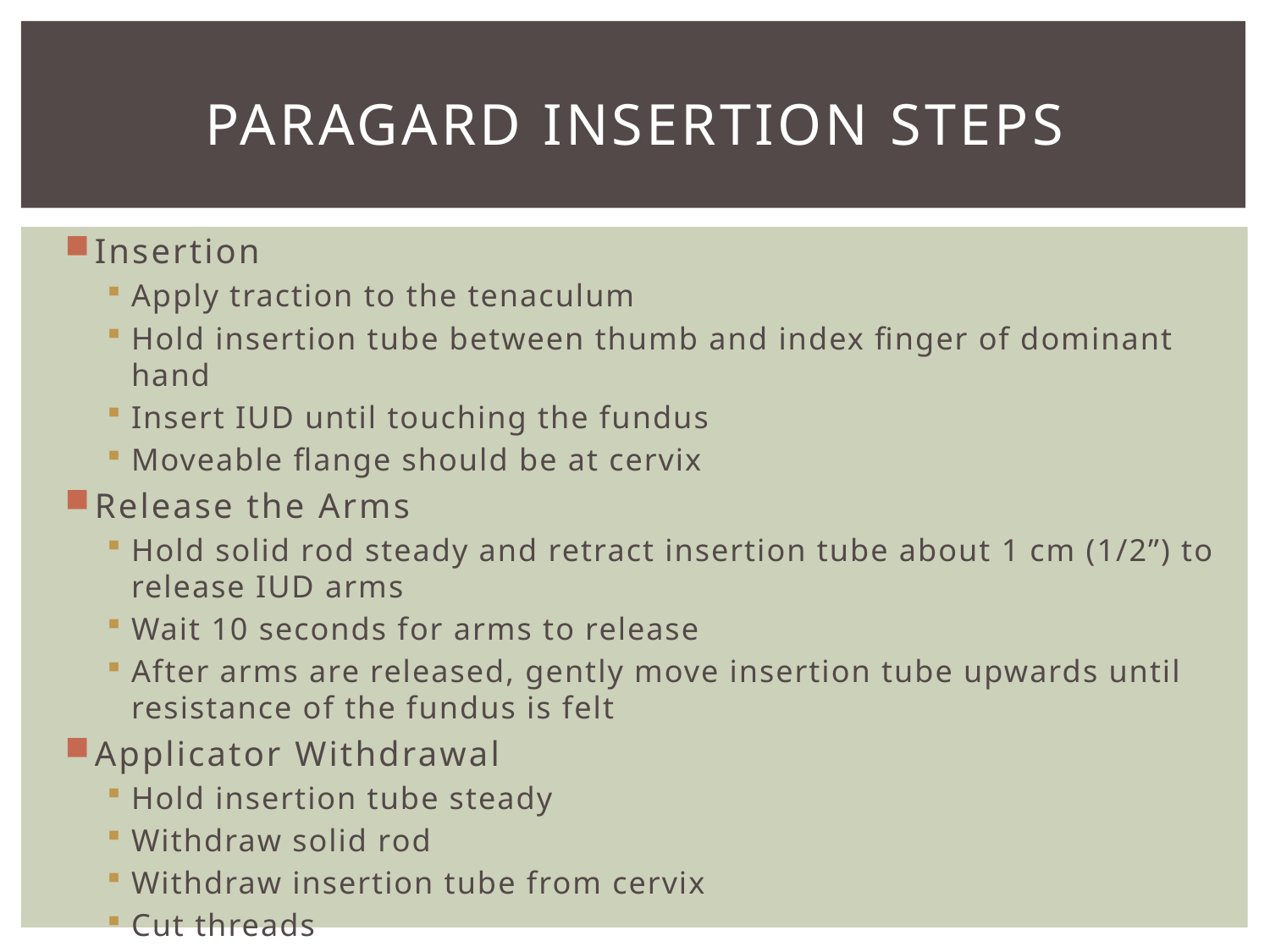

# Paragard insertion steps
Insertion
Apply traction to the tenaculum
Hold insertion tube between thumb and index finger of dominant hand
Insert IUD until touching the fundus
Moveable flange should be at cervix
Release the Arms
Hold solid rod steady and retract insertion tube about 1 cm (1/2”) to release IUD arms
Wait 10 seconds for arms to release
After arms are released, gently move insertion tube upwards until resistance of the fundus is felt
Applicator Withdrawal
Hold insertion tube steady
Withdraw solid rod
Withdraw insertion tube from cervix
Cut threads

## Slide 53
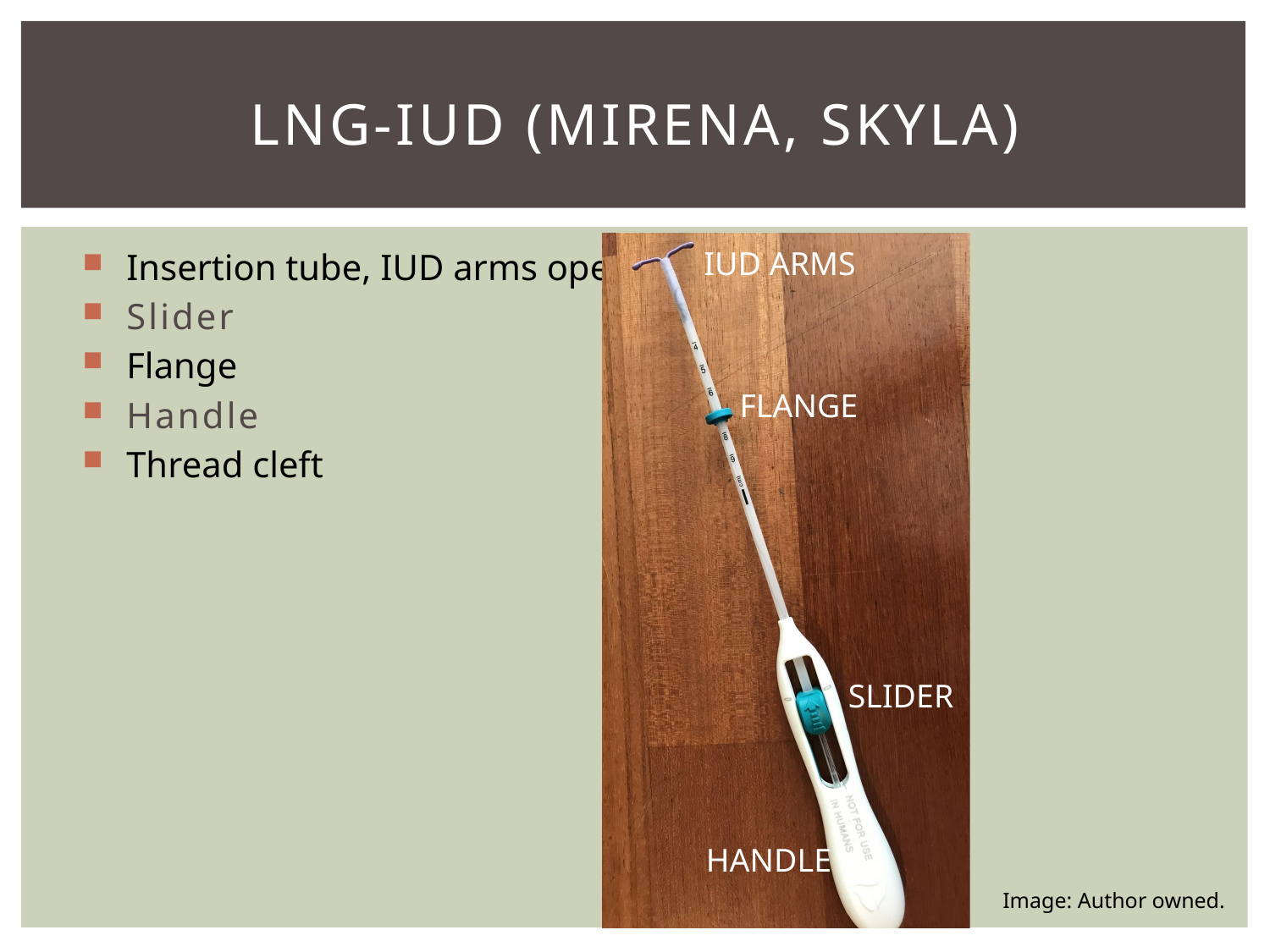

# LNG-Iud (mirena, skyla)
IUD ARMS
FLANGE
SLIDER
HANDLE
Insertion tube, IUD arms open
Slider
Flange
Handle
Thread cleft
Image: Author owned.

## Slide 54
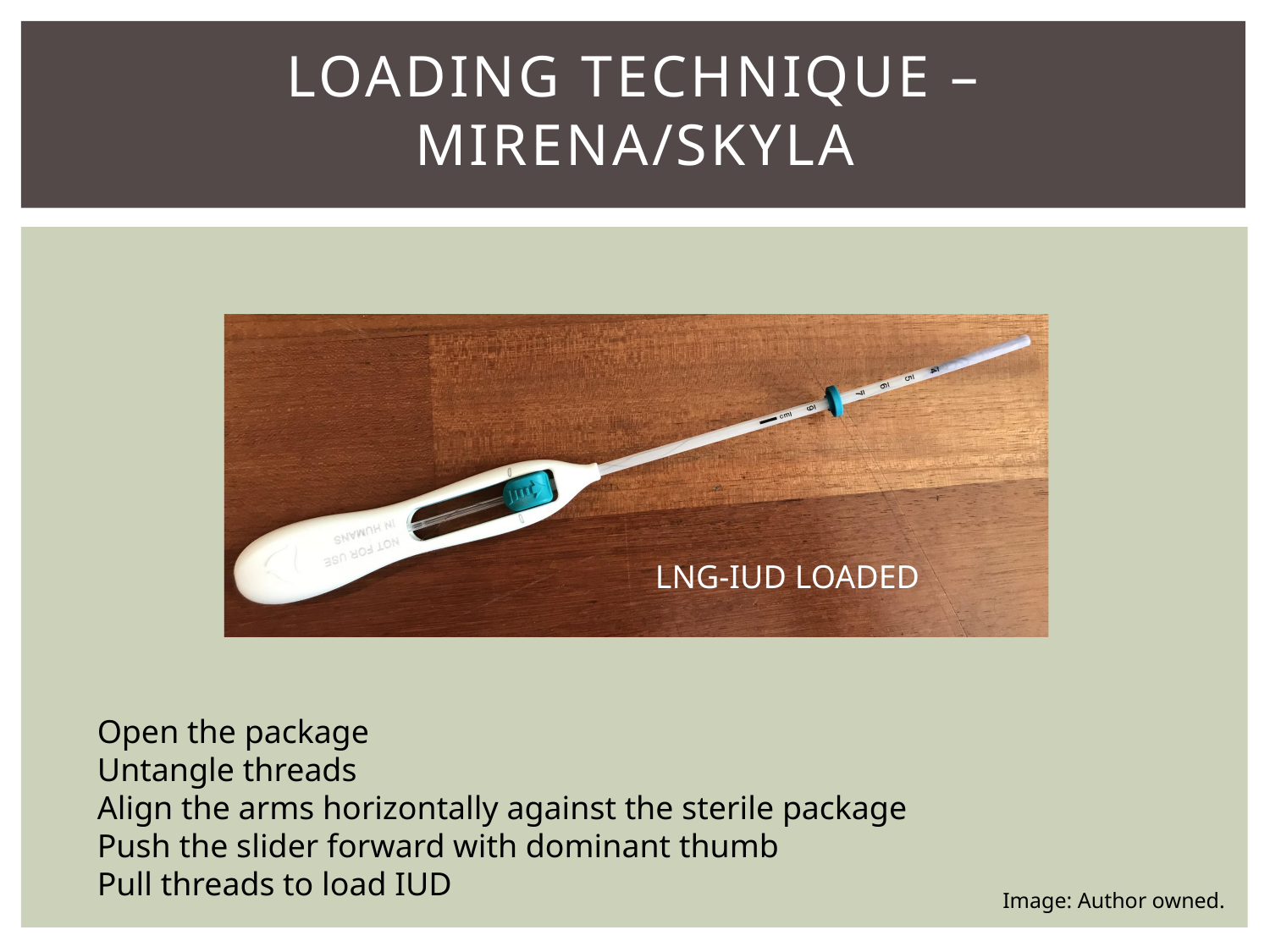

# Loading Technique – mirena/skyla
 Keep your thumb on the slider!
LNG-IUD LOADED
Open the package
Untangle threads
Align the arms horizontally against the sterile package
Push the slider forward with dominant thumb
Pull threads to load IUD
Image: Author owned.

## Slide 55
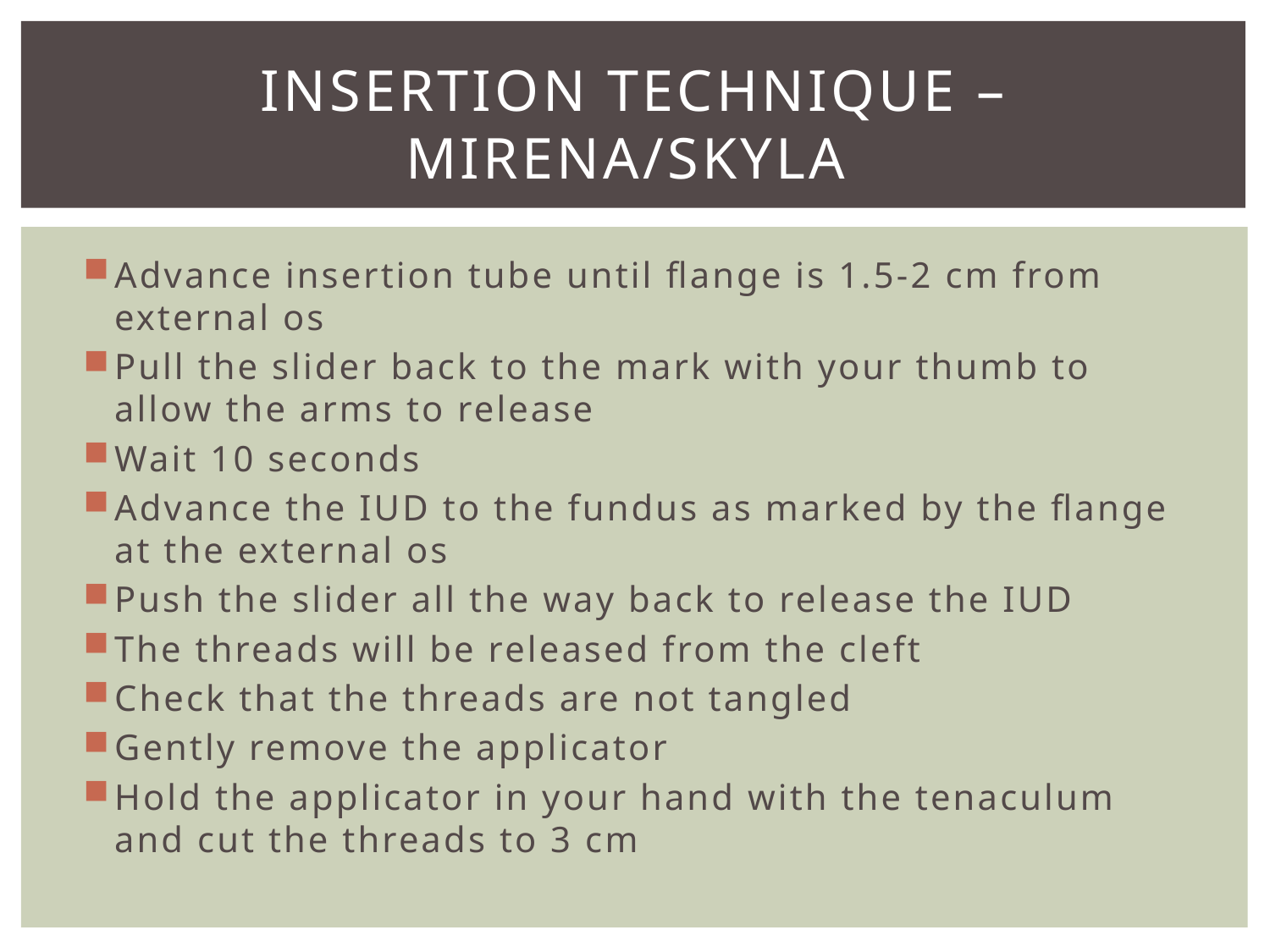

# Insertion Technique – mirena/skyla
Advance insertion tube until flange is 1.5-2 cm from external os
Pull the slider back to the mark with your thumb to allow the arms to release
Wait 10 seconds
Advance the IUD to the fundus as marked by the flange at the external os
Push the slider all the way back to release the IUD
The threads will be released from the cleft
Check that the threads are not tangled
Gently remove the applicator
Hold the applicator in your hand with the tenaculum and cut the threads to 3 cm

## Slide 56
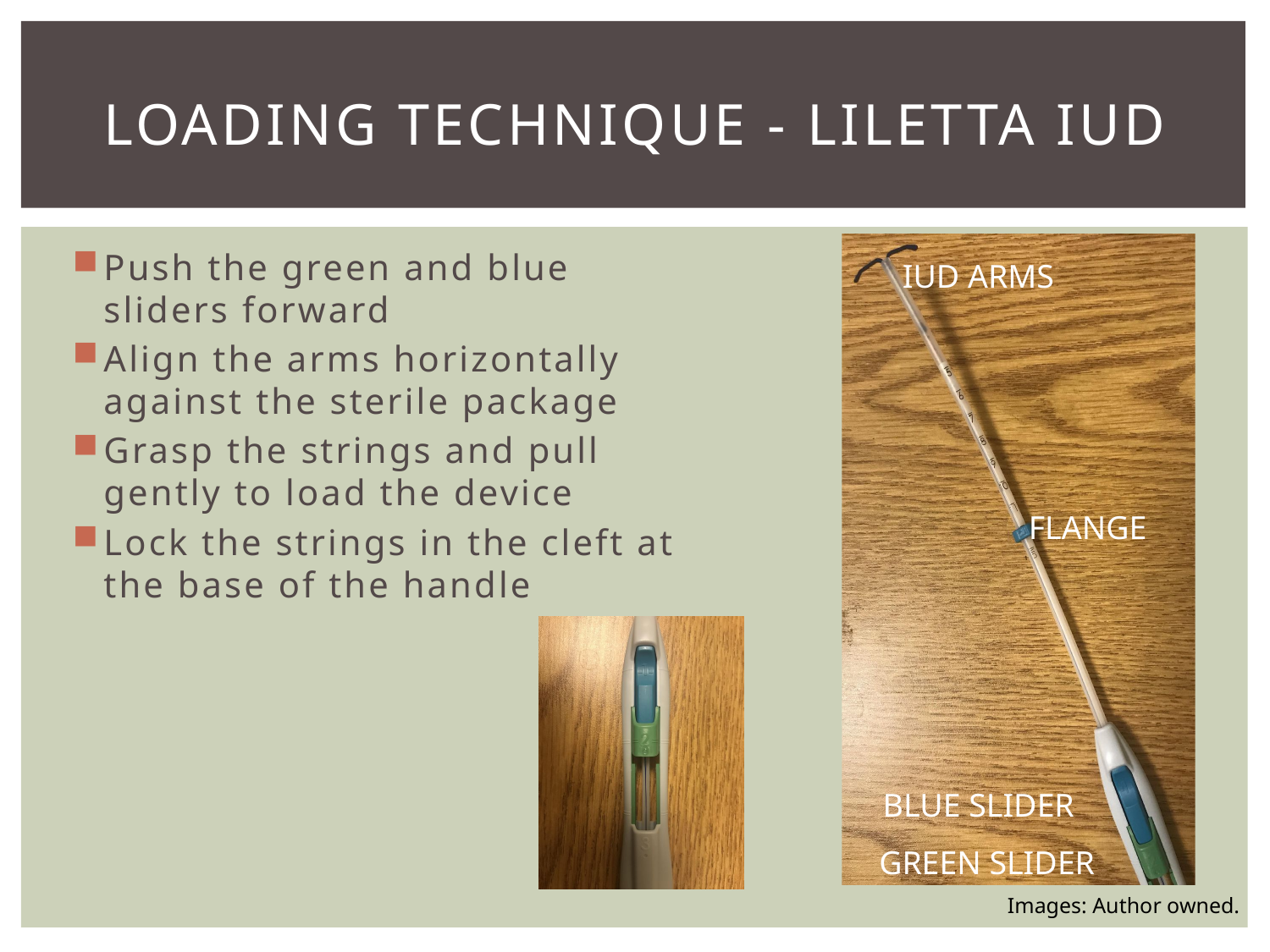

# Loading technique - Liletta iud
IUD ARMS
FLANGE
BLUE SLIDER
GREEN SLIDER
Push the green and blue sliders forward
Align the arms horizontally against the sterile package
Grasp the strings and pull gently to load the device
Lock the strings in the cleft at the base of the handle
Images: Author owned.

## Slide 57
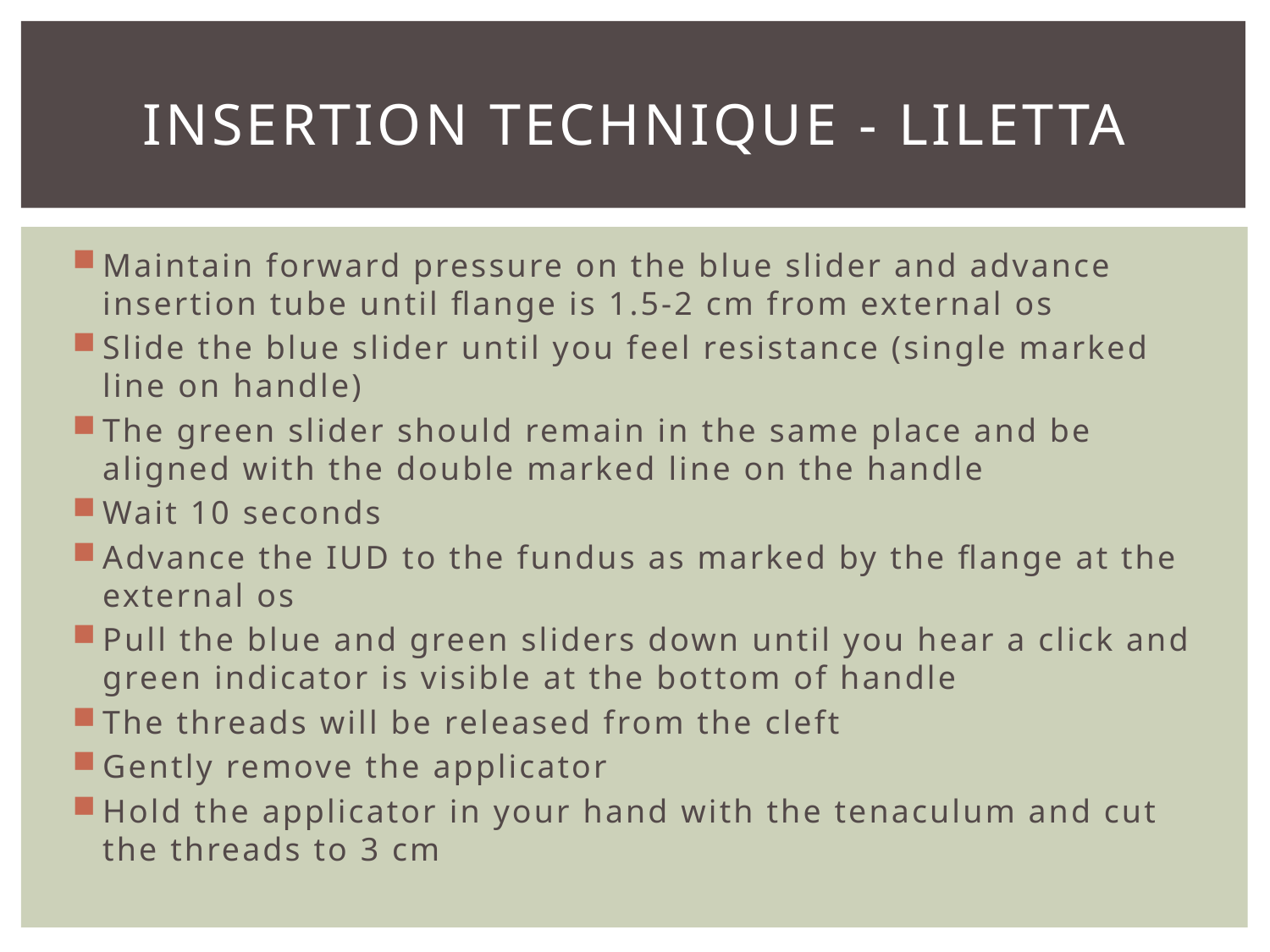

# Insertion Technique - liletta
Maintain forward pressure on the blue slider and advance insertion tube until flange is 1.5-2 cm from external os
Slide the blue slider until you feel resistance (single marked line on handle)
The green slider should remain in the same place and be aligned with the double marked line on the handle
Wait 10 seconds
Advance the IUD to the fundus as marked by the flange at the external os
Pull the blue and green sliders down until you hear a click and green indicator is visible at the bottom of handle
The threads will be released from the cleft
Gently remove the applicator
Hold the applicator in your hand with the tenaculum and cut the threads to 3 cm

## Slide 58
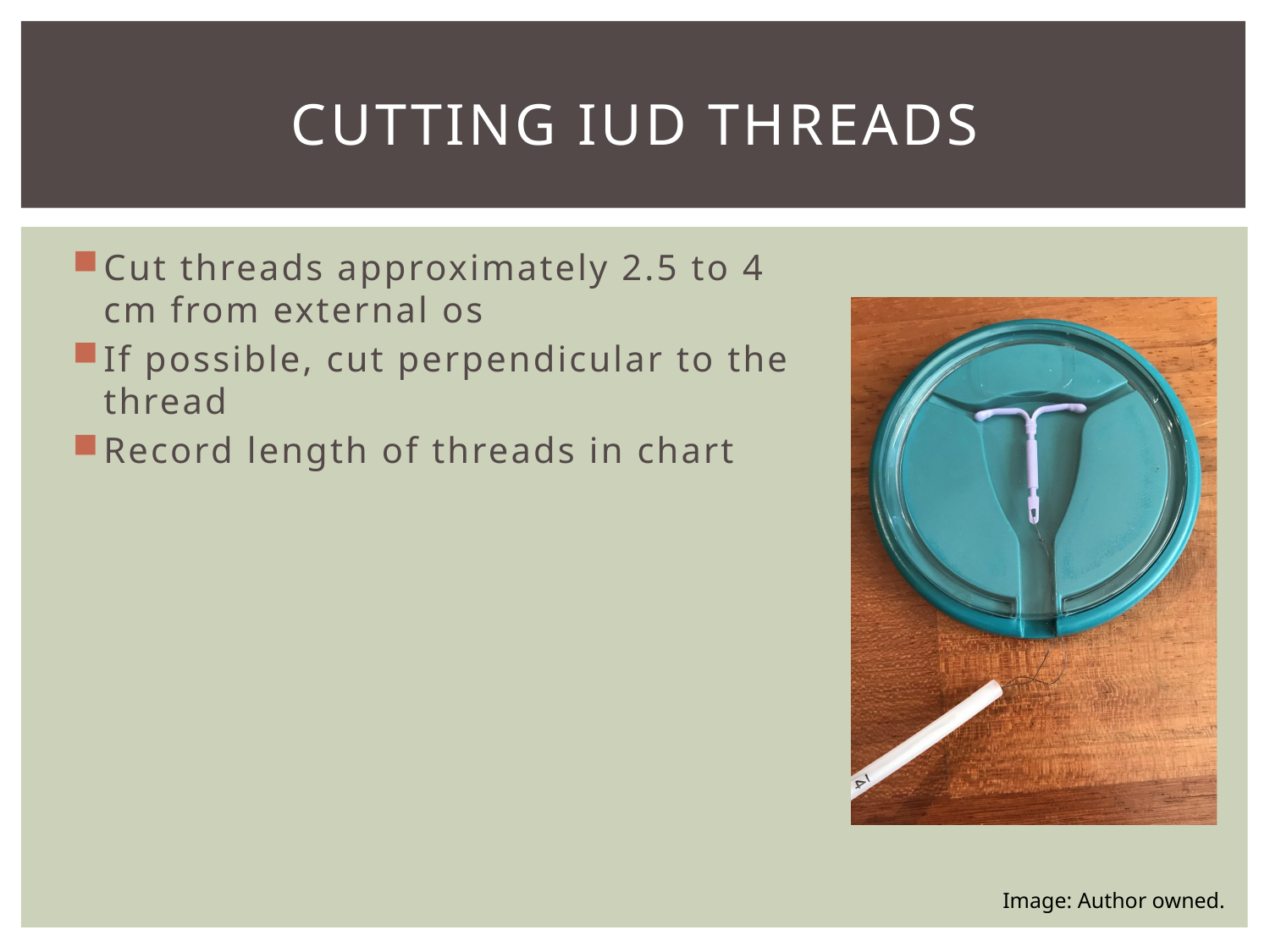

# Cutting IUD Threads
Cut threads approximately 2.5 to 4 cm from external os
If possible, cut perpendicular to the thread
Record length of threads in chart
Image: Author owned.

## Slide 59
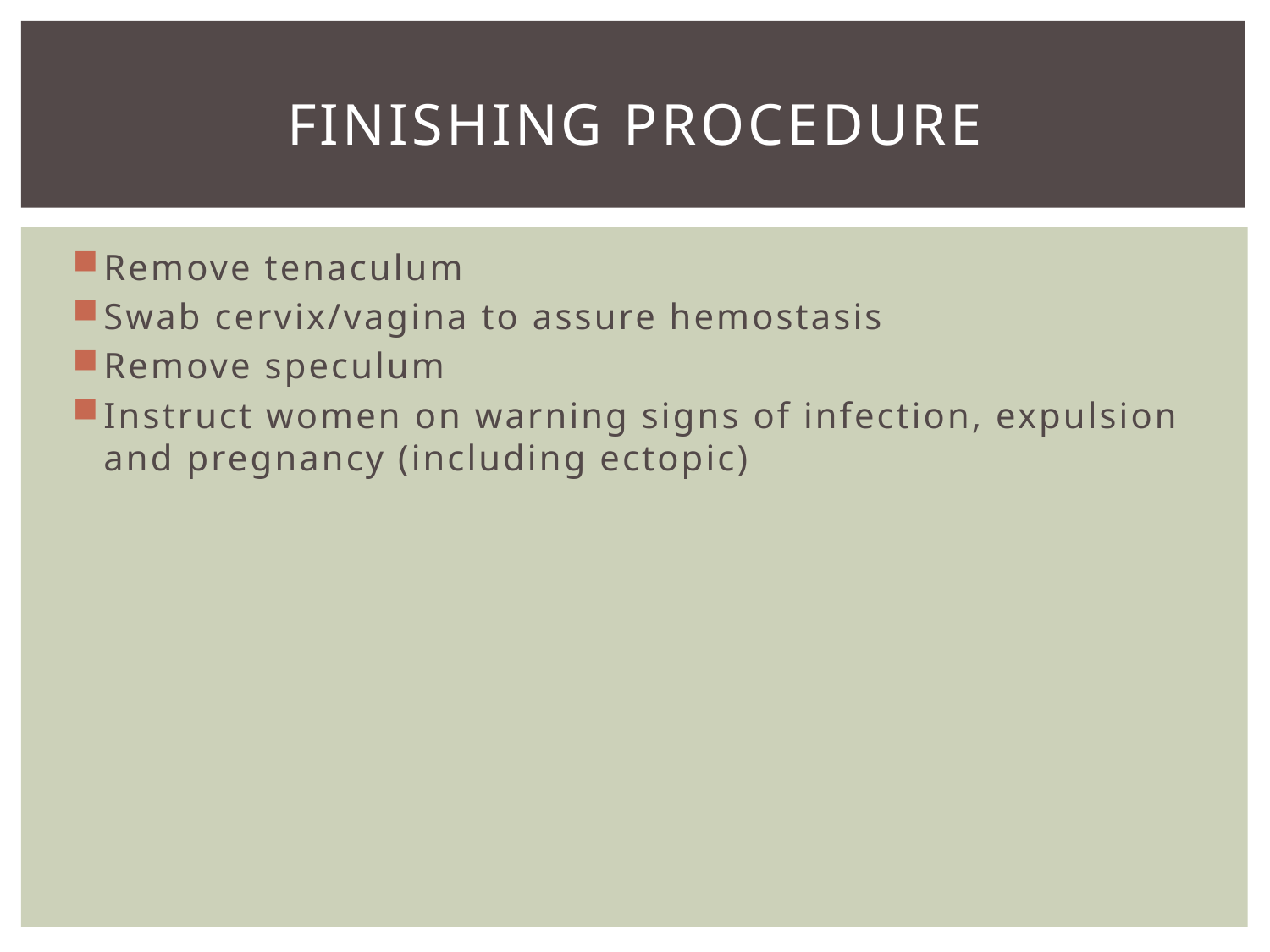

# Finishing procedure
Remove tenaculum
Swab cervix/vagina to assure hemostasis
Remove speculum
Instruct women on warning signs of infection, expulsion and pregnancy (including ectopic)

## Slide 60
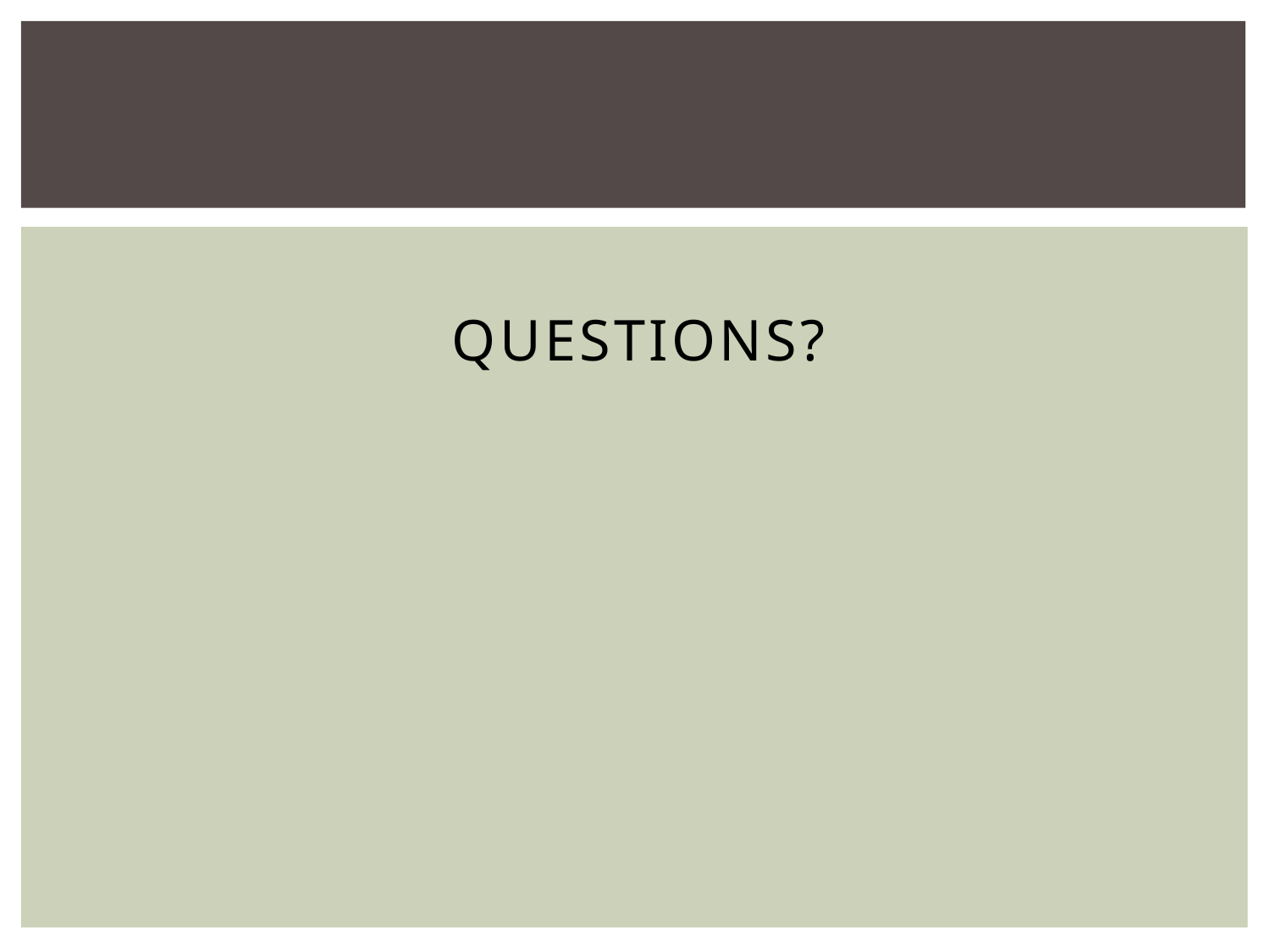

# Questions?
