# Supplementary material for: Intrauterine Device Training Workshop for Preclinical Medical Students
Source: MedEdPORTAL. 2019 Oct 18;15:10841. doi: 10.15766/mep_2374-8265.10841 (PMC6944262; doi:10.15766/mep_2374-8265.10841)
Supplement: Supplementary file 1 — A. Student Pretest Survey.docx B. IUD Simulation PowerPoint Didactic.pptx C. Student Posttest Survey.docx D. Faculty Guide for IUD Workshop.docx [file mep-15-10841-s001.zip › C. Student Posttest Survey.docx]

**Post-Training Intrauterine Contraception Questionnaire**

1. Please provide the last 4 digits of your cell phone (or home phone) number. _____________.

This is used to pair your pre- and post- survey responses. It is not used to identify you in any way.

1. If 100 women used any type of IUD for 1 year, how many would have an unintended pregnancy?
   1. <1%
   2. 1-2%
   3. 3-5%
   4. >5%
2. How quickly does fertility typically return after any type of IUD is removed?
   1. Within 1 month
   2. 1-2 months
   3. 3-6 months
   4. 6-12 months
3. What is the primary mechanism of action of a levonorgestrel IUD in contraception?
   1. preventing fertilization
   2. preventing implantation
   3. disrupting an implanted embryo
4. What is the primary mechanism of action of a copper IUD in contraception?
   1. preventing fertilization
   2. preventing implantation
   3. disrupting an implanted embryo

|  | 1 | 2 | 3 | 4 | 5 |
| --- | --- | --- | --- | --- | --- |
|  | Strongly Agree | Agree | Neither Agree nor Disagree | Disagree | Strongly Disagree |
| **The following clinicians should be able to COUNSEL patient about IUDs:** |  |  |  |  |  |
| Ob-Gyns |  |  |  |  |  |
| Family Physicians |  |  |  |  |  |
| Internal Medicine Physicians |  |  |  |  |  |
| Pediatricians |  |  |  |  |  |
| Midwives/Nurse Practitioners |  |  |  |  |  |

|  | 1 | 2 | 3 | 4 | 5 |
| --- | --- | --- | --- | --- | --- |
|  | Strongly Agree | Agree | Neither Agree nor Disagree | Disagree | Strongly Disagree |
| **The following clinicians should be able to PLACE IUDs:** |  |  |  |  |  |
| Pediatrician |  |  |  |  |  |
| Midwives/Nurse Practitioner |  |  |  |  |  |
| Internal Medicine  Physicians |  |  |  |  |  |
| Ob-Gyns |  |  |  |  |  |
| Family Physicians |  |  |  |  |  |

|  | 1 | 2 | 3 | 4 | 5 |
| --- | --- | --- | --- | --- | --- |
|  | Strongly Agree | Agree | Neither Agree nor Disagree | Disagree | Strongly Disagree |
| I am interested in learning more about IUDs |  |  |  |  |  |
| It was worthwhile to learn how to place IUDs |  |  |  |  |  |
| Contraceptive counseling will be a part of my practice |  |  |  |  |  |
| The IUD simulation increased my interest in women’s health |  |  |  |  |  |
| The IUD simulation increased my interest in contraceptive care |  |  |  |  |  |
| **Assuming she is an otherwise good candidate, I would recommend an IUD for a patient:** |  |  |  |  |  |
| Who has never been pregnant |  |  |  |  |  |
| Who is under 19 years old |  |  |  |  |  |
| Who currently has chlamydia |  |  |  |  |  |
| Who has had more than 1 vaginal delivery |  |  |  |  |  |

|  | 1 | 2 | 3 | 4 | 5 |
| --- | --- | --- | --- | --- | --- |
|  | Strongly Agree | Agree | Neither Agree nor Disagree | Disagree | Strongly Disagree |
| I am able to counsel patient about the IUD |  |  |  |  |  |
| I know as much about the IUD as other contraceptive methods |  |  |  |  |  |
| I know the steps to place an IUD |  |  |  |  |  |
| I feel comfortable placing an IUD independently in plastic model |  |  |  |  |  |
| I could teach another student how to place an IUD in a plastic model |  |  |  |  |  |
| I feel comfortable placing an IUD in a patient under faculty supervision |  |  |  |  |  |

End of Post-Test

*With permission from the authors, portions of this survey have been used from a previously published manuscript (Bartz, et al).
