# Supplementary material for: Intrauterine Device Training Workshop for Preclinical Medical Students
Source: MedEdPORTAL. 2019 Oct 18;15:10841. doi: 10.15766/mep_2374-8265.10841 (PMC6944262; doi:10.15766/mep_2374-8265.10841)
Supplement: Supplementary file 1 — A. Student Pretest Survey.docx B. IUD Simulation PowerPoint Didactic.pptx C. Student Posttest Survey.docx D. Faculty Guide for IUD Workshop.docx [file mep-15-10841-s001.zip › D. Faculty Guide for IUD Workshop.docx]

Appendix D: Faculty Guide

Intrauterine Device Training Workshop for Preclinical Medical Students

Supplies:

1. Computer and/or projector for powerpoint slides
2. Plastic uterus models provided by IUD manufacturer (or papaya model^18^ as previously described)
3. IUD demonstration kits provided by IUD manufacturer
   1. CuT 380A (ParaGard, CooperSurgical, Trumbell, CT)
   2. Levonorgestrel-containing intrauterine devices (Mirena, Kyleena and Skyla, Bayer HealthCare Pharmaceuticals, Inc; and Liletta, Allergan/Medicines 360)
   3. Donated placebo IUD kits can be obtained by contacting the respective manufacturers if these are otherwise not available at your facility
4. Long suture scissors
5. Uterine sounds
6. Tenaculums
7. Gloves
8. Sign-in sheet for students (optional)
9. Copies of pre- and post-test surveys (optional)

Faculty set-up list:

- Set up computer for powerpoint slide show for Part 1
- Set up stations with each IUD type and plastic discs in the shape of a uterus for Part 2, may depend on number of participants or number of sample IUDs available
- If plastic discs in the shape of a uterus are not available, can use papaya as previously described
- Obtain residents/faculty volunteers for the workshop who can help students troubleshoot IUD placement and are familiar with the different types of IUDs

Overview of the curriculum:

Part 1: 45 minute lecture “Everything you need to know about intrauterine devices (IUDs)”

- See Appendix B for power point slides
- Slides have been annotated to include talking points that may be helpful for faculty
- This didactic portion covers the following topics:
  - Contraception Use in the United States
  - Types of IUDs
  - Benefits of IUDs
  - Debunking IUD Myths
  - Risks of IUD
  - Cost effectiveness

Part 2: 45 minute IUD simulation training (hands-on)

- Utilize slides #45 – 60 to review IUD placement steps
- This portion of the simulation includes a detailed review on the steps of IUD placement, potentially including:
  - Consent
  - Pre-procedure urine HCG or other lab testing such as GC/CT
  - Bimanual exam
  - Speculum exam
  - Cervical preparation with betadine or chlorhexidine solution
  - Placement of the tenaculum for cervical stabilization
  - Tips for multiparous or nulliparous women: potential use of local anesthesia, peri-procedure NSAIDs
- Procedure for placement of various IUDs (may depend on models available) and trimming strings
  - CuT 380A (ParaGard, CooperSurgical, Trumbell, CT)
  - Levonorgestrel-containing IUDs (Mirena, Kyleena and Skyla, Bayer HealthCare Pharmaceuticals, Inc. and Liletta, Allergan/Medicines 360)

Pre- and Post-Test Surveys

- If utilizing the pre- and post-test surveys, ensure students are aware that this is portion of the workshop is optional, but data is de-identified and will not negatively affect them in any way.
- Pre- and post-test surveys were linked using a 4-digit number each student created and wrote on the top of the survey.
- Ensure enough papers copies for each student to have both a pre- and post-test survey. We used two different colors for the pre- and post-test survey to keep the papers separate.
- Hand out the pre-test surveys as students are walking in and collect all of the completed surveys before starting the workshop.
- Near the end of the session, begin to pass out the post-test surveys to each student. Ensure that there is a person or a box where completed surveys can be left before students begin leaving.
